# KEGG orthology-based annotation of the predicted proteome of *Acropora digitifera*: ZoophyteBase - an open access and searchable database of a coral genome

**DOI:** 10.1186/1471-2164-14-509

**Published:** 2013-07-26

**Authors:** Walter C Dunlap, Antonio Starcevic, Damir Baranasic, Janko Diminic, Jurica Zucko, Ranko Gacesa, Madeleine JH van Oppen, Daslav Hranueli, John Cullum, Paul F Long

**Affiliations:** 1 Centre for Marine Microbiology and Genetics, Australian Institute of Marine Science, PMB No. 3 Townsville MC, Townsville 4810, Queensland, Australia; 2 Institute of Pharmaceutical Science, King’s College London, Franklin-Wilkins Building, 150 Stamford Street, London SE1 9NH, United Kingdom; 3 Department of Chemistry King’s College London, Franklin-Wilkins Building, 150 Stamford Street, London SE1 9NH, United Kingdom; 4 Section for Bioinformatics, Department of Biochemical Engineering, Faculty of Food Technology and Biotechnology, University of Zagreb, Pierottijeva 6, 10000 Zagreb, Croatia; 5 Department of Genetics, University of Kaiserslautern, Postfach 3049, 67653 Kaiserslautern, Germany

**Keywords:** *Acropora digitifera*, KEGG orthology, Database, Annotation, Proteome, Genome, Coral, Symbiosis, Cnidaria

## Abstract

**Background:**

Contemporary coral reef research has firmly established that a genomic approach is urgently needed to better understand the effects of anthropogenic environmental stress and global climate change on coral holobiont interactions. Here we present KEGG orthology-based annotation of the complete genome sequence of the scleractinian coral *Acropora digitifera* and provide the first comprehensive view of the genome of a reef-building coral by applying advanced bioinformatics.

**Description:**

Sequences from the KEGG database of protein function were used to construct hidden Markov models. These models were used to search the predicted proteome of *A. digitifera* to establish complete genomic annotation. The annotated dataset is published in ZoophyteBase, an open access format with different options for searching the data. A particularly useful feature is the ability to use a Google-like search engine that links query words to protein attributes. We present features of the annotation that underpin the molecular structure of key processes of coral physiology that include (1) regulatory proteins of symbiosis, (2) planula and early developmental proteins, (3) neural messengers, receptors and sensory proteins, (4) calcification and Ca^2+^-signalling proteins, (5) plant-derived proteins, (6) proteins of nitrogen metabolism, (7) DNA repair proteins, (8) stress response proteins, (9) antioxidant and redox-protective proteins, (10) proteins of cellular apoptosis, (11) microbial symbioses and pathogenicity proteins, (12) proteins of viral pathogenicity, (13) toxins and venom, (14) proteins of the chemical defensome and (15) coral epigenetics.

**Conclusions:**

We advocate that providing annotation in an open-access searchable database available to the public domain will give an unprecedented foundation to interrogate the fundamental molecular structure and interactions of coral symbiosis and allow critical questions to be addressed at the genomic level based on combined aspects of evolutionary, developmental, metabolic, and environmental perspectives.

## Background

All of the reef-building corals (Scleractinia; phylum Cnidaria) that create the vast calcium carbonate deposits of coral reefs have evolved an endosymbiotic partnership with photosynthetic dinoflagellates of the genus *Symbiodinium* (Dinophyceae), commonly known as zooxanthellae, which reside within the gastrodermal cells of their scleractinian host [1-3]. Coral-algal symbiosis is a cooperative metabolic adaptation necessary for survival in the shallow oligotrophic (nutrient-poor) waters of tropical and subtropical marine environments [4,5] that drives the productivity of coral reefs [6]. Coral reefs provide habitat and trophic support for many thousands of marine species, the richness of which rival the biological biodiversity of tropical rainforests [7]. Underlying the basic requirements of corals for growth, reproduction and survival are special needs to accommodate symbiont-specific host recognition, to control innate and responsive immune systems, and what is likely to emerge from future research is the extent to which the host is involved in direct regulation of its endosymbiont populations. Much is understood about the cellular biology of cnidarian-dinoflagellate symbiosis (reviewed in [8]), but less is known at the molecular level of coral symbiology. There is little opposition to the contention that environmental and anthropogenic disturbances are causing alarming losses to coral reefs ([9] and reference therein). Threats to productivity are being imposed by the disruption of coral symbiosis (apparent as “coral bleaching”) caused in response to increasing thermal stress attributed to global warming [10,11], from an increase in stress-related coral disease [12-14], from the discharge of domestic and industrial wastes, pollutants from agricultural development and the transport of sediments in terrestrial runoff [15,16], and potentially from imminent declines in coral calcification owing to rising ocean acidification [17-19]. Accordingly, we require a better understanding of the molecular stress responses and adaptive potential of corals. Such information is necessary to predict bleaching events and so better inform effective management policies for the conservation of coral reef ecosystems [20-24].

To understand how coral holobionts respond to environmental change at the molecular level, the identification of genes that may respond by transcription to stress is of primary importance [25]. Thus, the use of transcriptomic methodologies to identify stress-responsive genes has been highly successful [26-32]. Transcriptome high-throughput profiling has allowed changes in gene expression across thousands of genes to be measured simultaneously. Fuelled by data-generating power, the number of coral based studies utilising transcriptomics to investigate molecular responses to environmental stressors has expanded greatly by the acquisition of expressed sequence tag (EST) gene libraries, the fabrication of microarray biochips used to estimate levels of mRNA expression, and by direct analysis using next-generation, high-throughput sequencing. However, much of this work has been conducted using the aposymbiotic state of pre-settlement coral larvae, so transcribed genes relevant to metamorphosis and the cytobiology of the adult polyp are limited to a few recent studies [33-36]. The transcriptome additionally does not provide the structural framework and essential regulatory elements of the functional genome for comprehensive evaluation. Recently, deep metatranscriptomic sequencing of two adult coral holobiomes has been made available on searchable databases: PocilloporaBase for *Pocillopora damicornis*[36] and PcarnBase for *Platygyra carnosus*[37]. In contrast, high-throughput metaproteomic analyses to quantify the product yield of stress-response genes of the coral holobiome are yet to be widely adopted by the coral reef scientific community, despite the proteome being the ultimate measure of the coral phenotype [38,39].

The early accumulation of transcriptomic data revealed that a small proportion of coral ESTs matched genes known previously only from other kingdoms of life, implying that the ancestral animal genome contained many genes traditionally regarded as ‘non-animal’ that have been lost from most animal genomes [40]. Furthermore, an unexpected revelation from EST data is the greater extent to which coral sequences resemble human genes than those of the *Drosophila* and *Caenorhabditis* model invertebrate genomes [41,42]. Comparative genomic analysis has revealed higher genetic divergence and massive gene loss within the ecdysozoan lineages. Hence, many genes assumed to have much later evolutionary origins are likely to have been present in an ancestral or early-diverged metazoan [43]. While much of the animal kingdom remains yet to be explored, examples of the metazoan phylum Cnidaria provide a unique insight into the deep evolutionary origins of at least some vertebrate gene families [42]. Thus, the complete genomic sequence of a coral is likely to reveal many genes previously assumed to be strictly vertebrate innovations. To date, cnidarian genomes have been published for the sea anemone *N. vectensis*[42] and the hydroid *Hydra magnipapillata*[44]. Only the coral genome of *Acropora digitifera* is available without restriction on use of its published sequence [45], but the compiled sequence has not been fully annotated. At the time of this writing, the genome assembly of *Acropora millepora* has been released to the public domain [46], also without full annotation, but an embargo is imposed on use of this data that is highly restrictive to the progress of further studies. Understanding how genomic variation affects molecular and organismal biology is the ultimate justification of genome sequencing, and annotation is an essential step in this process. We envisage that unrestricted access to annotation of the *A. digitifera* genome will provide an unprecedented foundation to freely interrogate the generic molecular structure, possible endobiotic interactions and the response of coral to environmental stress. Accordingly, we offer annotation of the predicted proteome of *A. digitifera* on the open access and searchable database, ZoophyteBase [47]. Use of the ZoophyteBase search engines will allow genes of encoded proteins to be identified that can be examined in context of the cellular physiology, processes of ecological significance, the evolutionary and developmental biology of corals and the functional metabolism of the holobiont that collectively underpin the health of coral reefs.

## Construction and content

ZoophyteBase is an open access and searchable database of complete annotation of the predicted proteome of the coral *A. digitifera*[48]. It was constructed using the MEGGASENSE system, which is a general system for constructing annotation databases with different sorts of input data (DNA reads, assembled genomes, predicted proteomes) and the possibility of using different combinations of analysis tools to create the annotation (Gacesa et al, in preparation). In the case of ZoophyteBase, hidden Markov model (HMM) profiles [49] were chosen as the annotation tool rather than the more common BLAST searches [50]. HMM profiles are constructed from multiple alignments of protein families and contain information about conserved differences in amino acid residues as well as deletions and insertions [49]. This is particularly important for a coral database, as corals are evolutionarily distant to most other organisms. This means that known homologous sequences present in the databases will usually have relatively low similarity, making BLAST searches inaccurate. The statistical information in an HMM profile gives more sensitive and accurate detection of sequence homology. An additional advantage of HMM profiles is that the statistical significance of hits (the expected value) is much more accurate than that calculated by BLAST programs.

The quality of sequence annotation is limited by the accuracy of information provided in any database used. It is well known that there are many problems with annotation in the large uncurated databases such as the NCBI GenBank nr sequences. Widely accepted, the most accurate database for functional annotation is the KEGG database [51]. The KEGG database organises sequences as groups of KEGG orthologues. These are sets of homologous sequences from as wide a range of organisms as possible having an assigned molecular function. These functions are arranged in a hierarchical fashion and grouped in biological pathways. The sequences belonging to KEGG orthologues were used to construct HMM profiles for annotating the coral sequences. Accordingly, the 23,524 predicted proteins encoded in the coral genome were analysed using HMM profiles. If a protein showed a highly significant correlation (“hit”) to a single HMM profile, this was used to create a “trusted” annotation of the sequence. Choosing a cut-off for this criterion is not trivial, because longer sequences tend to have more significant e-values. For construction of ZoophyteBase the criterion 1e-5 was used. This resulted in 19,044 predicted proteins giving “trusted” sequence annotation. For many of these proteins there were two or more highly significant hits to established HMM profiles. In these cases, the most significant correlation was used to construct our “best-fit” annotation file, but other hits can be viewed by the database user so that expert knowledge can be employed to override the automatic annotation function. In 8,004 out of 19,044 predicted proteins which were annotated, more than one annotation was assigned based on non-overlapping regions within the protein which were used to construct the “best-fit” annotation file. We interpreted these as “fusion” events generated by the *in silico* protein prediction method used, and these proteins were treated as multiple instead of single encoded proteins. Hence, this analysis resulted in the annotation of 33,195 proteins in total, generated from the original 23,524 predicted coral proteins. This is a very conservative annotation scheme, so it can be assumed that most of the annotations are biologically meaningful. Almost 81% (19,044 out of 23,524) of the predicted proteome was assigned using this method.

## Utility

**Figure 1:**
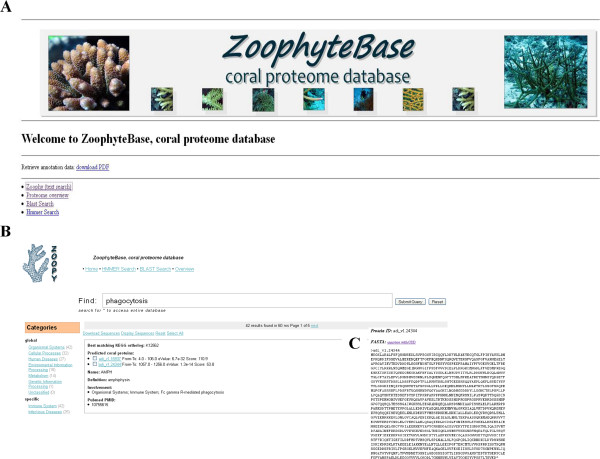
**Graphical overview of the user-web interface for ZoophyteBase during a typical search.** The home page allows several search functions **(A)**. Text queries using an intelligent Google-like search engine is illustrated by using the query “phagocytosis” **(B)**. This finds 42 hits to KEGG orthologue profiles. One of the hits corresponds to amphiphysin with annotation of two protein homologues encoded in the coral genome. On the data page there is a brief description of the function of amphiphysin together with a PUBMED literature reference. The sequences of the predicted coral proteins can be retrieved **(C)**.

The MEGGASENSE system was used to generate a web interface for ZoophyteBase. The home page (Figure 1A) allows the use of several functions. A text version of the entire annotation can be downloaded for manual inspection. There is a proteome overview that gives statistics about the database and a breakdown of the annotated functions into different categories of genes. A particularly useful feature of ZoophyteBase is the ability to use text queries employing a search engine that provides a relevant inquiry in the absence of an exact match between key words of a search and those described for a functional protein. The search engine uses text from the KEGG-database, PubMed and other sources to establish links between query words to access protein data using an intelligent Google-like search engine implemented by the search platform Lucene/Solr [52]. This helps to overcome the common problem that different terminology is used by different groups of researchers. The use of this search function is illustrated by using the query “phagocytosis” (Figure 1B). This inquiry finds 42 hits to KEGG orthologue profiles. One of the hits corresponds to amphiphysin (a synaptic vesicle protein) with annotation of two protein homologues encoded in the coral genome. On the data page there is a brief description of the function of amphiphysin together with a PUBMED literature reference. The sequences of the predicted coral proteins (Figure 1C) can be retrieved, and it is also possible to analyse such data with computer aided drug design methods [53] to look for conserved domains. There are also two tools for the user to examine matches to protein sequences. The user can carry out a BLAST search against the coral protein sequence or analyse the predicted sequence against HMM profiles used to annotate the coral proteome. These tools require only the user to paste their queury into the sequence window.

In this manuscript we demonstrate the utility of ZoophyteBase by presenting predicted gene-encoded proteins revealed by annotation of the *A. digitifera* genome that have physiological, biological and environmental significance. We discuss features of importance in coral physiology: (1) regulatory proteins of symbiosis, (2) planula and early developmental proteins, (3) neural messengers, receptors and sensory proteins, (4) calcification and Ca^2+^-signalling proteins, (5) plant-derived proteins, (6) proteins of nitrogen metabolism, (7) DNA repair proteins, (8) stress response proteins, (9) antioxidant and redox-protective proteins, (10) proteins of cellular apoptosis, (11) microbial symbioses and pathogenicity proteins, (12) proteins of viral pathogenicity, (13) toxins and venom, (14) proteins of the chemical defencesome and (15) coral epigenetics.

## Discussion

### Regulatory proteins of symbiosis

Metabolic cooperation is a key feature of coral-algal symbiosis that allows reef-building corals to inhabit the often nutrient-poor waters of tropical oceans [54]. In this phototropic symbiosis, fixed carbon produced by resident algae is released to the host for nutrition, and the algal symbionts benefit by acquiring the inorganic nutrient wastes of host metabolism [2,55]. The symbiotic dinoflagellates reside and proliferate within a specialised phagosome (the symbiosome) maintained within host gastrodermal cells. This arrangement requires complex biochemical coordination by the coral at various metabolic stages that includes endocytosis (phagocytosis) by post-settlement polyps to acquire algal symbionts, accord symbiosome recognition to arrest phagosomal maturation for sustained organelle homeostasis, activate symbiophagy or exocytosis to eliminate damaged symbionts [56,57], and regulate apoptotic or exocytotic pathways to remove excess or impaired populations, all of which have long been recognised as essential to preserve the stability of coral symbiosis [58]. Although these processes are poorly understood in corals, it has been realised from studies of the sea anemone *Aiptasia pulchella*, a related anthozoan also containing *Symbiodinium* sp. endosymbionts, that the persistence of algal-containing symbiosomes in Cnidaria relies on the exclusion or retention of small Rab GTPase family proteins that are key regulatory components of vesicular trafficking and membrane fusion in eukaryotic cells [59]. Significantly, ApRab3 and ApRab4 accumulate in the biogenesis of maturing symbiosomes of *A. pulchella*[60,61], and mature symbiosomes enveloping healthy dinoflagellates have tethered ApRab5 [62], a checkpoint antagonist of downstream ApRab7 and ApRab11 proteins that would otherwise direct autophagy of the symbiont cargo [63,64].

Our annotation of the *A. digitifera* genome reveals sequences encoding putative Rab homologues of the Ras superfamily of proteins (Table 1). In a comparison of cnidarian Rab proteins, eight proteins of *A. digitifera* matched homologues of *Aiptasia pulchella*, twenty-nine matched proteins encoded by the aposymbiotic freshwater *H. magnipapillata* and the aposymbiotic anemone *N. vectensis* genomes, while seven Rab and Rab-interacting proteins of *A. digitifera* did not match other cnidarian proteins (Table 2). Significantly, the eight homologues of *A. digitifera* that matched exclusively Rab proteins of *A. pulchella* included homologues of the aforementioned ApRab3, ApRab4 and ApRab5 proteins attributed to the maintenance of healthy symbiosomes in *Aiptasia*, while homologues of the autophagic ApRab7 and ApRab11 proteins are found also in *N. vectensis*. While Rab GTPase proteins and their effector proteins coordinate consecutive stages of endocytic vesicular transport [65,66], soluble N-ethylmaleimide-sensitive factor attachment receptor (SNARE) proteins are essential for Rab assembly to complete endosomal fusion of vesicle membranes [67], a process by which Rab proteins impart specificity by binding distinct Rab and SNARE partner proteins prior to membrane fusion [68]. Genes encoding syntaxin-like SNARE proteins have been unambiguously identified [69] from coral EST database libraries constructed from expressed mRNA isolated from various early life stages of *Acropora aspera*, *A. millepora*, *A. palmata* and *Orbicella faveolata* (= *Monastraea faveolata*), as well as from the genome of the sea anemone *N. vectensis*[70]. In metazoans, vacuolar r-SNARE receptor proteins comprise the syntaxin, synaptobrevin and VAMP family proteins, of which there are eight syntaxin and syntaxin-binding proteins (plus two plant-like syntaxins). Additionally, there are one t-SNARE target protein to direct vacuolar morphogenesis, two synaptosomal proteins, one synaptosomal complex ZIP1 protein (yeast homologue), one synaptobrevin membrane protein of secretory vesicles, ten vesicle-associated membrane proteins (VAMPs), a vacuolar protein-8 regulator of autophagy, four vacuolar-sorting proteins and two SEC22 vesicle trafficking protein encoded in the genome of *A. digitifera* (Table 1), many of which may interact to provide metabolic transport between the endoplasmic reticulum and Golgi apparatus [71]. Included in this vast but yet unexplored repertoire of vacuolar-acting proteins are the syntaxin-binding amisyn and tomosyn regulators of SNARE complex assembly and disassembly [72,73], which may control membrane fusion in the phagocytic establishment and dis-sociation of coral symbiosis.

**Table 1:** **Regulatory proteins of symbiosis in the predicted proteome of *A. digitifera***

**Gene sequence**	**KEGG Orthology**	**Encoded protein description**
v1.06849	K06110	Exocyst complex component 3
v1.00063; v1.01826	K06111	Exocyst complex component 4
v1.06336; v1.06337; v1.15354	K07195	Exocyst complex component 7
v1.04340 [+ 4 other sequence copies]	K14966	Host cell factor
v1.01629; v1.19166	K12481	Rabenosyn-5
v1.18447 [+ 26 other sequence copies]	K07976	Rab family, other (similar to Rab-6B)
v1.02380	K12480	Rab GTPase-binding effector protein-1
v1.01032	K13883	Rab-interacting lysosomal protein
v1.14682; v1.03256; v1.07709	K12484	Rab11 family-interacting protein-1/2/5
v1.13055; v1.13176; v1.16348	K12485	Rab11 family-interacting protein-3/4
v1.01275	K07932	Rab-like protein-2B
v1.17629 [+ 13 other sequence copies]	K07933	Rab-like protein-3
v1.03299; v1.09653	K07934	Rab-like protein-4
v1.08498	K07935	Rab-like protein-5
v1.16155 [+5 other sequence copies	K07874	Ras-related protein Rab-1A
v1.09098	K07875	Ras-related protein Rab-1B
v1.13558; v1.08983	K07877	Ras-related protein Rab-2A
v1.14260	K07878	Ras-related protein Rab-2B
v1.07500; v1.20532; v1.07498	K07884	Ras-related protein Rab-3D
v1.21242; v1.07502	K07880	Ras-related protein Rab-4B
v1.01341; v1.05619	K07888	Ras-related protein Rab-5B
v1.07125	K07889	Ras-related protein Rab-5C
v1.09239	K07893	Ras-related protein Rab-6A
v1.10443; v1.13335	K07897	Ras-related protein Rab-7A
v1.03086; v1.17122; v1.07231	K07916	Ras-related protein Rab-7 L1
v1.02275 [+ 4 other sequence copies]	K07901	Ras-related protein Rab-8A
v1.24612	K07899	Ras-related protein Rab-9A
v1.00411	K07900	Ras-related protein Rab-9B
v1.10697; v1.01515	K07903	Ras-related protein Rab-10
v1.22278; v1.04408; v1.12528	K07905	Ras-related protein Rab-11B
v1.07033; v1.23028	K07881	Ras-related protein Rab-14
v1.02275	K07908	Ras-related protein Rab-15
v1.16455; v1.14911; v1.14959	K07910	Ras-related protein Rab-18
v1.04714	K07911	Ras-related protein Rab-20
v1.01878; v1.12184	K07890	Ras-related protein Rab-21
v1.09930	K06234	Ras-related protein Rab-23
v1.13579; v1.12841	K07912	Ras-related protein Rab-24
v1.10183	K07913	Ras-related protein Rab-26
v1.08199	K07885	Ras-related protein Rab-27A
v1.13978; v1.18893	K07917	Ras-related protein Rab-30
v1.03085; v1.06007; v1.07729	K07918	Ras-related protein Rab-32
v1.24721	K07919	Ras-related protein Rab-33A
v1.18892	K07920	Ras-related protein Rab-33B
v1.16060	K07876	Ras-related protein Rab-35
v1.15894	K07922	Ras-related protein Rab-36
v1.03080	K07923	Ras-related protein Rab-38
v1.21391	K07924	Ras-related protein Rab-39A
v1.14786	K07928	Ras-related protein Rab-40
v1.05611 [+ 13 other sequence copies]	K08502	Regulator of vacuolar morphogenesis (t-SNARE domain)
v1.18253	K08520	SEC22 vesicle trafficking protein A/C
v1.15499	K13814	t-SNARE domain-containing protein 1
v1.05749	K08516	Synaptobrevin homologue YKT6
v1.13229	K12768	Synaptonemal complex protein ZIP1
v1.16533; v1.17141	K08508	Synaptosomal-associated protein, 23 kDa
v1.05301	K08509	Synaptosomal-associated protein, 29 kDa
v1.19071	K04560	Syntaxin 1A
v1.04614; v1.22747	K08486	Syntaxin 1B/2/3
v1.16462	K08490	Syntaxin 5
v1.20758; v1.21534	K08498	Syntaxin 6
v1.22836; v1.15499	K08488	Syntaxin 7
v1.01959; v1.24227	K08501	Syntaxin 8
v1.02007; v1.06683; v1.12727	K08491	Syntaxin 17
v1.21308; v1.11830; v1.01582	K08492	Syntaxin 18
v1.22100; v1.09457	K08518	Syntaxin binding protein 5 (tomosyn)
v1.18555	K08519	Syntaxin binding protein 6 (amisyn)
v1.12938	K08500	Syntaxin of plants SYP6
v1.06575	K08506	Syntaxin of plants SYP7
v1.14699	K08507	Unconventional SNARE in the endoplasmic reticulum protein 1
v1.23782 [+ 38 other sequence copies]	K08332	Vacuolar protein 8
v1.15282; v1.24603; v1.01672	K12196	Vacuolar protein-sorting-associated protein 4
v1.17791 [+ 4 other sequence copies]	K12479	Vacuolar protein sorting-associated protein 45
v1.20907	K11664	Vacuolar protein sorting-associated protein 72
v1.15996 [+ 5 other sequence copies]	K12199	Vacuolar protein sorting-associated protein VTA1
v1.15614	K08510	Vesicle-associated membrane protein 1 (synaptobrevin)
v1.13353	K13504	Vesicle-associated membrane protein 2 (synaptobrevin)
v1.12458; v1.07528	K13505	Vesicle-associated membrane protein 3 (cellubrevin)
v1.19735; v1.21831; v1.07186	K08513	Vesicle-associated membrane protein 4 (Golgi transport)
v1.05299	K08514	Vesicle-associated membrane protein 5 (exocytosis)
v1.13557; v1.24610	K08515	Vesicle-associated membrane protein 7 (exocytosis)
v1.12279	K08512	Vesicle-associated membrane protein 8 (endobrevin)
v1.00261; v1.08699; v1.04334	K06096	Vesicle-associated membrane protein A
v1.20177	K10707	Vesicle-associated membrane protein B
v1.15472; v1.03568	K06027	Vesicle-fusing ATPase
v1.11431; v1.10487	K08517	Vesicle transport protein SEC22
v1.06393; v1.13003; v1.08735; v1.04261	K08493	Vesicle transport interaction with t-SNAREs 1

**Table 2:** **Distribution of Rab homologues of *Aiptasia puchella*, *Hydra magnipapillata *and *Nematostella vectensis *in the predicted proteome of *A. digitifera***

***A. digitifera*Rab protein**	**Cnidarian encoding Rab homologue**
Rab-like protein- 2B, Rab-2B Rab-3D, Rab-4B, Rab-5B, Rab-26, Rab-32, Rab-38	*A. puchella*
Rab-like protein-3, Rab-36	*N. vectensis*
Rab-2A, Rab-23	*A. puchella*, *H. magnipapillata*
Rab-like protein-6B, Rab-6A, Rab-7 L1, Rab-10, Rab11B, Rab-30, Rab-33B	*A. puchella*, *N. vectensis*
Rab effector protein-1, Rab11-interacting protein-3/4	*H. magnipapillata*, *N. vectensis*
Rab-like protein-4, Rab-like protein-5, Rab-1A, Rab5C, Rab-7A, Rab-8A, Rab-9A, Rab-14, Rab-18, Rab-20, Rab-21, Rab-24, Rab-27A, Rab-35	*A. puchella*, *H. magnipapillata, N. vectensis*
Rab-interacting lysomal protein, Rab11-interacting protein-1/2/5, Rab-1B, Rab-9B, Rab-3A, Rab-39A, Rab-40	No match

In the final step of exocytosis there is a cytosolic influx of calcium which binds to synaptotagmin to actuate completion of membrane SNARE protein assembly with exocytic docking to form the conducting channel for trans-membrane vesicular transport on activation by vesicle-fusing ATPase [74]. As synaptotagmin proteins are not included in the KEGG database, Zoophytebase was used for BLAST searches with all known synaptotagamin sequences [27]. Synaptotagamin proteins from *A. digitifera* were found having similarity to homologues from diverse invertebrate and vertebrate organisms, including one from the human genome (Table 3). Other Ca^2+^-sensing proteins of *A. digitifera*, such as calmodulin and the calcium binding protein CML, are given with calcification and Ca^2+^-signalling proteins.

**Table 3:** **Synaptotagmin proteins in the predicted proteome of *A. digitifera***

		
**Gene sequence**	**GenBank Accession**	**Genome encoded homologue**
v1.08623	GI:268530614	*Caenorhabditis briggsae*: XP_002630433 (worm)
v1.20682; v1.10560; v1.02080; v1.10015	GI:150416761	*Platynereis dumerilii*: ABR68850 (worm)
v1.10269; v1.04412	GI:288869516	*Nasonia vitripennis*: NP_001165865 (wasp)
v1.01508	GI:29378331	*Lymnaea stagnalis*: AA093847 (snail)
v1.18613	GI:391339919	*Metaseiulus occidentalis*: XP_003744294 (mite)
v1.07402	GI:260834895	*Branchiostoma floridae*: XP_002612445 (lancelet)
v1.01542	GI:149067023	*Rattus norvegicus*: EDM16756 (rat)
v1.20683	GI:383860584	*Megachile rotundata*: XP_003705769 (bee)
v1.17688	GI:48529130	*Oreochromis niloticus*; XP_003452067 (fish)
v1.15777; v1.14902	GI:269785031	Saccoglossus kowalevskii: NP_001161667 (worm)
v1.17175; v1.11521	GI:11559313	*Halocynthia roretzi*: BAB18864 (ascidian)
v.1.03344; v1.03345	GI:12658419	*Manduca sexta*; AF331039 (moth)
v1.16152	GI:395729192	*Pongo abelii*: XP_003780414 (orangutan)
v1.10268	GI:327283049	*Anolis carolinensis*: XP_003226254 (lizard)
v10.2778	GI:125984480	*Drosophila pseudoobscura* XP_001356004.1 (fly)
v1.02083; v1.02777	GI:226490194	*Schistosoma japonicum*: CAX69339.1 (fluke)
v1.04326	GI:167744962	*Homo sapiens*: 2R83_A (human)
v1.14682; v1.04180	GI:241704658	*Ixodes scapularis*: XP_002411967 (tick)

Intriguingly, annotation of the *A. digitifera* genome reveals a host cell factor (K14966), but this is not related to the elusive “host factor” of symbiosis demonstrated to be present in tissue homogenates of corals and other marine invertebrates that harbor *Symbiodinium* spp. endosymbionts [75-77]. Instead, this mammalian transcriptional coactivator host cell factor (HFC-1) is known to mediate the enhancer-promoter assemblies of herpes simplex (HSV) and varicella zoster (VZV) viruses for activation of the latent state for replication [78], such that the coral HCF homologue may have similar relevance as a viral checkpoint transcriptional coactivator of virulence in *A. digitifera*. HCF-1 expression is coupled also to chromatin modification [79,80] suggesting that the coral protein homologue may have an additional role in epigenetic reprogramming of the chromatin histone-DNA complex at different stages of development.

### Planula and early developmental proteins

In this section we discuss predicted proteins encoded in the *A. digitifera* genome having functional homology to known proteins are specific to early embryonic development, planula larvae function and morphogenesis, which are given in Table 4. Annotation of the coral genome reveals a large set of homeobox proteins involved in the regulation of anatomical development during morphogenesis. The homeobox is a highly conserved DNA sequence (homeodomain) within genes that binds to DNA in a sequence-specific manner [81] often at the promoter region of their target gene to affect transcription in the developing embryo. Amonst these transcriptional regulators, *Hox* genes are essential to metazoan development as their expressed proteins differentiate embryonic regions along the anterior-posterior axis (the Hox code) and are recognised for their contribution to the evolution of morphological diversity [82]. *Hox* genes are well characterised in cnidarians and, given their importance in embryonic development, it is not surprising that molecular evidence from the Cnidaria reveal that the genetic origins of *Hox* genes predate the cnidarian-bilaterian divergence [83-85] yet had evolved after divergence of the sponge and eumetazoan lineages [86]. *Hox* genes of cnidarians are typically located in a conserved genomic collinear cluster, which is apparent also for *A. digitifera*, whereby the order of the genes on the chromosome is the same as that of gene expression in the developing embryo. Included in our annotation are genes encoding two LIM homeobox proteins and a LIM homeobox transcription factor (Lhx) having conserved roles in neuronal development [87], which in *N. vectensis* are responsible for the development of neural networks in developing larvae and juvenile polyps [88]. Unlike *N. vectensis*[89], the coral genome expresses a homeobox BarH-like protein that in vertebrates directs neurogenesis [90]. Distinct from homeodomain proteins, but serving similar functions, are various protein activators, regulators and receptors of cellular morphogenesis. Annotation of the coral genome has revealed multiple sequence alignments to a protein homologue of the dishevelled-associated activator of morphogenesis 1 (Daam1) that initiates cytoskeleton formation via the control of actin assembly. Daam1 was found crucial for gastrulation in *Xenopus*[91], wherein Daam1 mutants of *Drosophilia* exhibit trachea defects [92], and in mammals Daam1 is highly expressed in multiple developing organs and is deemed essential for cardiac morphogenesis [93]. Similar morphogenetic genes express regulatory proteins that are necessary for vacuole biogenesis in yeasts [94]. Others express bone morphogenetic proteins (and their BMP receptors), which are potent multi-functional growth activators that belong to the transforming growth factor beta (TGFbeta) cytokine superfamily of proteins that in humans have various functions during embryogenesis, skeletal formation, neurogenesis and haematopoiesis [95]. However, since many of the homeobox and morgenetic proteins (Table 4) are homologues of proteins with functions ascribed to higher organisms, their precise function in *A. digitifera* cannot be ascertained by KEGG orthology alone.

**Table 4:** **Planula and early developmental proteins in the predicted proteome of *A. digitifera***

		
**Gene sequence**	**KEGG Orthology**	**Encoded protein description**
v1.09797; v1.11180; v1.08414	K03776	Aerotaxis receptor (oxygen sensing)
v1.07838 [+5 other sequence copies]	K07822	Archaeal flagellar protein FlaC
v1.14039; v1.11310; v1.11309	K05502	Bone morphogenetic protein 1
v1.01025; v1.17008; v1.15796; v1.23658	K04662	Bone morphogenetic protein 2/4
v1.02299; v1.07696; v1.10675	K04663	Bone morphogenetic protein 5/6/7/8
v1.06335; v1.01763	K04673	Bone morphogenetic protein receptor type-1A
v1.13481	K13578	Bone morphogenetic protein receptor type-1B
v1.10550 [+4 other sequence copies]	K04671	Bone morphogenetic protein receptor type-2
v1.00912 [+4 other sequence copies]	K13579	Bone morphogenetic protein receptor type-1, invertebrate
v1.19370	K14624	C-C motif chemokine 2
v1.23163	K12499	C-C motif chemokine 5
v1.08576	K05511	C-C motif chemokine 15/23
v1.09229	K05512	C-C motif chemokine 19/21
v1.09305	K08373	C-C chemokine receptor-like 2
v1.04942	K04179	C-C chemokine receptor type 4
v1.02658	K04245	Chemokine-like receptor 1
v1.21300	K12671	C-X-C motif chemokine 10
v1.16396; v1.21991	K10035	C-X-C motif chemokine 16
v1.23712	K11522	Chemotaxis family two-component system response regulator PixG
v1.09435	K13490	Chemotaxis family, histidine kinase sensor response regulator (WspE-like)
v1.14142; v1.05300	K05874	Chemotaxis protein I, serine sensor receptor (MCP family)
v1.07361	K05877	Chemotaxis protein IV, peptide sensor receptor (MCP family)
v1.17411	K03414	Chemotaxis protein CheZ
v1.16104	K00575	Chemotaxis protein methyltransferase CheR
v1.15537 [+ 7 other sequence copies]	K08482	Circadian clock protein KaiC
v1.14925 [+ 4 other sequence copies]	K02223	Circadian locomoter output cycles kaput protein
v1.06432 [+ 9 other sequence copies]	K04512	Dishevelled associated activator of morphogenesis
v1.17637 [+ 70 other sequence copies]	K10408	Dynein heavy chain, axonemal
v1.00202 [+5 other sequence copies]	K10409	Dynein intermediate chain 1, axonemal
v1.04986; v1.09649; v1.23645	K11143	Dynein intermediate chain 2, axonemal
v1.08695; v1.09481; v1.23153	K10411	Dynein light chain 1, axonemal
v1.11684	K10412	Dynein light chain 4, axonemal
v1.23322; v1.01131; v1.04207	K10410	Dynein light intermediate chain, axonemal
v1.14083	K02401	Flagellar biosynthetic protein FlhB
v1.16997	K02420	Flagellar biosynthetic protein FliQ
v1.02867	K02396	Flagellar hook-associated protein 1 FlgK
v1.18101; v1.13427	K02408	Flagellar hook-basal body complex protein FliE
v1.04339; v1.07633	K06603	Flagellar protein FlaG
v1.17895[+5 other sequence copies]	K02383	Flagellar protein FlbB
v1.21111	K02413	Flagellar protein FliJ
v1.17651 [+ 13 other sequence copies]	K02415	Flagellar protein FliL
v1.01971 [+ 6 other sequence copies]	K02418	Flagellar protein FliO/FliZ
v1.14031	K02423	Flagellar protein FliT
v1.08025	K02394	Flagellar P-ring protein precursor FlgI
v1.02396; v1.15777	K02409	Flagellar M-ring protein FliF
v1.20693	K09451	Homeobox protein aristaless-like 4
v1.24732 [+5 other sequence copies]	K09452	Homeobox protein aristaless-related
v1.15788; v1.19334; v1.04164	K09313	Homeobox protein cut-like
v1.01801	K09319	Homeobox protein engrailed
v1.16835; v1.06323	K09320	Homeobox even-skipped homologue protein
v1.0412; v1.054771	K09354	Homeobox protein expressed in ES cells 1
v1.13604	K09324	Homeobox protein goosecoid
v1.06346; v1.08163	K09325	Homeobox protein goosecoid-like
v1.17295; v1.17294	K09361	Homeobox protein, BarH-like (vertebrate neurogenesis)
v1.07457	K09316	Homeobox protein DLX, invertebrate
v1.11157; v1.08573; v1.15250	K09317	Homeobox protein EMX
v1.01800	K09321	Homeobox protein GBX
v1.10929; v1.06346; v1.05443; v1.07458	K09310	Homeobox protein GSH
v1.13684; v1.24444	K08025	Homeobox protein HB9
v1.16254; v1.16064	K08024	Homeobox protein HEX
v1.07458; v1.06706; v1.06705	K09339	Homeobox protein HLX1
v1.06347; v1.06348; v1.17294	K09302	Homeobox protein HoxA/B2
v1.06125	K09306	Homeobox protein HoxA/B/C6
v1.19818	K09304	Homeobox protein HoxA/B/C/D4
v1.06706	K09301	Homeobox protein HoxA/B/D1
v1.02056	K09353	Homeobox protein LBX
v1.06347; v1.06348	K09328	Homeobox protein Unc-4
v1.24342; v1.04552	K09318	Homeobox protein ventral anterior
v1.03823; v1.10070; v1.04435	K09309	Homeobox protein Nkx-1
v1.12852 [+ 4 other sequence copies]	K08029	Homeobox protein Nkx-2.2
v1.21630	K09345	Homeobox protein Nkx-2.5
v1.10625	K09347	Homeobox protein Nkx-2.8
v1.10625; v1.13865; v1.05476	K09348	Homeobox protein Nkx-3.1
v1.21628; v1.05475; v1.05477	K09995	Homeobox protein Nkx-3.2
v1.06135; v1.10071	K09349	Homeobox protein Nkx-5
v1.14702	K08030	Homeobox protein Nkx-6.1
v1.14917; v1.11907	K09350	Homeobox protein Nkx-6.2
v1.00777; v1.21453	K09322	Homeobox protein MOX
v1.00602 [+ 6 other sequence copies]	K09326	Homeobox protein OTX
v1.16722; v1.12785	K09374	LIM homeobox protein 3/4
v1.11281; v1.05135	K09375	LIM homeobox protein 6/8
v1.07988; v1.22037	K09371	LIM homeobox transcription factor 1
v1.09328 [+ 5 other sequence copies]	K10394	Kinesin family member 3/17
v1.09196; v1.12479	K11525	Methyl-accepting chemotaxis protein PixJ (MCP family)
v1.17028; v1.13473	K08473	Nematode chemoreceptor
v1.13159; v1.00655	K09330	Paired mesoderm homeobox protein 2
v1.15178; v1.10962; v1.16587; v1.01557	K02633	Period circadian protein
v1.23288; v1.13857	K04627	Pheromone a factor receptor
v1.22464; v1.17135	K11213	Pheromone alpha factor receptor
v1.05611 [+ 13 other sequence copies]	K08502	Regulator of vacuolar morphogenesis
v1.04431	K09333	Retina and anterior neural fold homeobox-like protein
v1.17636	K09331	Short stature homeobox protein
v1.14704	K09340	T-cell leukemia homeobox protein
v1.11765	*NA*^**1**^	Tektin
v1.04154	K02669	Twitching motility protein PilT

^**1**^*NA* KEGG orthology designation not assigned.

Another protein encoded in the *A. digitifera* genome is a retina and anterior neural fold homeobox-like (RAX) protein that may activate the development of primitive coral photoreceptors [96,97], including a blue light-sensing, cryptochrome photoreceptor that in *A. millepora* is implicated in the detection of light from the lunar cycle of night time illumination to signal synchronous coral spawning [98,99]. Photosensitive behaviours and the circadian rhythms of corals are well described, and diurnal cycles of gene transcription that regulate circadian biological processes in the coral *A. millepora* have been reported [100]. Such traits in *A. millepora* appear regulated by an endogenous biological clock entrained to daily cycles of solar illumination [101]. Annotation of the *A. digitifera* genome reveals a circadian timekeeper protein KaiC [102] that in cyanobacteria is activated during the diurnal phosphorylation rhythm [103,104]. In *Synechococcus elongatus*, KaiC regulates the rhythmic expression of all other proteins encoded in the genome [105], yet no homologue of any of the prokaryotic clustered circadian *kiaABC* genes has been identified in eukaryotes [106]. In *Drosophila*, KaiC together with a homologue of the eukaryotic period (Per) circadian protein drives circadian rhythms in eclosion (hatching) and locomotor activity [107]. Nevertheless, a circadian locomotor output cycles kaput (CLOCK) homologue (Table 4) was found in our annotation. Since CLOCK proteins serve as an essential activator of downstream elements in pathways critical to the regulation of circadian rhythms in eukaryotes [108], it would be worthy to examine how transcription of the RAX-like homeobox protein in this coral contributes to the development of circadian functions by activation of *kaiC*, *per* and *Clock* genes. Such a study might reveal that components of the animal circadian clock are more ancient than data previously suggested [109].

Broadcast-spawning corals, such as *A. digitifera*, release gametes, and the fertilised eggs develop into planula larvae within the water column until they have reached settlement competency, find a suitable hard substrate, attach and develop into the polyp on metamorphosis. Coral sperm and planula larvae achieve motility using flagella (sperm) or cilia (larvae) as their locomotor organelles. The eukaryotic axonemal proteins of cilia and flagella are composed of a dynein ATPase protein to provide mechanochemical energy transduction together with the principle structural proteins of the ciliary/flagellar microtubules [110]. The flagellar/ciliary microtubules consist of filaments composed of α- and β-tubulins, microtubule-stabilising tektins and kinesin motor proteins [111-113]. The coral genome encodes members of the dynein axonemal (flagella and cilia) proteins (Table 4) and many of the dynein cytoplasmic proteins (not tabulated), the latter being involved in intracellular organelle transport and centrosome assembly. The coral genome encodes α- and β-tubulins and members of the eukaryotic kinesin superfamily proteins (not tabulated). Amongst the many kinesin proteins encoded in the coral genome is the kinesin family member 3/17 protein, which is a direct homologue of the kinesin-II intraflagellar transport protein FLA10 essential for flagella assembly in the alga *Chlamydomonas*[114]. The microtubule-stabilising tektin protein, which is required for cilia and flagella assembly [113], is also encoded in the coral genome [note: there is no KEGG orthology identifier assigned to this protein]. It was a surprise, however, to find a large complement of prokaryotic flagellar proteins encoded in the coral genome consisting of archaeal flagellar (FlaC and FlaG), bacterial filament (FibB, FlliE, FliF, FliJ, FliK, FliO/FliZ, FliQ and FliT) homologue components (Table 4). Included also are the prokaryote homologues FlgN and FlbB that regulate transcriptional activation of flagellar assembly [115,116] and FlhB which controls the substrate specificity of the entire prokaryotic flagellar apparatus [117]. Encoded in the coral genome is a flagella-independent Type IV twitching mobility protein PilT that affords social gliding translocation in many prokaryotic organisms controlled by complex signal transduction systems that include two-component sensor regulators [118]. It is unlikely that these genes are derived from contamination from bacterial DNA. Such contamination would manifest itself by the random occurrence of bacterial genes from the whole genome including many housekeeping genes. In this case, the genes occur as members of groups with specialised functions, suggesting that multiple horizontal gene transfers between bacteria and the coral genome have occurred [119]. Their precise function in *A. digitifera* remains unknown; homologues of these prokaryotic genes have not been described previously in any other eukaryote genome.

Linked closely with flagellar/ciliary proteins are the sensory receptors that signal chemoattraction or avoidance to direct cellular motility. The coral genome reveals a variety of genes that encode chemoreceptor and chemotaxis proteins (Table 4). The chemoreceptor proteins of *A. digitifera* include an oxygen-sensing aerotaxis receptor that in bacteria invokes an avoidance response to anoxic micro-environments [120]. Encoded also are a nematode sensory chemoreceptor homologue [121], two homologous pheromone factor receptor proteins that in fungi activate a species-specific mating response [122], three chemotaxis protein sensor receptors belonging to the methyl-accepting chemotaxis family of proteins (MCPs) in bacteria and archaea [123], and two proteins (CheZ and CheR) and two regulators (PixG and WspE) of the two-component signal transduction (TCST) system for activation of gene expression. In bacteria and archea, as well as some plants, fungi and protozoa [124], TCST systems mediate many cellular processes that respond to a broad range of environmental stimuli via activation of a specific histidine (or serine) kinase sensor and its cognate response regulator [125]. There are 77 sequence matches to various elements of the TCST family of proteins in the *A digitifera* genome (data not tabulated). Included also are genes encoding members of the chemotactic cytokine (chemokine) family of sensory proteins that on secretion directs chemotaxis in nearby responsive cells by stimulating target chemokine receptors; both chemokine and chemokine receptor proteins are encoded in the coral genome. Significantly, sensory chemokines/chemokine receptors are found in all vertebrates, some viruses and some groups of bacteria, but none have been described previously for invertebrates [126].

### Neural messengers, receptors and sensory proteins

Corals and other cnidarians are the earliest extant group of organisms to have a primitive nervous system network [127] thought to be evolved from a eumetazoan ancestor prior to the divergence of Cnidaria and the Bilateria [128,129]. Unlike marine sponges (Porifera) that predate synaptic innovation [130], cnidarians possess a homogenous nerve net that, although lacking any form of cephalization, accommodates fundamental neurosensory transmission across the nerve net to end in a motoneural junction to coordinate tentacle movement required for feeding and predator avoidance [131]. The nervous systems of cnidarians consist of both ectodermal sensory cells and their effector cells and endodermal multipolar ganglions capable of neurotransmission [132]. At the functional level, synaptic transmission in cnidarians relies on fast neurotransmitters (glutamate, GABA, glycine) and slow neurotransmitters (catecholamine, serotonin, neuropeptides) for sensory-signal conduction [133]. At the ultrastructural level, many cnidarian neurons have multifunctional traits of sensory, neurosecretory and stimulatory attributes [134]. Significantly, the genome of *A. digitifera* encodes the expression of a ciliary neurotrophic factor, which is a polypeptide hormone and nerve growth factor that promotes neurotransmitter synthesis, neurite outgrowth and regeneration [135]. Additionally, the coral genome encodes nerve growth factor and neurotrophic kinase receptors, a survival motor neuron protein, a survival neuron splicing factor, the neural outgrowth protein neurotrimin, and a neurotrophin growth factor attributed to signalling neuron survival, differentiation and growth (Table 5). Encoded for neuron regulation and development are several neuron cation-gated channels, a neuronal guanine nucleotide exchange factor, a neurotransmitter Na^+^ symporter, several neurogenic differentiation proteins, a neuronal PAS domain transcription factor for activation of neurogenesis, the axon guidance protein neurophilin-2, a neural crest protein of embryonic neural development, neural ELAV-like transcription proteins of neurogenesis, a Notch protein (79 sequence domain matches) and a neutralized protein subset of the Notch signalling pathway that promotes neuron proliferation in early neurogenic development. Structural elements of the coral nerve net include neurofilament polypeptides and neuronal adhesion proteins.

**Table 5:** **Neuronal and sensory proteins in the predicted proteome of *A. digitifera***

		
**Gene sequence**	**KEGG Orthology**	**Encoded protein description**
v1.01918 [+ 5 other sequence copies]	K01049	Acetylcholinesterase
v1.18087; v1.14516	K04136	Adrenergic receptor alpha-1B
v1.06394	K04137	Adrenergic receptor alpha-1D
v1.09628; v1.15688; v1.00966	K04140	Adrenergic receptor alpha-2C
v1.19831; v1.20450	K04142	Adrenergic receptor beta-2
v.17293	K00910	beta-Adrenergic-receptor kinase
v1.13740 [+ 5 other sequence copies]	K04828	Amiloride-sensitive cation channel 1, neuronal (degenerin)
v1.23541 [+ 6 other sequence copies]	K04829	Amiloride-sensitive cation channel 2, neuronal
v1.09323 [+ 4 other sequence copies]	K04439	beta-Arrestin
v1.07723; v1.22465	K04641	Bacteriorhodopsin
v1.08062	K05420	Ciliary neurotrophic factor
v1.03288 [+ 5 other sequence copies]	K02295	Cryptochrome
v1.20011; v1.20036; v1.20084; v1.18607	K04948	Cyclic nucleotide gated channel alpha 1
v1.21470	K04951	Cyclic nucleotide gated channel alpha 4
v1.21783; v1.01466; v1.01466; v1.01466	K05326	Cyclic nucleotide gated channel, invertebrate
v1.03645	K05391	Cyclic nucleotide gated channel, other eukaryote
v1.21256	K08762	Diazepam-binding inhibitor (GABA receptor, acyl-CoA-binding protein)
v1.22156 [+ 6 other sequence copies]	K00503	Dopamine beta-monooxygenase
v1.21775: v1.15989	K04148	Dopamine D1-like receptor
v1.14160; v1.01697	K04144	Dopamine receptor D1
v1.05089; v1.20018	K04145	Dopamine receptor D2
v1.14030; v1.23273	K04146	Dopamine receptor D3
v1.20536	K13088	ELAV-like protein 1
v1.18658 [+ 5 other sequence copies]	K13208	ELAV-like protein 2/3/4
v1.05774 [+ 18 other sequence copies]	K04313	G protein-coupled receptor 6
v1.00572; v1.18152	K08404	G protein-coupled receptor 17
v1.23842	K04316	G protein-coupled receptor 19
v1.03948	K08411	G protein-coupled receptor 26
v1.09271	K08383	G protein-coupled receptor 34
v1.05595	K04243	G protein-coupled receptor 37 (endothelin receptor type B-like)
v1.04019	K08409	G protein-coupled receptor 45
v1.19913; v1.09821; v1.04291	K08450	G protein-coupled receptor 56
v1.05404	K04321	G protein-coupled receptor 63
v1.02179; v1.10397	K08451	G protein-coupled receptor 64
v1.23269 [+ 5 other sequence copies]	K08408	G protein-coupled receptor 68
v1.21091	K08421	G protein-coupled receptor 84
v1.11008	K04302	G protein-coupled receptor 85
v1.21884; v1.01951	K08452	G protein-coupled receptor 97
v1.03243 [+ 13 other sequence copies]	K08378	G protein-coupled receptor 103
v1.13790; v1.18939	K08453	G protein-coupled receptor 110
v1.09442; v1.14019	K08455	G protein-coupled receptor 112
v1.24009	K08456	G protein-coupled receptor 113
v1.04290	K08459	G protein-coupled receptor 114
v1.06608; v1.24223	K08457	G protein-coupled receptor 115
v1.10800 [+ 6 other sequence copies]	K08458	G protein-coupled receptor 116
v1.07662 [+ 6 other sequence copies]	K08462	G protein-coupled receptor 125
v1.09663; v1.08981	K08463	G protein-coupled receptor 126
v1.24252	K08464	G protein-coupled receptor 128
v1.02750 [+ 26 other sequence copies]	K08465	G protein-coupled receptor 133
v1.05774 [+ 11 other sequence copies]	K08466	G protein-coupled receptor 144
v1.05497; v1.13272; v1.01323	K08436	G protein-coupled receptor 152
v1.08653 [+ 5 other sequence copies]	K08467	G protein-coupled receptor 157
v1.11807; v1.10392; v1.10394	K08469	G protein-coupled receptor 158
v1.07294; v1.00247	K08439	G protein-coupled receptor 161
v1.05167	K08442	G protein-coupled receptor 176
v1.08677; v1.23465; v1.19865; v1.06986	K12762	G protein-coupled receptor GPR1
v1.13395	K08291	G protein-coupled receptor kinase
v1.18529; v1.07599; v1.05558	K12487	G protein-coupled receptor kinase interactor 2
v1.02481	K04619	G protein-coupled receptor family C group 5 member B
v1.22242	K04622	G protein-coupled receptor family C group 6 member A
v1.08625; v1.13650; v1.13048; v1.18694	K04599	G protein-coupled receptor Mth (Methuselah protein)
v1.07465; v1.10540	K08341	GABA(A) receptor-associated protein (autophagy-related protein 8)
v1.09831 [+ 30 other sequence copies]	K05270	Gamma-aminobutyric acid (GABA) receptor, invertebrate
v1.18702; v1.11701	K05183	Gamma-aminobutyric acid (GABA) A receptor beta-3
v1.04252 [+ 6 other sequence copies]	K05185	Gamma-aminobutyric acid (GABA) A receptor epsilon
v1.06325	K05186	Gamma-aminobutyric acid (GABA) A receptor gamma-1
v1.00048	K05188	Gamma-aminobutyric acid (GABA) A receptor gamma-3
v1.07506 [+ 6 other sequence copies]	K04615	Gamma-aminobutyric acid (GABA) B receptor 1
v1.07506 [+ 24 other sequence copies]	K04616	Gamma-aminobutyric acid (GABA) B receptor 2
v1.06426; v1.10563; v1.01138	K05192	Gamma-aminobutyric acid (GABA) receptor theta
v1.15485	K05198	Glutamate receptor, ionotropic, AMPA 2
v1.09807	K05200	Glutamate receptor, ionotropic, AMPA 4
v1.04764	K05207	Glutamate receptor, ionotropic, delta 2
v1.15247 [+ 12 other sequence copies]	K05313	Glutamate receptor, ionotropic, invertebrate
v1.15247 [+ 7 other sequence copies]	K05202	Glutamate receptor, ionotropic, kainate 2
v1.00617	K05203	Glutamate receptor, ionotropic, kainate 3
v1.09688 [+ 6 other sequence copies]	K05208	Glutamate receptor, ionotropic, N-methyl D-aspartate 1
v1.21204 [+ 4 other sequence copies]	K05212	Glutamate receptor, ionotropic, N-methyl-D-aspartate 2D
v1.01622	K05214	Glutamate receptor, ionotropic, N-methyl-D-aspartate 3B
v1.01418 [+ 5 other sequence copies]	K05387	Glutamate receptor, ionotropic, other eukaryote
v1.04275	K05194	Glycine receptor alpha-2
v1.10737; v1.06885	K05195	Glycine receptor alpha-3
v1.05488	K05271	Glycine receptor alpha-4
v1.08900; v1.06885	K05196	Glycine receptor beta
v1.18634	K05397	Glycine receptor, invertebrate
v1.14569; v1.14570	K09071	Heart-and neural crest derivatives-expressed protein
v1.16783 [+ 4 other sequence copies]	K02168	High-affinity choline transport protein
v1.13837	K07608	Internexin neuronal intermediate filament protein, alpha
v1.01671	K04309	Leucine-rich repeat-containing G protein-coupled receptor 4
v1.09480; v1.05605	K04308	Leucine-rich repeat-containing G protein-coupled receptor 5
v1.15300 [+ 8 other sequence copies]	K08399	Leucine-rich repeat-containing G protein-coupled receptor 6
v1.17524 [+ 14 other sequence copies]	K04306	Leucine-rich repeat-containing G protein-coupled receptor 7
v1.21700; v1.03578; v1.17196	K04307	Leucine-rich repeat-containing G protein-coupled receptor 8
v1.16104	K08396	Mas-related G protein-coupled receptor member X
v1.08718; v1.02042; v1.02042	K04604	Metabotropic glutamate receptor 1/5
v1.22794 [+ 7 other sequence copies]	K04605	Metabotropic glutamate receptor 2/3
v1.15331	K04607	Metabotropic glutamate receptor 4
v1.01418	K04608	Metabotropic glutamate receptor 6/7/8
v1.21698; v1.04544; v1.21739	K14636	MFS transporter, solute carrier family 18 (acetylcholine transporter) 3
v1.05751; v1.19720; v1.22165; v1.02336	K04134	Muscarinic acetylcholine receptor
v1.11550	K04129	Muscarinic acetylcholine receptor M1
v1.01913 [+ 4 other sequence copies]	K04131	Muscarinic acetylcholine receptor M3
v1.18723	K04132	Muscarinic acetylcholine receptor M4
v1.08171	K04133	Muscarinic acetylcholine receptor M5
v1.07408 [+ 34 other sequence copies]	K02583	Nerve growth factor receptor (TNFR superfamily member 16)
v1.15265 [+ 91 other sequence copies]	K06491	Neural cell adhesion molecule
v1.13789; v1.24010; v1.03980	K09038	Neural retina-specific leucine zipper protein
v1.24586; v1.16386; v1.16387	K08052	Neurofibromin 1
v1.05520; v1.15407; v1.07950	K04572	Neurofilament light polypeptide
v1.19724	K04573	Neurofilament medium polypeptide (neurofilament 3)
v1.15787 [+ 4 other sequence copies]	K09081	Neurogenin 1 (neurogenic differentiation protein)
v1.00345; v1.05338; v1.10997	K08033	Neurogenic differentiation factor 1
v1.07355; v1.14517	K09078	Neurogenic differentiation factor 2
v1.08832	K09079	Neurogenic differentiation factor 4
v1.06678; v1.06677	K01393	Neurolysin
v1.16238 [+ 19 other sequence copies]	K06756	Neuronal cell adhesion molecule
v1.20460; v1.16967	K06757	Neurofascin NFASC (cell adhesion molecule CAMs)
v1.22060; v1.03561	K07525	Neuronal guanine nucleotide exchange factor
v1.03908	K09098	Neuronal PAS domain-containing protein 1/3
v1.00089	K05247	Neuropeptide FF-amide peptide
v1.21565	K08375	Neuropeptide FF receptor 2
v1.06392 [+ 11 other sequence copies]	K04209	Neuropeptide Y receptor, invertebrate
v1.08609 [+ 31 other sequence copies]	K06819	Neuropilin 2
v1.11492 [+ 5 other sequence copies]	K03308	Neurotransmitter:Na+ symporter, NSS family
v1.16744 [+ 8 other sequence copies]	K06774	Neurotrimin
v1.05353	K03176	Neurotrophic tyrosine kinase receptor type 1
v1.20055	K04360	Neurotrophic tyrosine kinase receptor type 2
v1.03803	K04356	Neurotrophin 3
v1.09523	K04803	Nicotinic acetylcholine receptor alpha-1 (muscle)
v1.11940	K04806	Nicotinic acetylcholine receptor alpha-4
v1.01548	K04808	Nicotinic acetylcholine receptor alpha-6
v1.05056; v1.12097	K04809	Nicotinic acetylcholine receptor alpha-7
v1.07222; v1.11069	K04810	Nicotinic acetylcholine receptor alpha-9
v1.18231 [+ 32 other sequence copies]	K05312	Nicotinic acetylcholine receptor, invertebrate
v1.24404	K04813	Nicotinic acetylcholine receptor beta-2 (neuronal)
v1.06514; v1.23640	K04815	Nicotinic acetylcholine receptor beta-4
v1.18634	K04816	Nicotinic acetylcholine receptor delta
v1.18231 [+ 32 other sequence copies]	K05312	Nicotinic acetylcholine receptor, invertebrate
v1.05293 [+ 78 other sequence copies]	K02599	Notch protein
v1.15348 [+ 4 other sequence copies]	K04256	c-Opsin protein
v1.01972	K08385	G0-Opsin protein
v1.13345 [+ 5 other sequence copies]	K04255	r-Opsin protein
v1.00749; v1.03435	K00504	Peptidylglycine monooxygenase
v1.12323 [+ 11 other sequence copies]	K00678	Phosphatidylcholine-retinol O-acyltransferase
v1.18340 [+ 6 other sequence copies]	K09624	Protease, serine, 12 (neurotrypsin, motopsin)
v1.08030 [+ 9 other sequence copies]	K01931	Protein neuralized
v1.04431	K09333	Retina and anterior neural fold homeobox-like protein
v1.01789; v1.06542	K00061	Retinol dehydrogenase
v1.05804 [+ 6 other sequence copies]	K11150	Retinol dehydrogenase 8
v1.22340; v1.14029	K11151	Retinol dehydrogenase 10
v1.24399; v1.07017	K11154	Retinol dehydrogenase 16
v1.19667; v1.16885; v1.24371	K00909	Rhodopsin kinase
v1.12432; v1.15302; v1.07505	K09516	all-*trans*-Retinol 13,14-reductase
v1.09104 [+ 6 other sequence copies]	K05613	Solute carrier family 1 (glial high affinity glutamate transporter), member 2
v1.19779; v1.08769; v1.22032	K05617	Solute carrier family 1 (high affinity Asp/glutamate transporter), member 6
v1.19293; v1.19292	K14387	Solute carrier family 5 (high affinity choline transporter), member 7
v1.10901; v1.19493	K05336	Solute carrier family 6 (neurotransmitter transporter), invertebrate
v1.24615 [+ 10 other sequence copies]	K05034	Solute carrier family 6 (neurotransmitter transporter, GABA) member 1
v1.07932	K05046	Solute carrier family 6 (neurotransmitter transporter, GABA) member 13
v1.01817	K05036	Solute carrier family 6 (neurotransmitter transporter, dopamine) member 3
v1.20691; v1.16333; v1.15484; v1.02123	K05038	Solute carrier family 6 (neurotransmitter transporter, glycine) member 5
v1.15484; v1.15484	K05042	Solute carrier family 6 (neurotransmitter transporter, glycine) member 9
v1.18461; v1.09068; v1.02237; v1.20880	K05333	Solute carrier family 6 (neurotransmitter transporter) member 18
v1.02239; v1.13836; v1.09067	K05334	Solute carrier family 6 (neurotransmitter transporter) member19
v1.21997 [+ 5 other sequence copies]	K12839	Survival of motor neuron-related-splicing factor member 30
v1.21997 [+ 6 other sequence copies]	K13129	Survival motor neuron protein

Cnidarians differentiate highly specialised sensory and mechanoreceptor cells involved in the capture of prey and for defence against predators. Their stinging cells, termed nematocysts or cnidocytes, are stimulated by adjacent chemosensory cells. Nematocysts trigger the release of a stinging barb (cnidae tubule) via ultra-fast exocytosis on physical contact with ciliary mechanoreceptors of the cnidocyte to deliver the discharge of its venom [136]. Despite considerable advances in the sensory biology of cnidarians, knowledge of the specific receptor genes that regulate cnidocyte function remains incomplete. In *Hydra*, and perhaps other cnidarians, cnidocyte discharge is controlled by an ancient light-activated, opsin-mediated phototransduction pathway [137] that precedes the evolution of cubozoan (box jellyfish) eyes [138]; cubozoans are the most basal of animals to have eyes containing a lens and ciliary-type visual cells similar to that of vertebrate eyes [139]. These G-coupled opsin photoreceptors of the retinylidene-forming protein family encoded in the genome of *A. digitifera* include rhodopsin, bacteriorhodopsin, c-opsin, r-opsin and G_0_-opsin (Table 5), but not the Gs-subfamily of opsin receptors reported to be present in sea anemones, hydra and jellyfish [140], that together with cyclic nucleotide-gated (CNG) ion channel proteins, arrestin (β-adrenergic receptor inhibitor) and other retino-protein receptors, are usual components of the bilaterian phototransduction cascade. Present also are genes to express rhodopsin kinase and β-adrenergic receptor kinase which are related members of the serine/threonine kinase family of proteins that specifically initiate deactivation of G-protein coupled receptors. Additional proteins of retinol metabolism of the phototransduction pathway encoded in the *A. digitifera* genome are retinol dehydrogenase, all-*trans*-retinol 13,14 reductase and phosphatidylcholine (lichthin)-retinol O-acyltransferase, a neural retina-specific leucine zipper protein that is an intrinsic regulator of photoreceptor development and function, and a retina and anterior neural fold homeobox-like protein that modulates the expression of photoreceptor genes within the rhodopsin promoter. The genome of *A. digitifera* encodes also a blue light-sensing, cryptochrome photoreceptor thought to signal synchronous coral spawning by detecting illumination from the lunar cycle [98,99].

The *A. digitifera* genome reveals genes to express a broad array of neurotransmitter receptor proteins (Table 5), including glycine and glutamate neuroreceptors, adrenergic receptors that target non-dopamine catecholamines (i.e., epinephrine and norepinephrine), dopamine, muscarinic and nicotinic acetylcholine receptors, sensory G protein-coupled receptors and γ-aminobutyric acid (GABA) ligand-gated ion channel and G protein-coupled receptors (and inhibitors), several of which are encoded in high copy numbers. Cellular trafficking of neurotransmitters to presynaptic terminals is essential for neurotransmission, and significantly the genome of *A. digitifera* encodes a wide range of solute carrier neurotransmitter transporters, including a high affinity choline transporter and an acetylcholine-specific protein belonging to the major facilitator superfamily (MFS) of secondary transporters. Encoded also is dopamine β-monooxygenase that catalyses the conversion of dopamine to norepinephrine in the catecholamine biosynthetic pathway, which is necessary for cross-activation of adrenergic neuroreceptors [141]. Notably, the *A. digitifera* genome encodes acetylcholinesterase that is expressed at neuromuscular junctions and cholinergic synapses where its protease activity serves to terminate synaptic transmission.

The primitive nervous networks of cnidarians are strongly peptidergic with at least 35 neuropeptides identified from different cnidarian classes [142]. Our annotation of the sequenced *A. digitifera* genome, however, revealed only the neuropeptide FF-amide neurotransmitter, a RF amide related peptide, and its neuropeptide FF and Y receptors (Table 5). Neuropeptides are usually expressed as large precursor proteins which comprise multiple copies of “immature” neuropeptides. Our annotation did not readily reveal these precursor neuropeptide proteins, but we did find enzymes required for their processing, for example, a variety of carboxypeptidase enzymes (not tabulated) that remove propeptide carboxyl residues at basic peptidase sites, and the mature peptide neurotransmitters that are finished by consecutive modification by peptidylglycine (α-hydroxylating) monooxidase (PHM) and peptidyl α-hydroxyglycine α-amidating lyase (PAL) enzymes, both of which are commonly expressed in mammals as a single bifunctional peptidylglycine monooxygenase (K00504/EC 1.14.17.3) [143]. Our extensive catalogue of animal-like neural and sensory proteins revealed by genome annotation is testament that essential neurobiological features were developed in the primitive neural networks of early eumetazoan evolution.

### Calcification and Ca^2+^-signalling proteins

The massive structures of coral reefs evident today are a construction of aggregated calcium carbonate deposited over long geological time by scleractinian corals and other calcifying organisms, yet our understanding of the molecular processes that regulate the biological processes of coral calcification is limited [144]. Ca^2+^ transfer from seawater to the calicoblastic site of coral calcification occurs by passive diffusion through the gastrovascular cavity [145] and by active calcium transport [146]. Active entry of Ca^2+^ through the oral epithelial layer is regulated by voltage-dependent calcium channels, such as demonstrated by the L-type alpha protein cloned from the reef-building coral *Stylophora pistillata*[147]. Ca^2+^ transport across the calioblastic ectoderm to the extracellular calcifying site is facilitated by the plasma-membrane ATP-dependent calcium pump that in *S. pistillata* resemble the Ca^2+^-ATPase family of mammalian proteins [148]. By 2H^+^/Ca^2+^-exchange at the calioblastic membrane, Ca^2+^-ATPase removes H^+^ (from the net reaction Ca^2+^ + CO_2_ + H_2_O ⇒ CaCO_3_ + 2H^+^) thereby increasing the saturation state of CaCO_3_ to sustain calcium precipitation [146]. Importantly, located also at the calicoblastic membrane is carbonic anhydrase [149] which is required to catalyse the intermediate step of calcification by the reversible hydration of carbon dioxide (CO_2_ + H_2_O ⇒ HCO_3_^-^ + H^+^). In coral phototrophic symbiosis, despite numerous studies describing the well-known phenomenon of light-enhanced calcification, the relationship linking symbiont photosynthesis to coral calcification has been elusive [150,151]. Nonetheless, efforts to better understand the calcifying response of scleractinian corals to environmental change and ocean acidification are gaining traction [149,152,153].

Voltage-gated calcium channels (VGCCs) have been examined extensively in mammalian physiology for converting membrane potential into intracellular Ca^2+^ transients for signalling transduction pathways (reviewed in [154]). VGCC signalling affects cellular processes to include muscle contraction, neuronal excitation, gene transcription, fertilisation, cell differentiation and development, proliferation, hormone release, activation of calcium-dependent protein kinases, cell death via necrosis and apoptosis pathways, phagocytosis and endo/exocytosis. Remarkably, annotation of the genome of *A. digitifera* reveals sequences encoding homologues of all the VGCC (α, αδ, β, and γ) subunits of the molecular (L, N, P/Q and R) phenotypes expressed in mammalian physiology (Table 6). There are multiple sequences encoding three variants of Ca^2+^-transporting ATPase, of which at least one is necessary for coral calcification. There is only one sequence match for expressing carbonic anhydrase in the genome of *A. digitifera*, which may reflect the high catalytic efficiency of this calcifying enzyme [155], although a BLAST search of ZoophyteBase does reveal scaffolds with low e-values which on future experimental inspection might uncover multiple copies of this enzyme essential for calcification. There are multiple sequences that express solute carrier Na^+^/Ca^2+^- and Na^+^/K^+^/Ca^2+^-exchange families of transport proteins that with expression of the coral Ca^2+^/H^+^-antiporter may regulate cellular pH and Ca^2+^ homeostasis.

**Table 6:** **Calcification and Ca**^**2+**^**-signalling proteins in the predicted proteome of *A. digitifera***

		
**Gene sequence**	**KEGG Orthology**	**Encoded protein description**
v1.06452; v1.06451; v1.24424; v1.16923	K07300	Ca2+:H+ antiporter
v1.01669 [+ 9 other sequence copies]	K01537	Ca2+−transporting ATPase
v1.22367; v1.22366; v1.22365	K05850	Ca2+ transporting ATPase, plasma membrane
v1.19074	K05853	Ca2+ transporting ATPase, sarcoplasmic/endoplasmic reticulum
v1.22416; v1.22417; v1.15682; v1.00750	K14757	Calbindin D28
v1.24568 [+ 9 other sequence copies]	K01672	Carbonic anhydrase
v1.09241	K08272	Calcium binding protein 39
v1.02323 [+ 39 other sequence copies]	K13448	Calcium-binding protein CML
v1.05162 [+ 21 other sequence copies]	K13412	Calcium-dependent protein kinase
v1.09352	K07359	Calcium/calmodulin-dependent protein kinase kinase
v1.06475; v1.07555;v1.00945; v1.00159; v1.21122	K08794	Calcium/calmodulin-dependent protein kinase I
v1.06475; v1.01061; v1.21150; v1.22443	K04515	Calcium/calmodulin-dependent protein kinase II
v1.00159	K05869	Calcium/calmodulin-dependent protein kinase IV
v1.21927; v1.01218; v1.22226; v1.06623; v1.13703	K06103	Calcium/calmodulin-dependent serine protein kinase
v1.13460	K08284	Calcium channel MID1
v1.20738; v1.01401	K12841	Calcium homeostasis endoplasmic reticulum protein
v1.22794 [+ 11 other sequence copies]	K04612	Calcium-sensing receptor
v1.10079 [+ 17 other sequence copies]	K02183	Calmodulin
v1.10994	K14734	S100 calcium binding protein G
v1.02488 [+ 14 other sequence copies]	K05849	Solute carrier family 8 (sodium/calcium exchanger)
v1.23153 [+ 9 other sequence copies]	K13749	Solute carrier family 24 (sodium/potassium/calcium exchanger)
v1.14863	K12304	Soluble calcium-activated nucleotidase 1
v1.18656 [+ 13 other sequence copies]	K04858	Voltage-dependent calcium channel alpha-2/delta-1
v1.13222	K04860	Voltage-dependent calcium channel alpha-2/delta-3
v1.08078 [+ 9 other sequence copies]	K05315	Voltage-dependent calcium channel alpha 1, invertebrate
v1.03896 [+ 6 other sequence copies]	K05316	Voltage-dependent calcium channel alpha-2/delta, invertebrate
v1.04798	K05317	Voltage-dependent calcium channel beta, invertebrate
v1.22788	K04863	Voltage-dependent calcium channel beta-2
v1.09999	K04872	Voltage-dependent calcium channel gamma-7
v1.02505	K04873	Voltage-dependent calcium channel gamma-8
v1.03648[+ 6 other sequence copies]	K04850	Voltage-dependent calcium channel L type alpha-1C
v1.03648; v1.17267	K04851	Voltage-dependent calcium channel L type alpha-1D
v1.03648; v1.13219; v1.21895	K04857	Voltage-dependent calcium channel L type alpha-1S
v1.06313; v1.01656; v1.23096	K04344	Voltage-dependent calcium channel P/Q type alpha-1A
v1.08078 [+ 10 other sequence copies]	K04849	Voltage-dependent calcium channel N type alpha-1B
v1.07968	K04852	Voltage-dependent calcium channel R type alpha-1E
v1.01364; v1.13467; v1.08705	K04854	Voltage-dependent calcium channel T type alpha-1G
v1.15414; v1.14241; v1.09595	K04855	Voltage-dependent calcium channel T type alpha-1H

Implicit to coral calcification is Ca^2+^ regulation that affects signalling of other vital cellular functions. Cellular Ca^2+^ is mediated by the calcium-sensing receptor calmodulin (18 sequence matches) and other messenger calcium-binding effectors (Table 6), including the calcium-binding protein CML (40 protein domain sequence matches). Calcium/calmodulin-protein kinase proteins are arguably key to Ca^2+^-signalling in coral symbiosis but, with the exception of activation of sperm flagellar motility [156], their precise role has not been elaborated.

### Plant-derived proteins

Endosymbiosis has contributed greatly to eukaryotic evolution, most notably to the genesis of plastids and mitochondria derived from prokaryotic antecedents. Genetic integration by endosymbiont-to-host transfer (EGT) or replacement (EGR) has been a significant force in early metazoan innovation, whereby nuclear transferred genes may even adopt novel functions in the host cell or replace existing versions of the protein that they encode [157]. Prokaryote-to-eukaryotic gene transfer has been widespread in evolution, but examples of genetic exchange between unrelated eukaryotes, such as between algal symbionts and their multicellular eukaryote host, are considered rare (reviewed by [158,159]). One such example is *aroB* (3-dehydroquinate synthase) transferred to the genome of the sea anemone *N. vectensis*, which sequence best fits that of the dinoflagellate *Oxyrrhis marina*[119]. Close inspection of the amino acid sequence of the *aroB* gene product, as reported by Shinzato et al. [45], clearly shows this protein to be 2-*epi*-5-*epi*-valiolone synthase (EVS), a sugar phosphate cyclase orthologue that catalyses the conversion of sedoheptulose 7-phosphate to 2-*epi*-5-*epi*-valiolone found to be a precursor of the mycosporine-like amino acid (MAA) sunscreen shinorine in the cyanobacterium *Anabaena variabilis*[160]. Additionally, the EVS gene of *N. vectensis* has a distinctive *O*-methytransferase fusion that is identical in *O. marina*[161]. The shikimate pathway is essential to apicomplexan parasites of the genera *Plasmodium*, *Toxoplasma* and *Cryptosporidium* and of *Tetrahymena* ciliates to express a pentafunctional *aroM* gene similar to that of Ascomycetes, which is thought to have been conveyed by fungal gene transfer to a common ancestral progenitor [162]. In a separate example, *H. viridis* expresses a plant-like ascorbate peroxidase gene (*HvAPX1*) during oogenesis in both symbiotic and aposymbiotic individuals [163], whereby peroxidase activity is coincident with oogenesis and embryogenesis that in *Hydra* acts as a ROS scavenger to protect the oocyte from apoptotic degradation [164]. The sacoglossan (sea slug) molluscs *Elysia chlorotica* and *E. viridis* (Plakobranchidae) acquire plastids on ingestion of the siphonaceous alga *Voucherea litorea* (termed “kleptoplasty”) and, by maintaining sequestered plastids in an active photosynthetic state, has emerged as a model organism for the transfer of nuclear-encoded plant genes from algal symbiont to its animal host [165]. In this symbiosis, the family of light-harvesting genes *psbO*, *prk* (phosphoribokinase) and chlorophyll synthase (*chlG*) are entrained in the genome of *Elysia chlorotica* (reviewed in [166,167]), although there is debate whether these genes are transcriptionally expressed (compare [168] and [169]). Also, phylogenomic analysis of the predicted proteins of the aposymbiotic unicellular choano-flagellate *Monosiga brevicollis*, considered to be a stem progenitor of the animal kingdom [170,171], reveals 103 genes having strong algal affiliations arising from multiple phototrophic donors [172]. Such notable examples illustrate the transfer of algal genes to animal recipients.

KEGG orthology-based annotation of the predicted proteome of *A. digitifera* reveals a plethora of sequences presumed to be of algal origin (Table 7). Like *E. chlorotica*, the coral genome has encoded the photosystem II (PSII) protein PsbO of the oxygen-evolving complex of photosynthesis, as well as the PSII light-harvesting complex protein PsbL that is important in protecting PSII from photo-inactivation [173]. Encoded also are the photosystem I subunit proteins PsaI and PsaO. Additionally encoded are the photosystem P840 reaction center cytochrome c551 (PscC) protein and the photosynthetic reaction center M subunit protein, the light-harvesting proteins complex 1 alpha (PufA), the complex II chlorophyll *a*/*b* binding protein 6 (LHCB6), the cyanobacterial phycobilisome proteins AcpF and AcpG, the phycocyanin-associated antenna protein CpcD, the phycocyanobilin lyase protein CpcF and the phycoerythrin-associated linker protein CpeS. Like *E. chlorotica*, the coral genome encodes chlorophyll synthase (ChlG), a chlorophyll transporter protein PucC, a light-independent nitrogenase-like protochlorophyllide reductase enzyme that is sensitive to oxygen [174] and a red chlorophyll reductase essential to the detoxification of photodynamic chlorophyll catabolites arising from plant/algal senescence [175]. Three chlorosome proteins of the photosynthetic antenna complex of green sulphur bacteria, a bacteriochlorophyll methyltransferase involved in BChl *c* biosynthesis [176] and the retinylidene bacteriorhodopsin of phototrophic Archaea are also encoded in the coral genome. Present are genes encoding subunit 6 of the cytochrome B_6_f complex that links PSII and PSI via the plastoquinone pool, together with chloroplast ferredoxin-like NapH and NapG proteins and their 2Fe-2S cluster protein. The coral genome, however, encodes sequences for NAD^+^-ferredoxin reductase (HcaD; not tablulated), rather than the required NADP^+^-ferredoxin reductase of photosynthesis. Annotation of the *A. digitifera* genome revealed genes unexpectedly encoding ferredoxin hydrogenase [EC:1.12.7.2] and that of its small subunit protein (Table 7) involved in light-dependent production of molecular hydrogen having its [Fe-Fe]-cluster coupled to the photosynthetic transport chain via a charge-transfer complex with ferredoxin (see [177]).

**Table 7:** **Plant-derived proteins in the predicted proteome of *A. digitifera***

		
**Gene sequence**	**KEGG Orthology**	**Encoded protein description**
v1.14452	K09843	(+)-Abscisic acid 8′-hydroxylase
v1.18868	K14496	Abscisic acid receptor PYR/PYL family (PYL)
v1.21983; v1.05890	K03342	p-Aminobenzoate synthetase / 4-amino-4-deoxychorismate lyase (PabBC)
v1.15436	K02822	Ascorbate-specific IIB component, PTS system (PTS-Ula-EiiB)
v1.11187; v1.13966	K00423	L-Ascorbate oxidase
v1.20081; v1.22465	K13604	Bacteriochlorophyll C20 methyltransferase (BchU)
v1.07723	K04641	Bacteriorhodopsin (BoP)
v1.21858	K04040	Chlorophyll synthase (ChlG)
v1.01742	K08945	Chlorosome envelope protein A (CsmA)
v1.04797; v1.14208	K08946	Chlorosome envelope protein B (CsmB)
v1.18698	K08948	Chlorosome envelope protein D (CamD)
v1.18637	K02642	Cytochrome b_6_f complex subunit 6 (PetL)
v1.21101; v1.14192; v1.14548	K01735	3-Dehydroquinate synthase (AroB)
v1.05796	K10210	4,4′-Diaponeurosporene oxidase (carotenoid biosynthesis; CrtP)
v1.11730	K04755	Ferredoxin, 2Fe-2S (FdX)
v1.19154; v1.00014	K00532	Ferredoxin hydrogenase
v1.00014	K00534	Ferredoxin hydrogenase small subunit
v1.17698; v1.06031; v1.16647	K02574	Ferredoxin-type protein (NapH)
v1.23058	K02573	Ferredoxin-type protein (NapG)
v1.08414	K08926	Light-harvesting complex 1 alpha chain (PufA)
v1.21458	K08917	Light-harvesting complex II chlorophyll a/b binding protein 6 (LHCB6)
v1.03743	K08226	MFS transporter, BCD family, chlorophyll transporter (PucC)
v1.13030; v1.08678	K13413	Mitogen-activated protein kinase kinase 4/5, plant ((MKK4_5P)
v1.02429; v1.10744; v1.03340	K08929	Photosynthetic reaction center M subunit (PufM)
v1.03631	K02696	Photosystem I subunit VIII (PsaI)
v1.11432	K14332	Photosystem I subunit (PsaO)
v1.17422	K02713	Photosystem II protein (PsbL)
v1.18303	K02716	Photosystem II oxygen-evolving enhancer protein 1 (PsbO)
v1.12300; v1.21136	K08942	Photosystem P840 reaction center cytochrome c551 ((PscC)
v1.00280	K02097	Phycobilisome core component 9 (AcpF)
v1.10967	K02290	Phycobilisome rod-core linker protein (AcpG)
v1.02166	K02287	Phycocyanin-associated, rod protein (CpcD)
v1.19642; v1.07305; v1.19572; v1.01248	K02289	Phycocyanobilin lyase beta subunit (CpcF)
v1.10441	K05382	Phycoerythrin-associated linker protein (CpeS)
v1.13406	K10027	Phytoene dehydrogenase (desaturase; CrtI)
v1.18809; v1.06199	K02291	Phytoene synthase (CrtB)
v1.20411; v1.02037; v1.14064; v1.21095	K09060	Plant G-box-binding factor (GBF)
v1.10035	K00218	Protochlorophyllide reductase [NifEN-like; Por]
v1.21846	K05358	Quinate dehydrogenase (QuiA)
v1.03127	K13545	Red chlorophyll catabolite reductase (ACD2)
v1.05899	K00891	Shikimate kinase (AroK, AroL)
v1.21101; v1.14192; v1.05899	K13829	Shikimate kinase / 3-dehydroquinate synthase (AroKB)
v1.12938	K08500	Syntaxin of plants (SYP6)
v1.06575	K08506	Syntaxin of plants (SYP7)
v1.04929	K09834	Tocopherol cyclase (VTE1, SXD1)
v1.01022	K05928	Tocopherol *O*-methyltransferase
v1.05457	K09838	Zeaxanthin epoxidase (ZEP, ABA1)

Like *N. vectensis* and the dinoflagellate *Oxyrrhis marina*, the genome *of A. digitifera* encodes an *O*-methyltransferase which is immediately downstream of EVS, but the two genes are not fused. Using a ZoophyteBase BlastP search, the *O*-methyltransferase showed little sequence homology with the corresponding protein of *A. variabilis* (e-value of 6.972E^-2^ and Bit score of 34.27), whereas the EVS protein shared 87% absolute sequence identity to the *A. variabilis* EVS protein. What role, if any, these two genes play in mycosporine-like amino acid (MAA) biosynthesis in *A. digitifera* has yet to be determined, although it has been suggested from the transcriptome of *Acropora microphthalma* that MAA biosynthesis proceeds from a branch point at 3-dehydroquinate of the shikimic acid pathway as a shared metabolic adaptation between the coral host and its symbiotic zooxanthellae [40]. The 3-dehydroquinate synthase enzyme of the shikimic acid pathway, thought to be a key intermediate in an alternative MAA biosynthetic pathway in *A. variabilis*[178], is instead encoded by the fused *aroKB* gene of *A. digitifera* (Table 7). Additional shikimate proteins of the predicted proteome, although not limited to phototrophs, are shikimate kinase (AroK), quinate dehydrogenase (QuiA) and the conjoined *p*-aminobenzoate synthase and 4-amino-4-deoxychlorismate lysate (PabBC) enzyme necessary for folate biosynthesis [179]. Other plant-related gene homologues include the phytohormone abscisic acid receptor protein (PabBC) and its cytochrome P450 monooxygenase abscisic acid 8′-hydroxylase, L-ascorbate oxidase and PTS system degrading enzymes, the unique SYP6 and SYP7 syntaxins of plant vesicular transport, tocopherol cyclase and a tocopherol *O*-methyltransferase enzyme that converts γ-tocopherol to α-tocopherol. Essential for carotene biosynthesis are phytoene synthase (CrtB) and phytoene dehydrogenase (CrtI) enzymes. Significantly, encoded within the coral genome is zeaxanthin epoxidase that is essential for abscisic acid biosynthesis and is a key enzyme in the xanthophyll cycle of plants and algae to impart oxidative stress tolerance.

Given that viruses often mediate gene transfer processes, it is intriguing that certain bacteriophages of marine *Synechococcus* and *Prochlorococcus* cyanobacteria are reported to carry genes encoding the photosynthesis D1 (*psbA*), and D2 (*psbD*) proteins, a high-light inducible protein (HLIP) [180,181] and the photosynthetic electron transport plastocyanin (*petE*) and ferredoxin (*petF*) proteins thought to enhance the photosynthetic fitness of their host [182-184]. Accordingly, it has been suggested that the transfer of *psbA* by viruses associated with *Symbiodinium* could lessen the severity of thermal impairment to PSII and the response of corals to thermal bleaching [185]. It is yet unknown if phages or dinoflagellate-infecting viruses [186], particularly those of *Symbiodinium*[187], may affect gene transfer leading to complementary (or “shared”) metabolic adaptations of symbiosis [119,188].

### Proteins of nitrogen metabolism

It is well accepted that intracellular *Symbiodinium* spp. provide reduced carbon for coral heterotrophic metabolism by photosynthetic carbon fixation. Because of this metabolic relationship, light is a critical feature in the bioenergetics of coral symbiosis [189]. The algal photosynthate translocated to corals, however, is deficient in nitrogen at levels necessary to sustain autotrophic growth. While corals can assimilate fixed nitrogen from surrounding seawater [190], “recycled” nitrogen within the symbiosis may account for as much as 90% of the photosynthetic nitrogen demand [191]. It would not be surprising then that light would have a strong influence on the uptake and retention of ammonium by symbiotic corals. Consequently, corals excrete excess ammonium in darkness [192], and in light excretion is induced by treatment with the photosynthetic electron transport inhibitor 3-(3,4-diclorophenyl)-1,1-dimethylurea (DCMU) [193]. Since ammonia is the product of nitrogen fixation, these observations suggest that the coral holobiont may fix nitrogen in the dark, or when photosynthesis is repressed, during which coral tissues are hypoxic [194], and nitrogenase activity is not inactivated by molecular oxygen [195].

Tropical coral reefs are typically surrounded by low-nutrient oceanic waters of low productivity but, paradoxically, the waters of coral reefs often have elevated levels of inorganic nitrogen [196,197] attributed to high rates of nitrogen fixation. While nitrogen fixation from diazotrophic epiphytes of the coral reef substrata and sediments [197,198] and diazotrophic bacterioplankton of the coral reef lagoon [199] provide substantial quantities of fixed nitrogen for assimilation by the coral reef, mass-balance estimates show this input to be less than the community’s annual nitrogen demand [200]. Endolithic nitrogen-fixingbacteria are abundant in the skeleton of living corals where they benefit from organic carbon excreted by overlaying coral tissues to provide a ready source of energy for dinitrogen reduction [201]. Additionally, intracellular nitrogen-fixing cyanobacteria are reported to coexist with dinoflagellate symbionts in the tissues of *Monastraea cavernosa* and to functionally express nitrogenase activity [202]. Corals also harbour a diverse assemblage of heterotrophic microorganisms in their skele-ton, tissues and lipid-rich mucus (reviewed in [203]), and these communities include large populations of diazo-trophic bacteria [204,205], and archaea [206]. Apart from nitrogen fixation, the coral microbiota contributes to other nitrogen-cycling processes, such as nitrification, ammonification and denitrification [207,208]. We were surprised to find several nitrogen fixation and cycling proteins encoded in the genome of *A. digitifera* (Table 8), notably a nitrogen fixation NifU-like protein, the Nif-specific regulatory protein (NifA), the regulatory NAD(+)-dinitrogen-reductase ADP-D-ribosylastransferase protein, a nitrifying ammonia monooxy-genase enzyme and nitrate reductase, which are usually expressed only by prokaryotic microorganisms.

**Table 8:** **Proteins of nitrogen metabolism in the predicted proteome of *A. digitifera***

		
**Gene sequence**	**KEGG Orthology**	**Encoded protein description**
v1.23444; v1.09133; v1.23443	K05521	ADP-ribosylglycohydrolase (DraG)
v1.09202	K10944	Ammonia monooxygenase subunit A
v1.03645 [+ 8 other sequence copies]	K03320	Ammonium transporter, Amt family
v1.12268; v1.12269	K06580	Ammonium transporter Rh
v1.02406	K01954	Carbamoyl-phosphate synthase (CPS)
v1.01524; v1.18283; v1.18284	K01948	Carbamoyl-phosphate synthase (CPS, ammonia)
v1.01615	K04016	Formate-dependent nitrite reductase (NrfA)
v1.16277; v1.23483; v1.13667; v1.22675	K00261	Glutamate dehydrogenase (NAD(P)+)
v1.17166; v1.11089	K01745	Histidine ammonia-lyase
v1.22825; v1.08034;v1.o8520	K05123	Integration host cell factor (INF) subunit beta
v1.11343	K05951	NAD+−dinitrogen-reductase ADP-D-ribosyltransferase (DraT)
v1.00547	K02584	Nif-specific regulatory protein (NifA)
v1.18869	K00371	Nitrate reductase 1, beta subunit
v1.06763	K08346	Nitrate reductase 2, beta subunit
v1.14858; v1.00685; v1.23148	K05916	Nitric oxide dioxygenase
v1.16954 [+ 5 other sequence copies]	K02448	Nitric oxide reductase NorD protein
v1.06115	K02164	Nitric oxide reductase NorE protein
v1.17629 [+ 12 other sequence copies]	K04748	Nitric oxide reductase NorQ protein
v1.24077 [+ 4 other sequence copies]	K13125	Nitric oxide synthase-interacting protein
v1.21801; v1.05719; v1.23577; v1.19464	K13253	Nitric-oxide synthase, invertebrate
v1.05980	K00363	Nitrite reductase (NAD(P)H) small subunit
v1.00101	K02598	Nitrite transporter NirC
v1.02355; v1.18772	K04488	Nitrogen fixation protein NifU
v1.17812	K02589	Nitrogen regulatory protein PII 1
v1.09150	K02570	Periplasmic nitrate reductase NapD
v1.01560	K02571	Periplasmic nitrate reductase NapE
v1.10035	K00218	Protochlorophyllide reductase [NifEN-like]
v1.08939	K03737	Pyruvate-flavodoxin reductase (NifJ)
v1.17373	K00365	Urate oxidase
v1.13217	K01427	Urease
v1.16409 [+ 5 other sequence copies]	K03187	Urease accessory protein
v1.13217	K01429	Urease subunit beta
v1.13217	K14048	Urease subunit gamma/beta
v1.12211 [+ 4 other sequence copies]	K00106	Xanthine dehydrogenase/oxidase
v1.12212	K13481	Xanthine dehydrogenase small subunit

(Excluding amino acid and pyrimidine/purine nucleotide synthesis or metabolism).

The presence of genes encoding proteins involved in nitrogen fixation raises speculation that corals may contribute directly to, or perhaps co-regulate, certain processes that catalyse the reduction of dinitrogen (N_2_) to ammonia (NH_3_) by the enzyme nitrogenase reductase (NifH). The functional NifH enzyme is a binary protein composed of a molybdenum-iron (MoFe) protein (NifB/NifDK), or its NifEN homologue, fused with a FeMo-cofactor (FeMoco) protein [209]. While genes encoding NifB, NifDK (or NifEN) and their FeMo-cofactor do not appear in the genome of *A. digitifera*, a gene encoding the NifEN-like protein protochlorophyllide oxidoreductase (POR) is present (Table 8). POR has all three subunits with high similarity to the assembled MoFe nitrogenase [210], but this homologue is unlikely to be effective in nitrogen reduction [211,212] since its activity is light dependent [213] when tissues are highly oxic [193]. The NifU protein encoded in the coral genome preassembles the metallocatalytic Fe-S clusters for maturation of nitrogenase [214], but its assemblage without NifS, a cysteine desulfurase needed for [Fe-S] cluster assembly [215], would be incomplete, and its pre-nitrogenase receptor is also missing. Yet, the coral does have the *nifJ* gene that encodes pyruvate:flavodoxin oxidoreductase required for electron transport in nitrogenase reduction [216]. The regulatory NifA protein encoded in the coral genome might activate, on stimulation by the integration host factor (INF), transcription of nitrogen fixation (*nif*) operons of RNA polymerase [217], and both of these proteins are encoded in the coral genome. Additional to this transcriptional control, post-translational nitrogenase activity is controlled by reversible ADP-ribosylation of a specific arginine residue in the nitrogenase complex [218]. NAD(+)-dinitrogen-reductase ADP-D-ribosyltransferase (DraT) inactivates the nitrogenase complex while ADP-ribosylgly-cohydrolase (DraG) removes the ADP-ribose moiety to restore nitrogenase activity, and both of these enzymes are encoded in the coral genome. Given that genes encoding essential constituent proteins of nitrogenase assembly appear incomplete, corals are unlikely to fix nitrogen *per se*, but co-opted elements of the coral genome to regulate processes of nitrogen fixation by its diazotrophic consortia is a prospect worthy of exploration [219].

Nitrofying/nitrifying bacteria and archaea express the enzyme ammonia monooxygenase that converts fixed ammonia to nitrite (via hydroxylamine) and the enzyme nitrite (oxido)reductase completes the oxidation of nitrite to nitrate, and both of these enzymes are entrained in the genome of *A. digitifera* (Table 8). The ammonia monooxygenase subunit A *(amoA*) of archaeal consorts has been described in nine species of coral from four reef locations [220], but the presence of *amoA* in the coral genome, together with encoded ammonium transport proteins, was not anticipated. Another protein of prokaryotic origin encoded in the coral genome is nitrate reductase (periplasmic, assimilatory and respiratory), the latter being required for anaerobic respiration by bacteria [221], and unlike the nitrate reductase family of sulphite oxidase enzymes in eukaryotes, the nitrate reductases of prokaryotes (K00363) belong to the DMSO reductase family of enzymes. Also encoded in the coral genome are a nitrite transporter (NirC) and a formate-dependent nitrite reductase (NrfA) required for nitrite ammonification [222]. In addition to nitrite reduction, NrfA reduces nitric oxide, hydroxylamine, nitrous oxide and sulphite, the last providing a metabolic link between nitrogen and sulphur cycling in coral metabolism. Other enzymes of nitrogen metabolism encoded in the coral genome are the carbamoyl-phosphate synthase family of enzymes [223] that catalyses the ATP-dependent synthesis of carbamoyl phosphate used for the production of urea (ornithine cycle) to provide a ready store of fixed-N in the urea-nitrogen metabolism of corals [224]. Another nitrogen source comes from glutamate dehydrogenase (GDH) that reversibly converts glutamate to α-ketoglutarate with liberation of ammonia, and as expected [225], this enzyme is encoded in the coral genome, together with the prokaryotic nitrogen regulatory protein PII of glutamine synthase, which in bacteria is activated in response to nitrogen availability. Encoded also is histidine ammonia-lyase (histidase) that liberates ammonia (and urocanic acid) from cytosolic stores of histidine. It is now accepted that uric acid deposits accumulated by symbiotic algae provide a significant store of nitrogen for the coral holobiont [226], so it is noteworthy that the coral genome encodes urate oxidase (uricase) to catalyse uric acid oxidation to allanotoin from which urea and ureidoglycolate are produced in a reaction catalysed by allantoicase (allantoate amidinohydrolase), both of which known isoforms are present in the coral genome. Encoded in the coral genome is also urease to catalyse the hydrolysis of urea, presumably excreted by its algal symbionts, with the release of carbon dioxide and ammonia to meet the nitrogen demand of the coral holobiont during periods of low nitrogen availability. Similarly, xanthine dehydrogenase (xanthine: NAD^+^-oxidoreductase) acts by oxidation on a variety of purines, including hypoxanthine, to yield urate for the recycling of nitrogen in coral nutrition. Many of the aforementioned proteins of nitrogen metabolism, including Nif proteins, have been detected in the proteome of an endosymbiont-enriched fraction of the coral *S. pistillata*[39].

Notwithstanding consideration of the rapid diffusion rate of nitric oxide (NO) or its apparent short biological half-life [227], there is debate about the provenance of endogenously produced NO in signalling the bleaching of corals in response to environmental stress. Elevated nitric oxide synthase (NOS) activity and NO production in algal symbionts has been attributed to the thermal stress response of corals [228,229], whereas the host is ascribed to be the major source of NO during exposure to elevated temperature [230,231]. While our annotation may not resolve this dispute, we show (Table 8) that nitric oxide synthase enzymes (Nor D, Nor E, Nor Q and an invertebrate NOS protein) are encoded in the genome of *A. digitifera*, together with a nitric oxide-interacting protein (NOIP) that in higher animals regulates neuronal NOS activity [232]. Nitric oxide is an intermediate of nitrite reduction catalysed by nitrite reductase (NIR), which by further reduction produces ammonia. The coral genome also encodes nitric oxide dioxygenase (NOD) that converts nitric oxide to nitrate. Accordingly, enhanced expression of NIR (NO reduction) or NOD (NO oxidation) could ameliorate the NO-signalling response of coral bleaching presumed activated by environmental stress.

### DNA repair

Cellular DNA is prone to damage caused by the products of normal metabolism and by exogenous agents. Damage to DNA from metabolic processes include the oxidation of nucleobases and strand interruptions by the production of reactive oxygen species (ROS), from alkylation of nucleotide bases, from the hydrolysis of bases causing deamination, depurination and depyrimidination, and from the mismatch of base pairs from errors in DNA replication. Damage affected by external agents include exposure to UV light causing pyrimidine dimerization and free radical-induced damage, exposure to ionising radiation causing DNA strand breaks, thermal disruption causing hydrolytic depurination and single-strand breaks, and by xenobiotic contamination to cause DNA adduct formation, nucleobase oxidation and DNA crosslinking. Most of these lesions affect structural changes to DNA that alter or prevent replication and gene transcription at the site of DNA damage. Thus, recognition and repair of DNA abnormalities are vital processes essential to maintain the genetic integrity of the coral genome. Since there are multiple pathways causing DNA damage at diverse molecular sites, there are likewise diverse and overlapping processes available to repair cellular DNA damage. Of the many nuclear repair processes, photoreactivation (photolyase), base excision repair and nucleotide excision repair are the main elements for the repair of cellular DNA damage.

Exposure to sunlight is an absolute requirement for phototrophic symbiosis, but excessive exposure of corals to solar ultraviolet radiation can inflict direct damage to DNA by pyrimidine dimerization and 6-4 photoadduct formation and cause indirect damage by the production of ROS to initiate free-radical damage. While there have been abundant studies on the sensitivity of corals to solar ultraviolet radiation, only a few have examined the effects of solar UV to cause DNA damage. Photoreactivation has been shown to be an important repair pathway for reversing UV-activated DNA damage in adult coral [233] and coral planulae [234]. UV damage to DNA was first demonstrated by the detection of unrepaired cyclobutane pyrimidine dimers (CPDs) in the host tissues and algal symbionts of the coral *Porities porites*, in which CPDs had increased in a UV dose-dependent manner [235], whereas CPDs and 6-4 pyrimidine-pyrimidone photoadducts in the coral *Montipora verrucosa* holobiont were correlated inversely with levels of coral “sunscreen” protection [236]. The effects of solar UV radiation causing DNA lesions in coral have been determined by use of the comet assay [237], and UV-induced DNA damage and repair has been examined in the symbiotic anemone *Aiptasia pallida*[238]. The comet assay showed also that DNA lesions in coral planulae had increased on acquiring algal symbionts, presumably from greater ROS production resulting as a by-product of photosynthesis [239]. Iron-induced oxidative stress was found likewise to enhance DNA damage in the coral *Pocillopora damicornis* as determined by the occurrence of DNA apurinic/apyrimidinic sites caused by hydrolytic lesions [240]. Significantly, DNA damage in the host and algal symbionts of the coral *Montastraea faveo-lata* was found to occur simultaneously during thermal “bleaching” stress, and DNA damage is further enhanced on exposure to greater irradiances of solar radiation [241]. Nevertheless, despite the serious risk of unrepaired DNA damage to coral survival, the DNA repair processes of corals to mitigate the detrimental effects of environmental stress have not been adequately characterised at the transcriptome level of expression [29,242].

Our annotation of the sequenced genome of *A. digitifera* has revealed genes encoding a large repertoire of DNA repairing enzymes and their adaptor proteins (Table 9). Given strong evidence for DNA photoreactivation in corals having been reported [233,234], it was surprising to find only one gene in single copy that encodes a sole photolyase enzyme for reversing pyrimidine dimer and 6-4 photoadduct formation. Notably, we found genes encoding 6 members of the ERCC family of nucleotide excision repair enzymes, together with the UV excision repair protein RAD23, for the repair of UV-induced DNA damage. More abundant are the DNA mismatch repair enzymes from the MLH, MSH, Mut and PMS protein families and related glycosylase/lyase proteins for repairing erroneous insertion, deletion and mis-incorporation of bases to arise during DNA replication and recombination. There is additionally a specific gene that encodes a 3′-endonuclease protein that has a preference to correct mispaired nucleotide sequences. Abundant also are other members of the RAD-family of DNA repair proteins, including 28 sequence copies of a gene encoding the RAD50 protein for DNA double-strand break repair that, together with members of the MRE, Rec, REV, Swi5/Sae3, XRCC and XRS families of recombination and polymerase proteins, have complementary roles in DNA repair. Apparent also in the genome are the DNA helicase proteins, including RuvB–like proteins, which are primarily involved in DNA replication and transcription, but assist also in the repair of DNA damage by separating double strands at affected sites of DNA damage to facilitate repair. Of the multiple families of ATP-dependent DNA helicase proteins encoded in the coral genome, RecQ and helicase Q predominate. Encoded in the coral genome are 5 homologues of the DNA repair alkB proteins that reverse damage to DNA from alkylation caused by chemical agents by removing methyl groups from 1-methyl adenine and 3-methyl cytosine products in single-stand DNA. Annotated also are genes encoding DNA ligase 3 for repairing single-strand breaks, DNA ligase 4 to repair double-strand breaks, and a DNA cross-link repair 1C protein with single-strand specific endonuclease activity that may serve in a proofreading function for DNA polymerase. Taken together, expressing this arsenal of DNA protection may provide corals with limited ability to transcribe gene-encoded adaptation to a changing global environment.

**Table 9:** **DNA repair proteins in the predicted proteome of *A. digitifera***

		
**Gene sequence**	**KEGG Orthology**	**Encoded protein description**
v1.02961; v1.13402	K03575	A/G-specific adenine glycosylase (MutY)
v1.11766	K03919	Alkylated DNA repair protein
v1.04821	K10765	Alkylated DNA repair protein alkB homologue 1
v1.02479	K10766	Alkylated DNA repair protein alkB homologue 4
v1.20302	K10767	Alkylated DNA repair protein alkB homologue 5
v1.24450	K10768	Alkylated DNA repair protein alkB homologue 6
v1.02766; v1.09413	K10770	Alkylated DNA repair protein alkB homologue 8
v1.01590 [+ 4 other sequence copies]	K10884	ATP-dependent DNA helicase 2 subunit 1
v1.18810; v1.03166; v1.08449	K10885	ATP-dependent DNA helicase 2 subunit 2
v1.08013	K03722	ATP-dependent DNA helicase DinG
v1.03542	K14635	ATP-dependent DNA helicase MPH1
v1.06737 [+ 5 other sequence copies]	K15255	ATP-dependent DNA helicase PIF1
v1.17360; v1.21235	K10899	ATP-dependent DNA helicase Q1
v1.01081 [+ 8 other sequence copies]	K10730	ATP-dependent DNA helicase Q4
v1.16859	K10902	ATP-dependent DNA helicase Q5
v1.11661 [+ 19 other sequence copies]	K03654	ATP-dependent DNA helicase RecQ
v1.20397	K03656	ATP-dependent DNA helicase Rep
v1.18049; v1.07731; v1.05830	K10905	ATR interacting protein
v1.01679	K01669	Deoxyribodipyrimidine photo-lyase
v1.03410; v1.12968; v1.00865; v1.16876	K10887	DNA cross-link repair 1C protein
v1.07474; v1.07473; v1.01809	K10610	DNA damage-binding protein 1
v1.13116; v1.03378; v1.16328	K10140	DNA damage-binding protein 2
v1.17099 [+ 5 other sequence copies]	K11885	DNA damage-inducible protein 1
v1.05469	K06663	DNA damage checkpoint protein
v1.02859; v1.14719; v1.21030; v1.10920	K04452	DNA damage-inducible transcript 3
v1.02191	K10844	DNA excision repair protein ERCC-2
v1.19108 [+ 5 other sequence copies]	K10843	DNA excision repair protein ERCC-3
v1.22267 [+ 4 other sequence copies]	K10848	DNA excision repair protein ERCC-4
v1.15137 [+ 5 other sequence copies]	K10846	DNA excision repair protein ERCC-5
v1.18550; v1.02606; v1.14935; v1.08831	K10841	DNA excision repair protein ERCC-6
v1.20045; v1.01844; v1.11724; v1.03203	K10570	DNA excision repair protein ERCC-8
v1.15430; v1.03058	K03658	DNA helicase IV
v1.00228 [+ 4 other sequence copies]	K11665	DNA helicase INO80
v1.00136; v1.0678; v1.21529	K10776	DNA ligase 3
v1.23293; v1.19418; v1.23430; v1.15721	K10777	DNA ligase 4
v1.19248	K07458	DNA mismatch endonuclease, patch repair protein
v1.19011	K08739	DNA mismatch repair protein MLH3
v1.11513; v1.11449	K08735	DNA mismatch repair protein MSH2
v1.14781	K08736	DNA mismatch repair protein MSH3
v1.05696; v1.22444; v1.19162	K08740	DNA mismatch repair protein MSH4
v1.04904	K08741	DNA mismatch repair protein MSH5
v1.15360; v1.19426; v1.08585	K08737	DNA mismatch repair protein MSH6
v1.02429 [+ 8 other sequence copies]	K03572	DNA mismatch repair protein MutL
v1.03990	K03555	DNA mismatch repair protein MutS
v1.14015	K07456	DNA mismatch repair protein MutS2
v1.08443	K10864	DNA mismatch repair protein PMS1
v1.15229	K10858	DNA mismatch repair protein PMS2
v1.08658; v1.14152; v1.01681	K15082	DNA repair protein RAD7
v1.16407 [+ 27 other sequence copies]	K10866	DNA repair protein RAD50
v1.22193	K04482	DNA repair protein RAD51
v1.02646; v1.22076	K10958	DNA repair protein RAD57
v1.15671 [+ 4 other sequence copies]	K04483	DNA repair protein RadA
v1.16193; v1.19033	K04485	DNA repair protein RadA/Sms
v1.16079; v1.07685	K04484	DNA repair protein RadB
v1.21363; v1.22360; v1.02900	K03584	DNA repair protein RecO (recombination protein O)
v1.18390	K03515	DNA repair protein REV1
v1.04705	K10991	DNA repair protein Swi5/Sae3
v1.13920; v1.03800; v1.16133	K10803	DNA repair protein XRCC1
v1.15052	K10879	DNA repair protein XRCC2
v1.09315 [+ 4 other sequence copies]	K10886	DNA repair protein XRCC4
v1.02733; v1.24592	K10868	DNA repair protein XRS2
v1.14551; v1.23176	K10873	DNA repair and recombination protein RAD52
v1.20503 [+ 4 other sequence copies]	K10875	DNA repair and recombination protein RAD54
v1.23173; v1.16050	K10877	DNA repair and recombination protein RAD54B
v1.07227; v1.08907; v1.09439; v1.02644	K10847	DNA repair protein complementing XP-A cells
v1.11534 [+ 5 other sequence copies]	K10865	Double-strand break repair protein MRE11
v1.07939	K03660	N-glycosylase/DNA lyase
v1.16163	K03652	3-Methyladenine DNA glycosylase
v1.07231	K10726	Replicative DNA helicase Mcm
v1.05482	K04499	RuvB-like protein 1 (pontin 52)
v1.19813	K11338	RuvB-like protein 2
v1.06890	K15080	Single-strand annealing weakened protein 1
v1.17193; v1.14087	K03111	Single-strand DNA-binding protein
v1.15575	K10800	Single-strand monofunctional uracil DNA glycosylase
v1.07134	K10992	Swi5-dependent recombination DNA repair protein 1
v1.13860	K03649	TDG/mug DNA glycosylase family protein
v1.14423; v1.14399; v1.05070	K03648	Uracil-DNA glycosylase
v1.23838	K10791	Three prime repair exonuclease 2
v1.19522	K10839	UV excision repair protein RAD23

### Stress response proteins

Annotation of the *A. digitifera* genome reveals a wide assortment of thermal shock proteins, molecular chaperones and other stress response elements that are given in (Table 10), excluding antioxidant and redox-protective proteins which are described in the next section. Heat shock proteins 70 kDa, 90 kDa, 110kDA, HspQ and HspX (the last two proteins being homologues of the bacterial heat shock factor sigma32 and α-crystallin, respectively) are encoded in the coral genome, together with several HSP gene transcription factors. HSPs play a role in various cellular functioning such as protein folding, intracellular protein trafficking and resistance to protein denaturation. HSP expression is usually increased on exposure to elevated temperatures and other conditions of biotic and abiotic stress that include infection, inflammation, metabolic hyperactivity, exposure to environmental toxicants, ultraviolet light exposure, starvation, hypoxia and desiccation [243]. HSPs and chaperones are transcriptionally regulated and are induced by heat shock transcription factors [244], of which there are several encoded in the coral genome. Since HSPs are found in virtually all living organisms, it is not surprising that cnidarian *hsp* transcription and protein expression (HSP60, HSP70 and HSP90) have been profiled as a stress determinant [245-250] and early warning indicator of coral bleaching [251-254]. The coral genome reveals also a cold shock protein encoded by the *cspA* gene family, but profiling its expression with other stress response proteins activated by sub-optimum cold temperatures [255] has not been reported. Additionally, the coral genome encodes transcription of a homologue of the universal stress protein A (UspA), a member of an ancient and conserved group of stress-response proteins [256,257], which have been studied mostly in bacteria [258] but have been described also in several plants [259] and animals, including members of the Cnidaria [260]. *Usp* transcripts have been quantified in the thermal stress response of the coral *Montastraea faveolata*[261] and its aposymbiotic embryos [262]. Another gene product of potential interest is a homologue of the oxidative-stress responsive protein 1 (OXSR1) that belongs to the Ser/Thr kinase family of proteins, as do other mitogen-stress activated protein kinases (MAPKs), that regulate downstream kinases in response to environmental stress [263] by interacting with the Hsp70 subfamily of proteins [264]. Another significant response protein encoded in the coral genome (Table 10) is a homologue of the stress-induced phosphoprotein 1 (30 domain sequence alignments), known also as the Hsp70-Hsp90 organising protein (HOP) belonging to the stress inducible (STI1) family of proteins, which is a principle adaptor protein that mediates the functional cooperation of molecular chaperones Hsp70 and Hsp90 [265,266]. It is yet to be determined if *Hop1* transcription may serve as a primary indicator of environmental stress in corals.

**Table 10:** **Stress response proteins in the predicted proteome of *A. digitifera***

		
**Gene sequence**	**KEGG Orthology**	**Encoded protein description**
v1.04616; v1.06277	K03694	ATP-dependent Clp protease subunit ClpA
v1.04617; v1.23486; v1.23484; v1.10207	K03695	ATP-dependent Clp protease subunit ClpB
v1.13464	K03697	ATP-dependent Clp protease subunit ClpE
v1.06903; v1.11461	K06891	ATP-dependent Clp protease adaptor protein ClpS
v1.12577; v1.09531; v1.17184	K03544	ATP-dependent Clp protease subunit ClpX
v1.09407	K08054	Calnexin (protein-folding chaperone)
v1.16781	K08057	Calreticulin (Ca^2+^-binding chaperone)
v1.04005	K10098	Calreticulin 3 (Ca^2+^-binding chaperone)
v1.02702[+ 5 other sequence copies]	K03704	Cold shock protein (beta-ribbon, CspA family)
v1.01907; v1.18998	K07213	Copper chaperone
v1.23457; v1.01713; v1.19228	K04569	Copper chaperone for superoxide dismutase
v1.08719; v1.19128	K09502	DnaJ homologue subfamily A member 1
v1.08719; v1.18432	K09503	DnaJ homologue subfamily A member 2
v1.16210; v1.22054	K09504	DnaJ homologue subfamily A member 3
v1.19128	K09505	DnaJ homologue subfamily A member 4
v1.04818 [+ 6 other sequence copies]	K09506	DnaJ homologue subfamily A member 5
v1.02841; v1.02842	K09507	DnaJ homologue subfamily B member 1
v1.00368; v1.13308; v1.16977; v1.03340	K09508	DnaJ homologue subfamily B member 2
v1.11537; v1.09205; v1.08628; v1.02840	K09511	DnaJ homologue subfamily B member 5
v1.24549 [+ 9 other sequence copies]	K09512	DnaJ homologue subfamily B member 6
v1.01573	K09513	DnaJ homologue subfamily B member 7
v1.00352; v1.09196; v1.06645	K09514	DnaJ homologue subfamily B member 8
v1.18536 [+ 4 other sequence copies]	K09515	DnaJ homologue subfamily B member 9
v1.14710	K09517	DnaJ homologue subfamily B member 11
v1.14959	K09518	DnaJ homologue subfamily B member 12
v1.09205	K09519	DnaJ homologue subfamily B member 13
v1.16242	K09520	DnaJ homologue subfamily B member 14
v1.20109; v1.03468	K09521	DnaJ homologue subfamily C member 1
v1.07111 [+ 5 other sequence copies]	K09522	DnaJ homologue subfamily C member 2
v1.21077 [+ 13 other sequence copies]	K09523	DnaJ homologue subfamily C member 3
v1.07739; v1.22910	K09524	DnaJ homologue subfamily C member 4
v1.01239 [+ 13 other sequence copies]	K09525	DnaJ homologue subfamily C member 5
v1.17629 [+ 29 other sequence copies]	K09527	DnaJ homologue subfamily C member 7
v1.18619; v1.08300; v1.23789	K09528	DnaJ homologue subfamily C member 8
v1.13575; v1.04213	K09529	DnaJ homologue subfamily C member 9
v1.05956; v1.05955; v1.21265; v1.21205	K09530	DnaJ homologue subfamily C member 10
v1.13525; v1.04120	K09531	DnaJ homologue subfamily C member 11
v1.09496 [+ 4 other sequence copies]	K09533	DnaJ homologue subfamily C member 13
v1.24546	K09534	DnaJ homologue subfamily C member 14
v1.05866	K09536	DnaJ homologue subfamily C member 16
v1.16151; v1.08307; v1.14980	K09537	DnaJ homologue subfamily C member 17
v1.16309	K09539	DnaJ homologue subfamily C member 19
v1.05241; v1.22999; v1.17372	K14258	Facilitated trehalose transporter (anhydrobiosis)
v1.12967; v1.19789	K14590	FtsJ methyltransferase [heat shock protein]
v1.02247	K09414	Heat shock transcription factor 1
v1.24112	K09416	Heat shock transcription factor 3
v1.05839	K09419	Heat shock transcription factor, other eukaryote
v1.12890 [+ 10 other sequence copies]	K03283	Heat shock 70 kDa protein 1/8
v1.07996	K09489	Heat shock 70 kDa protein 4
v1.02854; v1.07452; v1.01623	K09490	Heat shock 70 kDa protein 5
v1.14149; v1.14150	K09487	Heat shock protein 90 kDa beta
v1.07995; v1.07996; v1.16399; v1.11283	K09485	Heat shock protein 110 kDa
v1.08943; v1.05577	K11940	Heat shock protein HspQ
v1.00537; v1.00043	K03799	Heat shock protein HtpX
v1.01623	K04046	Hypothetical chaperone protein
v1.16216	K08268	Hypoxia-inducible factor 1 alpha
v1.08869; v1.15120	K09097	Hypoxia-inducible factor 1 beta
v1.22724	K09095	Hypoxia-inducible factor 2 alpha
v1.23698 [+ 16 other sequence copies]	K06711	Hypoxia-inducible factor prolyl 4-hydroxylase
v1.16737; v1.22345	K09486	Hypoxia up-regulated 1 (heat shock protein 70 family)
v1.10188	K08900	Mitochondrial chaperone BCS1
v1.17197; v1.04394	K04445	Mitogen-stress activated protein kinases
v1.16301; v1.21224; v1.19344	K04043	Molecular chaperone DnaK
v1.09682; v1.16748; v1.07471; v1.13624	K03687	Molecular chaperone GrpE
v1.01621; v1.04945; v1.15919	K04044	Molecular chaperone HscA
v1.18210	K04083	Molecular chaperone Hsp33
v1.17478; v1.16977; v1.10289; v1.19907	K04079	Molecular chaperone HtpG
v1.08895; v1.18099	K11416	Mono-ADP-ribosyltransferase sirtuin 6
v1.02024	K11411	NAD-dependent deacetylase sirtuin 1
v1.04813	K11412	NAD-dependent deacetylase sirtuin 2
v1.22049; v1.22211; v1.02221	K11413	NAD-dependent deacetylase sirtuin 3
v1.11849; v1.02221	K11414	NAD-dependent deacetylase sirtuin 4
v1.05495	K11415	NAD-dependent deacetylase sirtuin 5
v1.04868	K11417	NAD-dependent deacetylase sirtuin 7
v1.15070 [+ 4 other sequence copies]	K08835	Oxidative-stress responsive protein 1 (OXSR1)
v1.04503	K11875	Proteasome assembly chaperone 1
v1.01531	K11878	Proteasome assembly chaperone 4
v1.01210	K11879	Proteasome chaperone 1
v1.18611	K11880	Proteasome chaperone 2
v1.00599 [+ 29 other sequence copies]	K09553	Stress-induced-phosphoprotein 1 (HOP1)
v1.08830	K13057	Trehalose synthase (anhydrobiosis)
v1.22042	K03533	TorA specific chaperone
v1.16986 [+ 7 other sequence copies]	K06149	Universal stress protein A

Molecular chaperones are a diverse family of proteins expressed by both prokaryotic and eukaryotic organisms that serve to maintain correct protein folding in a 3-dimensional functional state, assist in multiprotein complex assembly and protect proteins from irreversible aggregation at synthesis and during conditions of cellular stress [267]. Additionally, heat shock proteins and their co-chaperones may regulate cell death pathways by inhibition of apoptosis [268]. The coral genome encodes a large number of DnaJ subfamily (J-domain) chaperones (Hsp40) that with co-chaperone GrpE (Table 10) regulates the ATPase activity of Hsp70 (DnaK in bacteria) to enable correct protein folding [269]. The coral genome encodes homologues of the molecular chaperones HscA (specialised Hsp70), the redox-regulated chaperone Hsp33, HtpG (high temperature protein G), members of the calnexin/calreticulin chaperone system of the endoplasmic reticulum, a mitochondrial chaperone BCS1 protein necessary for the assembly of the respiratory chain complex III and a specific chaperone of trimethyl N-oxide reductase (TorA). The coral genome also encodes hypoxia-inducible factors (HIFs) that moderate the deleterious effects of hypoxia on cellular metabolism (reviewed in [270]). In the HIF signalling cascade, the alpha subunits of HIF are hydroxylated at conserved proline residues by HIF prolyl-hydroxylases allowing their recognition for pro-teasomal degradation, which occurs during normoxic conditions but is repressed by oxygen depletion. Hypoxia-stabilised HIF1 upregulates the expression of enzymes principally of the oxygen-independent glycolysis pathway, and in higher animals promotes vascularisation, whereas the mammalian HIF2 paralogue regulates erythropoietin control of hepatic erythrocyte production in response to hypoxic stress [271]. The roles of HIF1 and HIF2 homologues in corals have been established, with HIF1 regulation of glycolysis critical to metabolic function during the dark diurnal anoxic state of coral respiration [193,272].

Heat shock proteins that repair unfolded or misfolded protein have a complementary function to the ubiquitin-proteasome system (ubiquitins not tabulated) that selects damaged protein for degradation [273], such that HSP chaperones and the proteasome act jointly to preserve cellular proteostasis [274,275]. Thus, several proteasome chaperones and assembly chaperones are encoded in the *A. digitifera* genome (Table 10). While proteasome chaperones serve to target aberrant proteins for ubiquination, the proteasome chaperones facilitates 20S assembly for biogenesis of the multiunit 26S proteasome that is activated in response to stress [276,277], possibly by FtsJ (aka RrmJ), a well-conserved heat shock protein having novel ribosomal methyltransferase activity that targets methylation of 26S rRNA under heat shock control [278,279]. The HspQ protein encoded in the coral genome, although studied almost exclusively in bacteria, is known to stimulate degradation of denatured proteins caused by hyperthermal stress, particularly DnaA that initiates DNA replication in prokaryotes [280]. Specifically, HspQ (heat shock factor sigma32) regulates the expression of Clp ATPase-dependent protease family enzymes [281,282], of which ClpA, ClpB, ClipE, the protease adaptor protein ClpS [283] and the unfoldase ClpX protein [284] are encoded in the coral genome (Table 10). HspX is a small 16 kDa α-crystallin chaperone (Acr) protein belonging to the Hsp20 family of proteins [285] that suppresses thermal denaturation and aggregation of proteins [285]. Significantly, Acr proteins are known to bind with carbonic anhydrase [286] and may have importance in moderating stress-induced loss of calcium deposition. Thus, HspX/Acr expression may account for differences in the thermal sensitivity of corals to calcification that varies among genera [287]. In a different context, HspX is attracting considerable attention for its potential to elicit long-term protective immunity against human *Mycobacterium tuberculosis* infection by chaperoning a host-protective antigen [288] that by extension, but yet untested, may likewise repress virulence in the initiation and progression of microbial coral disease [289,290].

The coral genome encodes complete membership of the human sirtuin (SIRT1-7) family of NAD(+)-dependent protein deacetylases and ADP-ribosyltransferases. Mammalian SIRT1 (a homologue of yeast Sir2) is an important regulator of metabolism, cell differentiation, stress response transcription and pathways of cellular senescence (reviewed in [291]). SIRT proteins regulate chromatin function through deacetylation of histones that promote subsequent alterations in the methylation of histones and DNA to affect, via deactivation of nuclear transcription factors and co-regulators, epigenetic control of nuclear transcription. As NAD^+^-dependent enzymes, SIRT1 can regulate gene expression in response to cellular NAD^+^/NADH redox status providing a metabolic template for epigenetic transcriptome reprogramming [292,293]. In the human genome repertoire, SIRT1 modulates cellular responses to hypoxia by deacetylation of HIF1α [294] and inhibits nitric oxide synthesis by suppression of the nuclear factor-kappaB (NF-κB) signalling pathway [295], SIRT2 promotes oxidative stress resistance by deacetylation of forkhead box O (FOXO) proteins [296], SIRT3 decreases ROS production in adipocytes [297], SIRT4 regulates fatty acid metabolism and stress-response elements of mitochondrial gene expression [298], SIRT5 is a protein lysine desuccinylase and demalonylase of unknown function [299], SIRT6 activates base-excision repair [300] and SIRT7 inhibits apoptosis induced by oxidative stress by deacylation of p53 [301,302]. The significance of coral SIRT proteins, by analogy, to exert stress tolerance is yet to be examined.

Metallochaperones are an important class of enzymes that transport co-factor metal ions to specific proteins [303]. The copper chaperone protein ATX1 (human ATOX1) delivers cytosolic copper to Cu-ATPase proteins and serves as a metal homeostasis factor to prevent Fenton-type production of highly reactive hydroxyl radicals. ATX1, which is strongly induced by molecular oxygen, functions additionally as an antioxidant to protect cells against the toxicity of both the superoxide anion and hydrogen peroxide [304]. Encoded also is a specific copper chaperone essential to the activation of Cu/Zn superoxide dismutase [305,306] that is enhanced by photooxidative stress in scleractionian corals [307], although reported to be less pronounced in the host than in symbiotic algae [308]. In addition to high light exposure, reef-building corals of shallow reef flats are occasionally exposed to the atmosphere for periods that can last several hours during extreme low tides. Hence, species that are adapted to withstand acute desiccation (anhydrobiosis) have a better chance of surviving such conditions. The disaccharide trehalose is an osmolyte that in some plants and animals allows them to survive prolonged periods of desiccation [309]. The hydrated sugar has high water retention that forms a gel phase when cells dehydrate, which on rehydration allows normal cellular activity to resume without damage that would otherwise follow a dehydration/rehydration cycle. Furthermore, trehalose is highly effective in protecting enzymes in their native state from inactivation from thermal denaturation [310]. Given that *A. digitifera* is endemic on shallow reef flats prone to exposure at low tides [311], it is not surprising that the coral genome encodes trehalose synthase and a facilitated trehalose transporter for protection against dehydration.

### Antioxidant and redox-protective proteins

Oxygen is vital for life, but it can also cause damage to cells, particularly at elevated levels. In coral symbiosis, the photosynthetic endosymbionts of corals typically produce more oxygen than the holobiont is able to consume by respiration, so that coral tissues are hyperoxic with tissue *p*O_2_ levels often exceeding 250% of air saturation during daylight illumination [193]. Furthermore, because algal symbionts reside within the endodermal cells of their host, coral tissues must be transparent to facilitate the penetration of downwelling light required for photosynthesis by their algal consorts. In clear shallow waters this entails concurrent exposure to vulnerable molecular sites of both partners to damaging wavelengths of ultraviolet radiation. The synergistic effects of tissue hyperoxia and UV exposure can cause oxidative damage to the symbiosis via the photochemical production of cytotoxic oxygen species [312] that are produced also during normal mitochondrial function [313]. Consequently, protective proteins (antioxidant enzymes) are expressed to maintain the fine balance between oxygen metabolism and the production of potentially toxic reactive oxygen species (ROS). If this balance is not maintained by regulation of oxidative and reductive processes (redox regulation), oxidative stress occurs by the generation of excess ROS, causing damage to DNA, proteins, and lipids. Corals elaborate a variety of molecular defences that including the production of UV-protective sunscreens, (MAAS), antioxidants, antioxidant enzymes, chaperones and heat shock proteins, which are often inducible under conditions of enhanced oxidative stress [307], including conditions that elicit coral bleaching [314,315]. An excellent review on the formation of ROS and the role of antioxidants and antioxidant enzymes in the field of redox biology is given by Halliwell [316].

Annotation of the *A. digitifera* genome reveals sequences encoding two isoforms of the antioxidant enzyme superoxide dismutase (SOD) from both the Cu/Zn and Fe/Mn families of SOD (Table 11). These metalloprotein enzymes catalyse the dismutation of superoxide to yield molecular oxygen and hydrogen peroxide, the latter being less harmful than superoxide. Superoxide can oxidize proteins, denature enzymes, oxidize lipids and fragment DNA. By removing superoxide, SOD protects also against the production of reactive peroxynitrite formed by the combination of superoxide and nitric oxide, which is a precursor reactant for production of the supra-reactive hydroxyl radical. Hydrogen peroxide *per se* is a mild oxidant, but it readily oxidises free cellular ferrous iron to ferric iron with production of hydroxyl radicals via the Fenton reaction. Accordingly, both the removal of hydrogen peroxide and the expression of proteins, such as transferrin, (bacterio)ferritins and metallothioneins, that bind reactive (transition) metal ions is important to protect cellular components from acute oxidative damage. Oddly, only a metallothionein expression activator was found encoded in the coral genome without finding a sequence to activate transcription of the actual metallothionein protein gene.

**Table 11:** **Antioxidant and redox-protective proteins in the predicted proteome of *A. digitifera***

		
**Gene sequence**	**KEGG Orthology**	**Encoded protein description**
v1.10918	K04756	Alkyl hydroperoxide reductase subunit D
v1.11551	K03387	Alkyl hydroperoxide reductase subunit F
v1.07812	K03594	Bacterioferritin
v1.21362 [+ 4 other sequence copies]	K00429	Catalase (bacterial)
v1.17525 [+ 4 other sequence copies]	K03781	Catalase (peroxisonal)
v1.23457; v1.01713; v1.19228	K04569	Copper chaperone for superoxide dismutase
v1.20153; v1.20154	K10528	Hydroperoxide lyase
v1.19687; v1.19688; v1.18796; v1.18795	K00522	Ferritin heavy chain
v1.06441	K03674	Glutaredoxin 1
v1.19449	K03675	Glutaredoxin 2
v1.14929 [+ 5 other sequence copies]	K03676	Glutaredoxin 3
v1.13285; v1.03722; v1.03688; v1.10496	K00432	Glutathione peroxidase
v1.13174; v1.13775; v1.05473	K00383	Glutathione reductase (NADPH)
v1.14344; v1.19399; v1.01421	K01920	Glutathione synthase
v1.02173	K09238	Metallothionein expression activator
v1.09719; v1.16134; v1.18608	K07390	Monothiol glutaredoxin
v1.14890; v1.17685	K07305	Peptide-methionine (R)-S-oxide reductase
v1.14909	K00435	Peroxiredoxin
v1.14106	K13279	Peroxiredoxin 1
v1.08691	K11187	Peroxiredoxin 5, atypical 2-Cys peroxiredoxin
v1.01410	K11188	Peroxiredoxin 6, 1-Cys peroxiredoxin
v1.03688	K05361	Phospholipid-hydroperoxide glutathione peroxidase
v1.05148	K05905	Protein-disulfide reductase
v1.02922; v1.22772; v1.24164	K05360	Protein-disulfide reductase (glutathione)
v1.06810	K12260	Sulfiredoxin
v1.01713 [+ 4 other sequence copies]	K04565	Superoxide dismutase, Cu/Zn family
v1.09974; v1.20324	K04564	Superoxide dismutase, Fe/Mn family
v1.02378	K11065	Thiol peroxidase, atypical 2-Cys peroxiredoxin
v1.22324 [+ 7 other sequence copies]	K03671	Thioredoxin 1
v1.05148; v1.03230; v1.20699	K03672	Thioredoxin 2
v1.17881 [+ 5 other sequence copies]	K13984	Thioredoxin domain-containing protein 5
v1.04532; v1.24501	K09585	Thioredoxin domain-containing protein 10
v1.11551; v1.19049	K00384	Thioredoxin reductase (NADPH)
v1.10930	K14736	Transferrin

As expected from the foregoing, the genome of *A. digitifera* encodes the antioxidant enzyme catalase (CAT) that is highly efficient in decomposing hydrogen peroxide to yield molecular oxygen and water. Two isoforms of CAT are encoded at multiple sites. One is a peroxisomal eukaryotic CAT enzyme that targets the removal of hydrogen peroxide formed as a by-product of oxidase enzymes, and the other is a related catalase domain-containing protein presumed also to decompose hydrogen peroxide. Glutathione peroxidise (GPx) reduces both hydrogen peroxide and lipid hydroperoxides, the latter of which are formed by radical-induced lipid autoxidation. Phototrophic organisms, including higher plants, utilise ascorbate peroxidase (APx) as a primary catalyst for the reduction of hydrogen peroxide and lipid hydroperoxides. However, unlike the freshwater cnidarian *H. viridis*[164], there is no evidence for transfer of APx-encoding genes to *A. digitifera*. The antioxidant enzymes SOD, CAT, GPx and APx are well characterised in the algal and animal partners of coral symbiosis (reviewed in [317]). Additionally, the coral genome has sequences encoding alkyl hydroperoxide reductase, hydroperoxide lyase, phospholipid-hydroperoxide glutathione peroxidase, thiol peroxidase and multiple isoforms of peroxiredoxin, all of which function in the detoxification of organo-hydroperoxides that are produced as a by-product of aerobic metabolism. Additionally, sulfiredoxin (Table 11) repairs peroxiredoxins when these enzymes are inhibited by over-oxidation [318].

Thioredoxins and glutaredoxins have important secondary roles in regulating multiple pathways in many biological processes, including redox signalling of apoptotic pathways, which have been attributed to processes involved in coral bleaching [56]. Other enzymes that regulate cellular thiol-disulfide homeostasis in this coral are monothiol glutaredoxin and protein-disulfide reductase. The coral genome encodes the ubiquitous thioredoxin system of antioxidant proteins (Table 11) that act as electron donors to peroxidases and ribonucleotide reductase (the latter not tabulated). By cysteine thiol-disulfide exchange, thioredoxins function as a protein thiol-disulfide oxidoreductase [319]. In the thioredoxin system, thioredoxins are maintained in their reduced state by NADPH-dependent, flavoenzyme thioredoxin reductase [320]. Peptide-methionine (R)-S-oxide reductase can additionally rescue thioredoxin from oxidative inactivation by disulfide reduction. Related glutaredoxins share many of the functions of thioredoxins but are reduced directly by glutathione, rather than by a specific reducing enzyme, while in turn glutathione is kept in its native state by NADPH: glutathione reductase.

In recent years there has been a particular focus on the role of ROS in coral bleaching, fuelled by dire prediction of future catastrophic episodes caused by environmental change affected by global warming [321]. Early predictions of coral bleaching were based principally on physical environmental parameters, rather than on the determination of the physiological state of coral populations to such conditions. While gene expression markers are being developed to monitor sub-bleaching levels of stress *in situ* (e.g., [261]), Kenkel et al. [322] opined that the current challenge for implementing expression-based methods lies in identifying coral genes demonstrating the most pronounced and consistent stress response, preferably with a large dynamic range to enable reliable quantification. To this end, we offer in Table 11 the annotation of novel redox-related genes for examination as potential candidate biomarkers to monitor the physiological response of *A. digitifera* to environmental stress.

### Proteins of cellular apoptosis

Apoptosis is the signalling of programmed cell death (PCD) that occurs in multicellular organisms in response to cellular injury. A key feature of apoptosis is the activation of endogenous endonucleases causing nuclear fragmentation, chromatin condensation and chromosomal DNA fragmentation, which typically presents in affected cells by the morphological appearance of plasma membrane blebbing and cell shrinkage. Caspases and related family member proteases are described as “executioners” of apoptosis that on post-translational activation degrade the regulatory proteins that prevent DNA degradation. Fragmentation of nuclear DNA is one of the hallmarks of apoptotic cell death that occurs by PCD stimuli in a wide variety of proliferating cells. NF-κB is a protein complex that controls the transcription of DNA that can induce the expression of nitric oxide synthesis (NOS) to produce NO that is a well-known promoter of the of the pro-apoptotic transcription factor p53 cell-cycle gatekeeper of the caspase cascade. In contrast to necrosis, which is the outcome of PCD, apoptosis mediates the fragmentation of damaged cells, which by phagocytosis are removed or degraded in phagolysosomes to spare surviving cells from the uncontrolled release of cytotoxic agents. Proteins of the caspase-mediated apoptotic cascade are regarded as products of constituent housekeeping genes that are necessary to maintain healthy multicellular function [323]. In the progression of cnidarian bleaching, apoptotic pathways are activated [322-325], but not all corals that suffer bleaching are destined to die [326,327]. Coral survival has been attributed to having a high level of apoptotic protection at the onset of coral bleaching [328] and during post-bleaching recovery [329] by specific activation of anti-apoptotic Bcl-2 proteins in surviving cells [330].

Cnidarians have a complex apoptotic protein network that has exceptional ancestral complexity and is comparable to that of higher vertebrates [331,332]. Cnidarian metamorphosis is tightly coupled with caspase-dependent apoptosis [333] and subsequent host-symbiont selection by post-phagocytic winnowing of *Symbiodinium* genotypes during the establishment of coral-dinoflagellate mutualism [334]. As expected, the coral genome of *A. digitifera* encodes multiple isoforms of genes that transcribe the caspase family of apoptotic effectors (Table 12). Included in this signalling pathway are the pro- and anti-apoptotic Bax/Bcl regulators and Bcl-2 athanogene (DNA-binding) activators of apoptosis. Notable in our annotation dataset are multiple genes that encode the protein domains of the apoptotic protease-activating factor (Apaf) that triggers assembly of the apoptosome leading to caspase activation [335]. Additional to this arsenal of cell cycle regulators are the death associated protein-6 (DAXX), a Fas-binding adaptor of c-Jun N-terminal kinase (JNK) activation [336], death-associated protein kinase (DAPK), a mediator of calcium/calmodulin-regulated Ser/Thr kinase [337], and the programmed cell death 6-interacting protein (PDCD6IP), which binds to PDCD-6 for execution of apoptosis via the caspase-3 pathway [338]. PDCD6IP activation of apoptosis is an enigma since PDCD-6 is not encoded in the coral genome, nor is caspase-3. Other cell cycle regulators are the p53 binding and p53-associated parkin-like proteins, and the activating TP53 regulating kinase protein and TP53 apoptosis effector of *TP53* gene expression.

**Table 12:** **Proteins of cellular apoptosis in the predicted proteome of *A. digitifera***

		
**Gene sequence**	**KEGG Orthology**	**Encoded protein description**
v1.17521; v1.02505; v1.20702; v1.05077	K02159	Apoptosis regulator BAX (BCL2-associated)
v1.05086; v1.20659	K02161	Apoptosis regulator BCL-2
v1.17522; v1.00181; v1.10817; v1.20703	K02163	Apoptosis regulator BCL-W
v1.05147 [+ 6 other sequence copies]	K12875	Apoptotic chromatin condensation inducer
v1.22264 [+ 72 other sequence copies]	K02084	Apoptotic protease-activating factor (Apaf)
v1.17326; v1.20305; v1.11586	K09555	BCL2-associated athanogene 1
v1.08601	K09558	BCL2-associated athanogene 4
v1.02839	K09559	BCL2-associated athanogene 5
v1.01518	K13087	BCL2-associated transcription factor 1
v1.20278; v1.00172; v1.07858	K14021	BCL-2 homologueous antagonist/killer
v1.09624	K02561	BCL2-related (ovarian) killer protein
v1.17749	K08573	Calpain-3
v1.00595; v1.14671; v1.00040	K08574	Calpain-5
v1.00040	K08575	Calpain-6
v1.19153; v1.17749	K08576	Calpain-7
v1.15226	K04740	Calpain-12
v1.02951	K08582	Calpain-15
v1.11167; v1.06681; v1.20230; v1.01376	K08585	Calpain, invertebrate
v1.0312 7 [+ 6 other sequence copies]	K08583	Calpain, small subunit 1
v1.17229; v1.00023; v1.09976	K02186	Caspase 2
v1.11989 [+ 5 other sequence copies]	K04397	Caspase 7
v1.02756 [+ 27 other sequence copies]	K04398	Caspase 8
v1.01818	K04399	Caspase 9
v1.00817 [+ 4 other sequence copies]	K04400	Caspase 10
v1.02005	K04741	Caspase 12
v1.00818 [+ 11 other sequence copies]	K04489	Caspase apoptosis-related cysteine protease
v1.13260	K07367	Caspase recruitment domain-containing protein 11
v1.06297 [+ 44 other sequence copies]	K02832	CASP2 and RIPK1 adaptor with death domain
v1.21531	K02308	Death-associated protein 6 (DAXX)
v1.09448; v1.15529; v1.20164	K08803	Death-associated protein kinase (DAPK)
v1.23110; v1.14222; v1.03658	K12366	Engulfment and motility protein 1 (phagocytosis/apoptosis)
v1.18448 [+ 78 other sequence copies]	K02373	Fas (TNFRSF6)-associated via death domain (FADD)
v1.24288 [+ 66 other sequence copies]	K10130	Leucine-rich repeats and death domain-containing protein
v1.20620	K04734	NF-kappa-B inhibitor alpha
v1.01706	K14214	NF-kappa-B inhibitor delta
v1.10378; v1.10729; 1.05609; v1.05609	K05872	NF-kappa-B inhibitor epsilon
v1.17893; v1.22419; v1.00700; v1.08415	K09256	NF-kappa-B inhibitor-like protein 1
v1.04158 [+ 211 other sequence copies]	K09257	NF-kappa-B inhibitor-like protein 2
v1.05320; v1.06979; v1.04467; v1.21371	K02580	Nuclear factor NF-kappa-B p105 subunit
v1.20334; v1.22743	K11970	p53-Associated parkin-like cytoplasmic protein
v1.14920; v1.11864; v1.15271; v1.11865	K06643	p53-Binding protein
v1.04289	K06708	Programmed cell death 1 ligand 2
v1.05882 [+ 7 other sequence copies]	K12200	Programmed cell death 6-interacting protein (PDCD6!P)
v1.10959; v1.04994	K04727	Programmed cell death 8 apoptosis-inducing factor
v1.16714	K06875	Programmed cell death protein 5 (PDCD-5)
v1.13112	K03171	Tnfrsf1a-associated via death domain
v1.24655; v1.12385	K10136	TP53 apoptosis effector
v1.09087	K08851	TP53 regulating kinase
v1.05030; v1.07044	K11859	Tumor necrosis factor, alpha-induced protein 3
v1.22799	K04389	Tumor necrosis factor ligand superfamily member 6
v1.05776	K05470	Tumor necrosis factor ligand superfamily member 7
v1.13754	K05472	Tumor necrosis factor ligand superfamily member 9
v1.21776 [+ 6 other sequence copies]	K04721	Tumor necrosis factor ligand superfamily member 10
v1.04001	K05473	Tumor necrosis factor ligand superfamily member 11
v1.19776	K05474	Tumor necrosis factor ligand superfamily member 12
v1.09015; v1.14041	K03158	Tumor necrosis factor receptor superfamily member 1A
v1.07010	K05141	Tumor necrosis factor receptor superfamily member 1B
v1.19735	K05142	Tumor necrosis factor receptor superfamily member 4
v1.13754	K03160	Tumor necrosis factor receptor superfamily member 5
v1.22577	K05143	Tumor necrosis factor receptor superfamily member 6B
v1.20003	K05144	Tumor necrosis factor receptor superfamily member 7
v1.23750; v1.17970; v1.19022	K05146	Tumor necrosis factor receptor superfamily member 9
v1.07527	K05148	Tumor necrosis factor receptor superfamily member 11B
v1.10221	K05151	Tumor necrosis factor receptor superfamily member 13C
v1.14826; v1.01054	K05152	Tumor necrosis factor receptor superfamily member 14
v1.09514	K05156	Tumor necrosis factor receptor superfamily member 19
v1.01640	K05161	Tumor necrosis factor receptor superfamily member 26
v1.08207; v1.16237; v1.14824	K10133	Tumor protein p53-inducible protein 3

Our genome annotation reveals 73 sequence matches for expressing the Apaf protein domain that, in conjunction with a high copy number for expressing caspase-8 (28 protein sequence matches), may enhance coral survival during embryogenesis by suppressing receptor-induced protein kinase (45 sequence matches) during early development [339]. The most conserved function of the CAPS2/RIPK adaptor (45 sequence matches) encoded in the coral genome is its essential regulation of apoptosis [340]. We find a wide repertoire of genes that additionally encode proteins that mediate apoptosis (Table 12). Amongst these are the calpain Ca^+2^-sensing family of proteins that initiate the signalling of apoptotic pathways [341]. There are 79 matches to sequences that encode the tumor necrosis Fas superfamily member 6 (TNFRSF6) receptor, which coupled with the death domain (FADD) protein is a cell signalling mediator for recruitment of caspase-8 that activates the apoptotic cysteine protease cascade. Coincident in the genome are 67 sequences encoding the leucine-rich repeat and death domain-containing (LRDD) adaptor that, by interacting with other p53-inducible death domain-containing (PIDD) proteins such as FADD, induces the caspase-2 pathway of apoptosis in response to DNA damage [342]. Elements of the NF-κB signalling pathway of cnidarians are highly conserved traits [343], which includes the caspase cascade and the pro-apoptotic and anti-apoptotic Bcl-2 family of proteins [344]. The coral genome of *A. digitifera* encodes the pleiotropic nuclear factor NF-κB p105 subunit, and astonishingly there are 212 sequence matches to the NF-κB inhibitor-like protein 2 domain with fewer matches to the NF-κB inhibitor-like protein 1 and NF-κB family inhibitors alpha, delta and epsilon. Evident in our genome annotation is the tumor necrosis factor-alpha induced protein 3 (TNFAIP3), a cytokine produced by activated (inflammatory) macrophages. Although TNF cytokines are a major extrinsic mediator of cellular apoptotic pathways, the precise function of the superfamily members of TNF ligands and receptors (Table 12) remains elusive in coral symbiology.

### Microbial symbiosis and pathogenicity

It is well established that corals associate with a vast consortia of microbes, including phototrophic symbionts (*Symbiodinium* spp.) and other eukaryotic microbionts, cyanophytes, heterotrophic bacteria, archaea and viruses [345]. Corals harbour diverse and abundant prokaryotic communities with distinct populations residing in separate habitats of the host skeleton, tissues and surface mucus layer (reviewed in [203]). Microbial populations are dominated by a few coral-specific taxonomic traits [346], but the majority of the population comprises a high number of taxonomically diverse, low-abundance ribotypes [347] with much of the diversity within the coral microbiome belonging to the “rare” biosphere [348,349]. The coral microbiome is vital to the nutrition and health of the holobiont [350] and contributes significantly to the protection of coral reef ecosystems against the detrimental effects of organic enrichment [351,352]. One emerging threat to coral reefs is the outbreak of infectious diseases (reviewed in [353]). Although highly subjective and with little experimental evidence to date, the coral probiotic hypothesis [354] suggests that the coral prokaryotic microbiome can adapt to changing environmental conditions by selective microbial reorganisation to impart greater resistance to disease and pathogen-mediated bleaching [355]. Whether the coral microbiome can respond to changing environmental conditions more rapidly than by host genetic mutation and selection based on contemporary phenotypic evolution on ecological time-scales [356], is a topic of current debate [357].

Corals, like other invertebrates, have an innate immune system based on self-histocompatibility recognition (reviewed in [358]), but to date few adaptive components have been identified [359]. Corals do not produce antibodies and thus lack a true adaptive immune system. Nonetheless, corals once susceptible to infection and bleaching caused by a specific bacterial agent can become immune to the invading pathogen by a phenomenon termed “experience-mediated tolerance”, a precept of the hologenome theory of evolution [360], although how this process occurs is largely unknown. In our annotation of the genome sequence of *A. digitifera* we uncovered genes encoding the expression of disease resistance proteins (Table 13), two of which match the plant RPM1 and RPS2 pathogen resistance proteins that guard against disease by binding with pathogen avirulence receptors [360,361]. Significant also is a gene to express the pathogenesis-related protein PR-1 (29 sequence domain matches) that is inducible in plants for systemic acquired resistance to pathogenic invasion [362]. We uncovered also multiple genes encoding the expression of myeloperoxidase (MPO) enzymes. MPOs produce hypochlorous acid from hydrogen peroxide and chloride ion (requiring heme as a cofactor), and it oxidizes tyrosine to the tyrosyl radical using hydrogen peroxide as an oxidizing agent. Hypochlorous acid and tyrosyl radicals are strong cytotoxic agents that in higher organisms are used as a primary defence by neutrophils to protect against invading pathogens. Phenoloxidase (tyrosinase) activity is reported to contribute to the innate defence system of *A. millepora* and *Porites* sp. [363] via activation of the melanin-signalling pathway that is induced in response to coral bleaching and localised disease [364,365]. Three genes of *A. digitifera* encode tyrosinase enzymes (data not tabulated) to account for the phenoloxidase activity reported in corals.

**Table 13:** **Microbial symbiosis and pathogenicity proteins in the predicted proteome of *A. digitifera***

**Gene sequence**	**KEGG Orthology**	**Encoded protein description**
v1.06126	K13061	Acyl homoserine lactone synthase
v1.19990	K01372	Bleomycin hydrolase
v1.00209; v1.06178	K03587	Cell division protein FtsI (penicillin-binding protein 3)
v1.18860	K13458	Disease resistance protein
v1.16231; v1.00374; v1.08191	K13457	Disease resistance protein RPM1
v1.13482 [+ 4 other sequence copies]	K13459	Disease resistance protein RPS2
v1.07889	K12090	Cag pathogenicity island protein 5
v1.24345	K12091	Cag pathogenicity island protein 6
v1.18924; v1.17622	K12093	Cag pathogenicity island protein 8
v1.05278	K12096	Cag pathogenicity island protein 11
v1.02083	K12104	Cag pathogenicity island protein 19
v1.12907	K12109	Cag pathogenicity island protein 24
v1.00209; v1.06178	K03587	Cell division protein FtsI (penicillin-binding protein 3)
v1.13874	K07259	Carboxy/endopeptidase (penicillin-binding protein 4)
v1.12514; v1.09758	K04127	Isopenicillin-N epimerase
v1.21332	K04126	Isopenicillin-N synthase
v1.07742	K02547	Methicillin resistance protein
v1.17478; v1.16977; v1.10289; v1.19907	K04079	Molecular chaperone HtpG (anti-bacterial)
v1.08255	K13651	Motility quorum-sensing regulator, GCU-specific toxin
v1.14792 [+ 7 other sequence copies]	K10789	Myeloperoxidase
v1.02333 [+ 26 other sequence copies]	K13449	Pathogenesis-related protein 1
v1.05017	K03693	Penicillin-binding protein
v1.17507	K12556	Penicillin-binding protein 2X
v1.13874	K07259	Penicillin-binding protein 4
v1.16655	K02171	Penicillinase repressor
v1.14688	K15126	Type III secretion system cytotoxic effector protein
v1.20647	K03980	Virulence factor, integral membrane protein
v1.18964	K03810	Virulence factor, oxidoreductase domain

The genome of *A. digitifera* also reveals homologues of genes that promote bacterial pathogenicity (Table 13), including virulence factors that are expressed and excreted by invading pathogens (bacteria, viruses, fungi and protozoa) to inhibit certain protective functions of the host. Such are the bacterial Type III cytotoxic effector protein and multiple Type IV Cag pathogenicity island proteins encoded in the coral genome. Many Gram-negative bacteria utilize Type III secretion proteins, which are regulated by quorum sensing, to deliver cytotoxic effector proteins into eukaryote host cells during infection. Cag (cytotoxin-associated) pathogenicity island (PAI) proteins are encoded by mobile genetic elements of the Type IV system secreting both proteins and large nucleoprotein complexes [366] that may be transferred between prokaryotes to enhance selected traits of virulence [367]. Our annotation reveals genes encoding six pathogenicity island proteins (Table 13) with similarity to the Cag PAI proteins of the human *Heliobacter pylori*, an infectious bacterium causing peptic ulcers that may lead to the development of stomach cancer. While many properties of Type III and IV secretion system proteins have been well characterized in bacteria, the functional purpose of homologous genes in *A. digitifera*, if expressed, are unknown.

The genome of *A. digitifera* contains genes of bacterial origin that encode the motility quorum-sensing regulator of the GCU-specific mRNA interferase toxin and acyl homoserine lactone synthesis used for the communication of quorum sensing between bacteria to enable the coordination of group behaviour based on collective population density. Apparent in our annotation (Table 13) is a wide array of microbial penicillin-binding proteins (PBPs) that have an affinity for β-lactam antibiotics that by binding to PBPs prevent bacteria from constructing a cell wall. There are genes also to enhance antibiotic resistance, including potential expression of a penicillinase repressor, a methicillin resistance protein and bleomycin hydrolase (cysteine peptidase). Additionally, isopenicillin-N synthase and an isopenicillin-N epimerase, both of which catalyse key steps in the biosynthesis of penicillin and cephalosporin antibiotics, are encoded in the coral genome. Taken as a whole, we demonstrate an extensive presence of ancient non-metazoan genes that are maintained in the genome of *A. digitifera*, as is reported in the genomes of *A. millepora* and the anemone *N. vectensis*[368]. Recent thought on genome evolution places these ancestral conserved domains as ‘orphan’ or ‘taxonomically restricted’ genes [352,369,370], rather than acquired later by horizontal gene transfer. There is, of course, little knowledge of how or when, if at all, these non-metazoan genes are expressed or even their function to mediate pathogenicity in the coral holobiont.

### Proteins of viral pathogenicity

Marine viruses were of minor interest until 1989, when it was realised that virus-like particles (VLPs) are the most abundant biological entities to occupy aquatic environments with variable numbers reaching ~10^8^ VLPs ml^-1^[371]. Typically, VLPs surpass the number of marine bacteria by an order of magnitude in coastal waters [372]; their diversity is extremely high and many are specific to the marine environment [373,374]. Significant VLP numbers are reported from the surrounding waters of oceanic coral reef atolls [375], in waters flowing across the reef substratum [376] and in samples taken within the close vicinity of coral colonies [377,378]. The viral load within the surface microlayer of scleractinian corals is enumerated as being 10^7^-10^8^ VLPs mL^-1^[379] and, based on VLP morphological diversity, is attributed to infecting various microbial hosts (bacteria, archaea, cyanobacteria, fungi and algae) residing within the coral mucus [380]. VLPs have been observed in the epidermal and gastrodermal tissues of corals and occasionally occur in the mesogloea [381]. Latent viruses were found to infect *Symbiodinium* isolated from several scleractinian corals [382-384] with a preponderance of eukaryotic algae-infecting phycodnaviruses suggested [385]. A wide range of bacteriophage and eukaryotic virus families have been identified within scleractinians using metagenomic analyses [207,386-388], with bacteriophages being by far the most abundant entities (Wood-Charlson EM, Weynberg KD, Suttle CA, Roux S, van Oppen MJH: Methodological biases in coral viromics*,* submitted).

The importance of the coral-virus interactome in bleaching and disease (reviewed in [185,389]) is founded on reports showing that VLP abundances are higher in the seawater immediately surrounding diseased compared to that of healthy corals [378], that latent viruses are induced by heat stress in symbiotic dinoflagellates of the sea anemone *Anemonia virdis*[382] and the coral *Pavona danai*[383], and that UV exposure induces a latent virus-like infection in cultured *Symbiodinium*[187]. Quantitative 454 pyrosequence analysis of the coral *Porites compressa* on exposure to reduced pH, elevated nutrients or thermal stress showed that the abundance of its viral consortia varied across treatments, but notably a novel herpes-like virus increased by up to 6 orders of magnitude on exposure to abiotic stress [387], although some caution may be warranted in assessing the reliability of such determinations [Wood-Charlson *et al.*, submitted]. Unexpectedly, the proteome of an endosymbiont-enriched fraction of the coral *Stylophora pistillata* showed a significant 114-fold increase in a viral replication protein on thermal bleaching [39], which is consistent with the finding of VLP induction in *P. compressa* by similar treatment [387].

General aspects of histocompatibility [390-393] and the genetic structure of innate immune receptors of the Cnidaria [363,394-401], including the immune response effected by coral disease and bleaching [364,402], have been examined extensively, hence further elaboration here is unnecessary. Instead, we focus on proteins that directly regulate the pathogenicity of coral-associated microbes and viruses. The *A. digitifera* genome encodes protein homologues having either putative antiviral and virus-promoting activities (Table 14). These homologues include the antiviral “superkiller” helicase SKI2 protein that acts by blocking viral mRNA translation [403] and, together with the superkiller proteins SKI3 (69 sequence alignments) and SKI8 of the exosome complex, function in a 3′-mRNA degradation pathway [404]. The coral genome encodes also three exoribonuclease (RNase) enzymes (XRN, XRN2 and RNB) with antiviral RNA-degrading properties [405,406]. Annotation of the coral genome reveals homologues to four interferon proteins (IFNB, IFNG, IFNW1 and IFNT1). Interferons are potent and selective antiviral cytokines [407], which are induced by viral infection or by sensing dsRNA, a by-product of viral replication, leading to the transcription of interferon-stimulated genes whose products have antiviral activities and others having antimicrobial, antiprolifera-tive/antitumor or immumomodulatory effects [408,409]. Included in the coral antivirus defence system are three members of the interferon regulatory transcription factor (IRF1, IRF2 and IRF8) family proteins. IRF1 and IRF2 are transcriptional activators of cytokines and other target genes [410]; IRF1 is known to trans-activate the tumor suppressor protein p53 [411] while IRF2 regulates post-transcriptional induction of NO synthase [412]. Conversely, IRF8 is an interferon consensus sequence-binding protein that is a negative (interference) regulator of enhancer elements common to interferon-inducible genes [413]. The coral genome additionally includes an interferon-stimulated 20 kDa protein (ISG20) RNase specific to deactivation of singled-stranded RNA viruses [414]. The coral genome encodes several interferon-inducible proteins, notably interferon gamma induced GTPase (IGTP) that accumulates in response to IFNB [415], the interferon-induced GTP-binding protein Mx1 that is a key element of host antiviral defence [416], the interferon-induced helicase C domain-containing protein1 (aka MDA-5), which is an immune receptor that senses viral dsRNA to activate the interferon antiviral-response cascade [417] and the interferon-induced transmembrane protein (IFITM1) that suppresses cell growth [418]. The coral genome encodes the interferon-gamma receptor 2 (IFNGR2) transmembrane protein that activates downstream signal transduction cascades that control cell proliferation and apoptosis [419]. Encoded also is a homologue of the human bone marrow stromal cell antigen 2 (BST2) that inhibits retrovirus infection by preventing VLP release from infected cells [420]. Additionally encoded is a mitochondrial antiviral-signalling protein (MAVS) that triggers the host immune response by activation of the nuclear transcription factor NF-kB and the interferon regulatory transcription factor IRF3 which coordinates the expression of type-1 interferons such as IFNB [421].

**Table 14:** **Regulatory and related proteins of viral pathogenicity in the predicted proteome of *A. digitifera***

**Gene sequence**	**KEGG Orthology**	**Encoded protein description**
v1.20647; v1.06188; v1.21287	K12599	Antiviral helicase SKI2
v1.18443 [+ 40 other sequence copies]	K12807	Baculoviral IAP repeat-containing protein 1 (BIRC1)
v1.06263 [+ 6 other sequence copies]	K04725	Baculoviral IAP repeat-containing protein 2/3/4 (BIRC2/3/4)
v1.14355	K08731	Baculoviral IAP repeat-containing protein 5 (BIRC5)
v1.04171 [+ 7 other sequence copies]	K10586	Baculoviral IAP repeat-containing protein 6 (BIRC6)
v1.12348; v1.01945; v1.16612	K06731	Bone marrow stromal cell antigen 2 (antiviral BST2)
v1.01539 [+ 7 other sequence copies]	K04012	Complement component receptor 2 (CR2)
v1.17305	K04462	Ecotropic virus integration site 1 protein (EVI1)
v1.1496 [+ 4 other sequence copies]	K12618	5′-3′ Exoribonuclease 1 (antiviral XRN1)
v1.22746; v1.19002; v1.12850; v1.21216	K12619	5′-3′ Exoribonuclease 2 (antiviral XRN2)
v1.09005	K01147	Exoribonuclease II (antiviral RNB)
v1.22793; v1.12978; v1.19008; v1.20838	K09239	HIV virus type I enhancer-binding protein (HIVEP)
v1.02776 [+ 7 other sequence copies]	K15046	Influenza virus NS1A-binding protein (NS1A-BP)
v1.09829; v1.13077	K05415	Interferon beta (IFNB)
v1.11946; v1.21512; v1.11221; v1.11927	K04687	Interferon gamma (IFNG)
v1.21512	K14140	Interferon gamma induced GTPase (ITGP)
v1.11946	K05133	Interferon gamma receptor 2 (IFNGR2)
v1.01539 [+ 4 other sequence copies]	K04012	Interferon-induced GTP-binding protein Mx1
v1.10782; v1.23797; v1.17119; v1.03221	K12647	Interferon-induced helicase C domain-containing protein 1
v1.06274; v1.15849; v1.05943	K06566	Interferon induced transmembrane protein (IFITM1)
v1.21327; v1.24081	K05440	Interferon, omega 1 (IFNW1)
v1.11817	K09444	Interferon regulatory factor 1 (IRF1)
v1.11816; v1.07639	K10153	Interferon regulatory factor 2 (IRF2)
v1.11421	K10155	Interferon regulatory factor 8 (IRF8)
v1.02158	K12579	Interferon-stimulated gene 20 kDa protein (ISG20)
v1.15947	K05442	Interferon tau-1 (IFNT1)
v1.22825; v1.08034; v1.08520	K05788	Integration host factor subunit beta (IHFB)
v1.14899	K08220	MFS transporter, FLVCR family virus subgroup C receptor
v1.04514; v1.04513; v1.16929	K12648	Mitochondrial antiviral-signalling protein (MAVS)
v1.17718; v1.08002; v1.08001; v1.22382	K06081	Poliovirus receptor-related protein 1 (PVRL1)
v1.21413; v1.06637	K06531	Poliovirus receptor-related protein 2 (PVRL2)
v1.11740; v1.21467; v1.11410; v1.17135	K06592	Poliovirus receptor-related protein 3 (PVRL3)
v1.15077	K06593	Poliovirus receptor-related protein 4 (PVRL4)
v1.04158 [+ 68 other sequence copies]	K12600	Superkiller protein 3 (antiviral SHI3)
v1.18238 [+ 4 other sequence copies]	K12601	Superkiller protein 8 (antiviral SHI8)

The coral genome encodes a full set of baculoviral IAP repeat-containing proteins BIRC 1-6 (Table 14). The IAP (inhibitor of apoptosis) family proteins were first identified secreted by baculovirus to protect infected cells from death in the progression of viral replication [422]. Expressed by most eukaryotic organisms (reviewed in [423]), their IAP function is presumably conserved in corals. The coral genome encodes a full set of poliovirus receptor-related proteins (PVRL1-4) of the immunoglobulin superfamily, which bind and transport herpesviruses at the cellular membrane in the establishment of latent infections (reviewed in [424]). Encoded also is a complement component (3d/Epstein Barr virus) receptor 2 (CR2) protein that binds to the Epstein-Barr virus *Herpes viridae* with antigenic activity for disease prevention [425]. Another encoded protein is a homologue of the human immunodeficiency virus type 1 (HIV-1) enhancer-binding protein (HIVEP; aka EBP1) that attaches to the HIV long terminal repeat (LTR) region to activate transcription via the HIV LTR [426]. Present in the coral genome is also a homologue of the influenza virus non-structural binding protein NS1A-BP that interacts with the NS1 virulence factor of the influenza A virus *Orthomyxoviridae* to interfere with NS1-inhibition of pre-mRNA splicing within the host nucleosome [427]. NS1A-BP inhibits NS1A-mediated disruption of the host immune response caused by restricting interferon production and the antiviral effects of IFN-induced proteins [428]. The genome of *A. digitifera* encodes an integration host factor subunit beta (IHFB), first discovered as a host factor for bacteriophage λ integration of mobile genetic elements, that in *E. coli* is involved in multiple processes of DNA replication, site-specific recombination and gene expression [429]. A homologue of the MFS transporter feline leukemia virus subgroup C receptor (FLVCR) cell surface protein is encoded in the coral genome, which in cats confers susceptibility to FeLV-C infection [430]. Encoded also is a viral integration site 1 (EVI1) that in humans is an oncogenic transcription factor, often activated by viral infection, to cause proliferation of invasive tumours [431]. Arguably, these homologue proteins typically expressed in such distantly related species may have similar relevance in viral interactions of the coral holobiome.

How these regulatory proteins and viral receptors interact and respond to viral infection in corals is yet to be realised. The absence of virion-specific sequences (e.g. for nucleic acid replication or capsid structure) suggests that proviral DNA is absent from the coral genome, or it may be an artefact of the limited number of marine viral sequences deposited in public databases. Discovery of viral activity through proteomics [39] may, therefore, suggest that viral proteins are synthesised from a lytic infection, but this requires confirmation.

### Toxins and venom

A review of protein sequences deposited in the UniProt database in October 2012 shows that there are 150 known cnidarian toxins. These toxins have diverse biological activities (neurotoxins, pore-forming cytolysins and venom phospholipases) used to capture prey and for protection against predators [432] that are best characterised in sea anemones (Actiniaria) with 141 sequences deposited [433,434]. The cytotoxin MCTx-1 isolated from the Net Fire Coral *Millepora dichotoma* is the only toxin from a coral deposited in Uniprot (accession number A8QZJ5). However, our initial examination of the predicted proteome of *A. digitifera* shows 18 proteins with similarity to bacterial toxins and associated regulatory proteins (Table 15). Unlike reports from proteomic examination of the coral *S. pistillata*[39] and nematocysts (stinging organelles) of the jellyfish *Olindias samba-quiensis*[435], *Tamoya haplonema*, *Chiropsalmus quadrumanus*, *Chrysaora lactea* (PF Long *et al*., pers comm), by sea anemones [434] and by the highly dangerous box jellyfish *Chironex fleckeri*[436,437], no venoms typical of higher animals were found in the *A. digitifera* genome. This was because our annotation was carried out using the KEGG database (release v58 [53]) to relate *A. digitifera* protein sequences to KEGG orthologues. The KEGG database is a collection of proteins from well characterised and ubiquitous biochemical pathways. Animal venoms, however, are highly specialised proteins for which this release of the KEGG database does not contain any described orthologues.

**Table 15:** **Proteins homologous to bacterial toxins in the predicted proteome of *A. digitifera***

**Gene sequence**	**KEGG Orthology**	**Encoded protein description**
v1.20214	K11029	Anthrax edema toxin adenylate cyclase (CyaA)
v1.17686	K10921	Cholera toxin transcriptional activator (ToxR)
v1.13017	K11020	Exotoxin A (ToxA)
v1.23507	K13655	HTH-type transcriptional regulator (MsqA) antitoxin for MqsR
v1.21184	K11009	Murine toxin (Ymt)
v1.04313	K11033	Non-hemolytic enterotoxin A (NheA)
v1.08011	K11034	Non-hemolytic enterotoxin B/C (NheBC)
v1.08255	K13651	Motility quorum-sensing regulator (MqsR) interferase toxin
v1.15986	K11059	Probable enterotoxin A (EntA)
v1.13046	K04392	Ras-related C3 botulinum toxin substrate 1 (Rac1)
v1.13966	K11007	Shiga toxin subunit B (StxB)
v1.23958	K11063	Toxin A/B (TcdAB)
v1.21174	K10930	Toxin co-regulated pilin (TCP)
v1.05802	K10961	Toxin co-regulated pilus biosynthesis protein I (TcpI)
v1.21783	K10964	Toxin co-regulated pilus biosynthesis protein S (TcpS)
v1.14688	K15126	Type III secretion system cytotoxic effector protein (BteA)
v1.05520	K11028	Vacuolating cytotoxin (VacA)
v1.06590	K10954	Zona occludens toxin (Zot)

KEGG orthology-based annotation of the *A. digitifera* genome reveals genes encoding protein homologues of 10 bacterial toxins, 7 regulatory toxin proteins and a botulinum protein substrate (Table 15). Of the 9 toxin homologues, one with similarity to anthrax edema factor (EF) adenylate cyclase (CyaA) is one of three proteins that comprise the anthrax toxin of *Bacillus anthracis*, the other two being a protective antigen (PA) and lethal factor (LF). Without the LF protein, anthrax CyaA has no known toxic effects in animals [438], although the EF protein does play an important role in disabling cellular functions vital for microbial host defences [439]. The *A. digitifera* genome encodes a secretion virulence factor exotoxin A-like protein produced by *Pseudomonas aeruginosa*, which for this bacterium affects local tissue damage, bacterial invasion and immunosuppression within their eukaryote host [440] with pathogenicity similar to that of the diphtheria toxin [441]. Another encoded protein is a murine-like toxin (Ymt) produced by the enterobacterium *Yersinia pestis*, which is the causative agent responsible for transmission of the notorious bubonic plague [442]. Additionally, two hemolytic enterotoxins similar to NheA and NheBC produced by *Bacillus cereus*[443], an enterotoxin (EntA) similar to that of *Staphylococcus aureus*[444], a Shiga-like enterotoxin (StxB) produced by *Shigella dysenteria*, the diarrhoea-causing toxin A/B (TcdAB) such as that secreted by *Clostridium difficile*[445], and a protein similar to the zonula occludens (tight junction) enterotoxin (Zot) secreted by *Vibrio cholera*[446] are encoded in the *A. digitifera* genome. Within the predicted proteome is also a homologue of the vacuolating cytotoxin (VacA) produced by *Helicobacter pylori* that colonises the gastric mucosa of the human stomach epithelium [447].

Although a direct homologue of the cholera toxin (CT) was not found encoded in the *A. digitifera* genome (Table 15), a protein similar to its transcriptional activator ToxR was. ToxR not only controls the expression of CT in *Vibrio cholera*[448], but also a co-regulated pilin (TcpA) protein that is under control of the ToxR regulon cascade [449]. Bacterial TcpA protein is assembled into toxin-coregulated pili that induce the transfer of DNA by horizontal exchange of genetic material during conjugation [450]. TcpA and two toxin co-regulated biosynthetic proteins (TcpI and Tcps) of the bacterial virulence-associated pilus appendage [451] are encoded in the coral genome. Entrained also are the motility quorum-sensing interference regulator MsqR and its transcriptional regulator MsqA that in *Eschericia coli* controls biofilm formation by inhibiting quorum-sensing motility, and together the MqsR/MqsA complex represses the lethal cold shock-like protein cspD gene [452] that on expression impairs DNA replication [453]. The *A. digitifera* genome likewise encodes a Type III secretion system T3SS cytotoxic effector (BteA) protein [454] that in Gram-negative invasive bacteria is translocated into host cells to suppress innate immunity to enhance virulence [455,456]. However, the ecophysiological significance of these toxigenic proteins and allied regulators, if indeed expressed by the coral genome, is unknown.

In addition to using the KEGG database, we undertook a BLAST search of the predicted proteome of *A. digitifera* against peptide sequences for all animal venoms using the annotated UniProtKB/Swiss-Prot Tox-Prot program [457]. This search revealed a large number of accession hits from the predicted proteome, although these are unlikely to be true multiple copies given that the genome sequence has yet to be completely assembled. However, just taking a single accession number from each annotation reveals a complex array of 83 toxins that represents the predicted venom of *A. digitifera* (Table 16); UniProt BLAST E-values are given in Additional file 1: Table S16b. These venoms are highly diverse and are significantly homologous to toxins from a wide variety of venomous marine and terrestrial creatures such as fish, reptiles, other cnidarians, cone-snails, stinging insects and even a venomous mammal (Shrew), covering the complete range of pharmacological properties known in venoms, including cytolytic, neurotoxic, haemotoxic, phospholipase, proteinase and proteinase inhibitor activities. Both the number of toxins predicted in the venom of *A. digitifera* and the degree of homology to such widely divergent phyla is remarkable. Accordingly, cnidarian venoms may possess unique biological properties that might generate new leads in the discovery of novel pharmacologically active drugs. Gene duplication followed by mutation and natural selection is widely held as the key mechanism whereby the large diversity of toxins found within a single venom could have evolved [458,459]. Conversely, primary mRNA splicing patterns have been shown to account for the diversity of metallopro-teinases in the pit viper *Bothrops neuwiedi*[460]. Variations in peptide processing have also been shown by proteomics and transcriptomics to explain how a limited set of genes transcripts could generate thousands of toxins in a single species of cone snail [461]. Despite these various processes that could account for the evolution of toxin diversity, it has never been demonstrated how gene duplications or variations in transcript or peptide processing could have radiated across the very different poisonous creatures found on Earth. Our data (Table 16) reveal that the predicted toxins of *A. digitifera* venom are orthologues to all of the most important superfamilies of peptide/protein venoms found in diverse taxa. We posit that the origins of toxins in the venoms of higher organisms may have arisen from deep eumetazoan innovations and that the molecular evolution of these venom super gene families can now be addressed taking an integrated venomics approach using Cnidaria such as the jellyfish as model systems [462].

**Table 16:** **UniProt-predicted homologues of animal venom proteins in the predicted proteome of *A. digitifera***

**Gene sequence**	**UniProt toxin accession**	**Animal with closest homology**
v1.01916 [+ 5 other sequence copies]	Q92035; Acetylcholinesterase	*Bungarus fasciatus* (Banded Krait)
v1.06761; v1.08075; v1.09840; v1.20323	Q9IAM1; Agkisacutacin (subunit anticoagulant protease)	*Deinagkistrodon acutus* (Sharp-nosed Viper)
v1.04809	A8QL52; L-Amino acid oxidase	*Bungarus fasciatus* (Banded Krait)
v1.06380	Q4JHE1; L-Amino acid oxidase	*Pseudechis australis* (Mulga Snake)
v1.10291	P81383; L-Amino acid oxidase	*Ophiophagus hannah* (King Cobra)
v1.14412	A6MFL0; L-Amino acid oxidase	*Demansia vestigiata* (Lesser Black Whipsnake)
v1.16469	P81383; L-Amino acid oxidase	*Ophiophagus hannah* (King Cobra)
v1.23477	P81382; L-Amino acid oxidase	*Calloselasma rhodostoma* (Malayan Pit Viper)
v1.16440	C5NSL2; Bandaporin (haemolysin)	*Anthopleura asiatica* (Sea Anemone)
v1.16571 [+ 10 other sequence copies]	Q76B45 ; Blarina toxin (vasoactive protease)	*Blarina brevicauda* (Northern Short-Tailed Shrew)
v1.06055 [+ 20 other sequence copies]	Q593B6; Coagulation factor V	*Pseudonaja textilis* (Eastern Brown Snake)
v1.07831; v1.10094 ; v1.20732	P14530; Coagulation factor IX	*Protobothrops flavoviridis* (Okinawa Habu Snake)
v1.01708 [+ 5 other sequence copies]	Q4QXT9; Coagulation factor X	*Tropidechis carinatus* (Rough-Scaled Snake)
v1.09601; v1.10410	Q93109; Equinatoxin-5 (cytolysin)	*Actinia equina* (Beadlet Anemone)
v1.06821	Q08169 ; Hyaluronidase	*Apis mellifera* (European Honey Bee)
v1.08924	I0CME7; Hyaluronidase, Conohyal-Cn1	*Conus consors* (Singed Cone)
v1.06189 [+ 112 other sequence copies]	Q9XZC0; α-Latrocrustotoxin Lt1a (neurotoxin)	*Latrodectus tredecimguttatus* (Mediterranean Black Widow Spider)
v1.02942 [+ 8 other sequence copies]	G0LXV8; α-Latrocrustotoxin Lh1a (neurotoxin)	*Latrodectus hasseltii* (Australian Redback Spider)
v1.00644 [+ 32 other sequence copies]	Q25338; Δ- Latroinsectotoxin Lt1a (neurotoxin)	*Latrodectus tredecimguttatus* (Mediterranean Black Widow Spider)
v1.07446	A7X3X3; Lectin, Lectoxin Enh4 (platelet binding)	*Enhydris polylepis* (Macleay’s Water Snake)
v1.20653	A7X3Y6; Lectin, Lectoxin Enh7 (platelet binding)	*Enhydris polylepis* (Macleay’s Water Snake)
v1.02561,v1.11493; v1.16681	A7X3Z4; Lectin, Lectoxin Lio1 (platelet binding)	*Liophis poecilogyrus* (Water Snake)
v1.13597; v1.08696; v1.10757; v1.20654	A7X3Z7; Lectin, Lectoxin Lio2 (platelet binding)	*Liophis poecilogyrus* (Water Snake)
v1.18386, v1.15479	A7X413; Lectin, Lectoxin Lio3 (platelet binding)	*Liophis poecilogyrus* (Water Snake)
v1.06094	A7X406; Lectin, Lectoxin Phi1 (platelet binding)	*Philodryas olfersii* (Green Cobra)
v1.06416; v1.16248; v1.23712	A7X3Z0; Lectin, Lectoxin Thr1 (platelet binding)	*Thrasops jacksonii* (Black Tree Snake)
v1.17681	Q6TPG9; Lectin, Mucrocetin (platelet binding)	*Protobothrops mucrosquamatus* (Brown Spotted Pit Viper)
v1.00077 [+ 14 other sequence copies]	Q66S03; Lectin, Nattectin (platelet binding)	*Thalassophryne nattereri* (Toad Fish)
v1.12241; v1.02332; v1.12298	Q71RQ1; Lectin, Stejaggregin-A (platelet binding)	*Trimeresurus stejnegeri* (Bamboo Viper)
v1.02245 [+ 19 other sequence copies]	A0FKN6; Metalloprotease, Astacin-like toxin	*Loxosceles intermedia* (Recluse Spider)
v1.03638; v1.14772	Q90391; Metalloprotease, Atrolysin	*Crotalus atrox* (Western Diamondback Rattlesnake)
v1.13106	D3TTC2; Metalloproteinase, Atragin	*Naja atra* (Chinese Cobra)
v1.11132	Q7T1T4; Metalloproteinase, BjussuMP-2	*Bothrops jararacussu* (Jararacussu Pit Viper)
v1.02168	O73795; Metalloproteinase, Disintegrin	*Gloydius brevicaudus* (Chinese Mamushi Snake)
v1.06910	Q7SZE0; Metalloproteinase, Disintegrin	*Gloydius saxatilis* (Rock Mamushi Snake)
v1.22282	P14530; Metalloproteinase, Disintegrin	*Protobothrops flavoviridis* (Okinawa Habu Snake)
v1.03804	Q2UXQ5; Metalloproteinase, EoVMP2	*Echis ocellatus* (West African Carpet Viper)
v1.02016	Q91511; Mucrofibrase-5, Hypotensive serine protease	*Protobothrops mucrosquamatus* (Brown Spotted Pit Viper)
v1.09026	Q7ZZN8; Natrin-2 (neurotoxin)	*Naja atra* (Chinese Cobra)
v1.04153; v1.04595; v1.12730; v1.04157	A0ZSK3; Neoverrucotoxin (haemolysin)	*Synanceia verrucosa* (Reef Stone Fish)
v1.12433 [+ 5 other sequence copies]	A2VBC4; Phospholipase A1	*Polybia paulista* (Neotropical Social Wasp)
v1.00019; v1.13757	Q06478; Phospholipase A1 1	*Dolichovespula maculata* (Bald-Faced Hornet)
v1.09322; v1.09961; v1.13629	P0CH47; Phospholipase A1, Magnifin	*Vespa magnifica* (Giant Hornet)
v1.03556	P53357; Phospholipase A1 2	*Dolichovespula maculata* (Bald-Faced Hornet)
v1.13015; v1.16921	D2X8K2; Phospholipase A2	*Condylactis gigantean* (Giant Caribbean Sea Anemone)
v1.18628	Q9TWL9; Phospholipase A2, Conodipine-M	*Conus magus* (Magical Cone)
v1.11796	Q9PUH9; Phospholipase A2, Acidic S9-53 F	*Austrelaps superbus* (Lowland Copperhead Snake)
v1.09883	Q8AXW7; Phospholipase A2, Basic	*Micrurus corallinus* (Painted Coral Snake)
v1.14874	Q90WA8; Phospholipase A2, Basic 2	*Bungarus fasciatus* (Banded Krait)
v1.11797	P20256; Phospholipase A2, Basic PA-12C	*Pseudechis australis* (Mulga Snake)
v1.07278 [+ 34 other sequence copies]	Q7SZN0; Prothrombin activator Pseutarin-C	*Pseudonaja textilis* (Eastern Brown Snake)
v1.11045	P83370; Prothrombin activator Hopsarin-D	*Hoplocephalus stephensii* (Stephen’s Branded Snake)
v1.04104 [+ 5 other sequence copies]	Q58L94; Prothrombin activator Notecarin D2	*Notechis scutatus* (Tiger Snake)
v1.00387 [+ 9 other sequence copies]	Q58L90; Prothrombin activator Omicarin C	*Oxyuranus microlepidotus* (Inland Taipan )
v1.02137 [+ 38 other sequence copies]	Q58L91; Prothrombin activator Omicarin C	*Oxyuranus scutellatus* (Coastal Taipan)
v1.00618 [+ 10 other sequence copies]	Q58L93; Prothrombin activator Porpharin D	*Pseudechis porphyriacus* (Red-Bellied Black Snake)
v1.09896	P81428; Prothrombin activator Trocarin D	*Tropidechis carinatus* (Rough-Scaled Snake)
v1.13726	A6MFK7; Prothrombin activator Vestarin D1	*Demansia vestigiata* (Lesser Black Whipsnake)
v1.02129; v1.05362; v1.20273	Q6T269; Protease inhibitor, Bitisilin-3 (neurotoxic)	*Bitis gabonica* (Gaboon Viper)
v1.06980; v1.09028	Q3SB05; Pseudechetoxin (neurotoxin)	*Pseudonaja textilis* (Eastern Brown Snake)
v1.21284 [+ 5 other sequence copies]	D8VNS7; Ryncolin-1 (haemostasis inhibitor)	*Cerberus rynchops* (Dog-Faced Water Snake)
v1.18895 [+ 20 other sequence copies]	D8VNS8; Ryncolin-2 (haemostasis inhibitor)	*Cerberus rynchops* (Dog-Faced Water Snake)
v1.14251; v1.10489; v1.14254	D8VNS9; Ryncolin-3 (haemostasis inhibitor)	*Cerberus rynchops* (Dog-Faced Water Snake)
v1.06759 [+ 7 other sequence copies]	D8VNT0; Ryncolin-4 (haemostasis inhinitor)	*Cerberus rynchops* (Dog-Faced Water Snake)
v1.01273	Q9YGN4; Salmorin toxin (haemostasis inhibitor)	*Gloydius brevicaudus* (Chinese Mamushi Snake)
v1.09855; v1.09856	B2DCR8; SE-Cephalotoxin	*Sepia esculenta* (Golden Cuttlefish)
v1.16247	O13060; Serine protease, 2A	*Trimeresurus gramineus* (Bamboo Viper)
v1.08397; v1.09733	Q9DF66; Serine protease, 3 (haemostasis inhibitor)	*Protobothrops jerdonii* (Jerdon’s Pit Viper)
v1.03275	Q9DG84; Serine protease, Serpentokallikrein-2 (haemostasis inhibitor)	*Protobothrops mucrosquamatus* (Brown Spotted Pit Viper)
v1.16638	Q7SYF1; Serine protease, Cerastocytin (platelet binding)	*Cerastes cerastes* (Saharan Horned Viper)
v1.22320	P0C5B4; Serine protease, Gloshedobin (platelet binding)	*Gloydius shedaoensis* (Shedao Pit Viper)
v1.15074 [+ 4 other sequence copies]	B2D0J4; Serine protease, Venom dipeptidyl peptidase 4	*Apis mellifera* (European Honey Bee)
v1.05361	B6RLX2; Serine protease inhibitor, TCI (neurotoxin)	*Ophiophagus hannah* (King Cobra)
v1.10994	B7S4N9; Serine protease inhibitor, Taicatoxin (neurotoxin)	*Oxyuranus scutellatus* (Coastal Taipan)
v1.11218; v1.23374	Q90WA0; Serine protease inhibitor, Textilinin-2 (thrombin inhibitor)	*Pseudonaja textilis* (Eastern Brown Snake)
v1.17856; v1.22256	Q8T3S7; Serine protease inhibitor, U1-aranetoxin-Av1a (neurotoxin)	*Araneus ventricosus* (Devil Spider)
v1.04154 [+ 4 other sequence copies]	Q98989; Stonustoxin (haemostasis inhibitor)	*Synanceia horrida* (Estuarine Stonefish)
v1.09427; v1.16619; v1.19446	Q76DT2; Toxin AvTX-60A (cytolysin)	*Actineria villosa* (Okinawan Sea Anemone)
v1.12311	Q9GV72; Toxin CrTX-A (haemolysin)	*Carybdea rastonii* (Jimble Jellyfish)
v1.07546 [+ 5 other sequence copies]	P58911; Toxin PsTX-60 (haemolysin)	*Phyllodiscus semoni* (Night Anemone)
v1.11270; v1.14265	E2IYB3; Veficolin-1 (complement activator)	*Varanus komodoensis* (Komodo Dragon)
v1.02115	Q98993; Verrucotoxin (cytolysin)	*Synanceia verrucosa* (Reef Stonefish)

### Detoxification proteins of the chemical defensome

There have been considerable advancements made to better understand the effects of pollution on coral reef habitats. The three main categories of environmental pollutants from anthropogenic sources are nutrient enrichment (eutrophication), hydrocarbon pollution and heavy metal contamination. Eutrophication from terrestrial inputs are a significant threat to coral reefs stemming from the discharge of treated sewage, the runoff of agricultural fertilizers (plus herbicides and pesticides), and by sedimentation caused by the erosion of organic-rich soils [463]. Notwithstanding that eutrophication can shift coral reef communities towards macroalgae domination [19], nitrogen and phosphorus enrichment can diminish coral growth and affect the photosynthetic performance of their algal symbionts [464]. Nutrient enhancement alters multiple pathways of primary metabolism that in coral is complicated by the photosynthetic demands of its symbiotic partners. While corals respond to hypertrophic levels of nutrients by activating general stress-response proteins [465], there are no specific proteins known to mitigate the cellular effects of nutrient enrichment on corals *per se*, and we have not attempted to identify such in this study.

Gene families and their regulators that defend against chemical stressors comprise the chemical defensome encoding a network of detoxifying proteins that allows an organism to sense, transform and eliminate potentially toxic endogenous metabolites and xenobiotic contaminants [466]. Expressed proteins of the chemical defensome include the biotransformation cytochrome P450 (CYP) family of enzymes, conjugating enzymes, efflux transporters, heavy metal membrane pump exporters and their transcriptional activators. Annotation of the genome of *A. digitifera* reveals multiple genes encoding 20 hemoproteins belonging to the Phase II cytochrome P450 superfamily of monooxidase enzymes that catalyse the oxidation of diverse organic substances (Table 17). The substrates of CYP enzymes include intermediates of lipid metabolism and sterol/steroid biosynthesis, and include the detoxification of exogenous xenobiotics. Of significance are the CYP1A-type (aryl hydrocarbon hydroxylase) enzymes that have been studied widely in the hepatic response of fishes to polycyclic aromatic hydrocarbon (PAH) contamination (from crude or fuel oil) and exposure to polychlorinated biphenyl and dibenzodioxin toxicants (reviewed in [467]). CYP450 activity has been detected in the corals *Favia fragum*[468], *Siderastrea siderea*[469], *Montastraea faveolata*[470] and *Pocillopora damicornis*, [471]. Furthermore, CYP encoding sequences have been extracted from the genome of *N. vectensis*[472] and the transcriptome of *A. millepora*[29]. As well as providing chemical defence, mixed-function CYPs perform multiple endogenous tasks that are often taxon-specific. Hence, the orthology and substrate specificity of coral CYP enzymes cannot be predicted solely on homology to CYPs of known function assigned to higher metazoans. Similar to the function of CPY enzymes, there are genes encoding p-hydroxybenzoate 3-monooxygenase, an oxidoreductase catalyzing aryl oxidation and the soluble and microsomal forms of epoxide hydrolase that converts epoxides, formed by the degradation of aromatic compounds, to trans-diols that by conjugation are readily excreted. Conjugating enzymes to eliminate hydroxylated substrates are the detoxifying UDP-glucuronosyltransferase and sulfotransferase families of enzymes. Estrone sulfotransferase is significant for inactivation of exogenous (contraceptive) estrogens [473] and similar endocrine-disruptive contaminants released from treated wastewater [474]; their occurrence in marine waters are known to disrupt the reproduction and development of fish [475] and corals [476]. Glutathione S-transferase (GST) enzymes catalyse the addition of reduced glutathione to the reactive sites of electrophilic toxins [477]. Surprisingly, only two isoforms of GST were detected in the *A. digitifera* genome (Table 17), whereas 18 distinct GST-encoding genes (6 classes + 1 fungal-type) were classified from genome sequences of *N. vectensis*[472]. This unexpected genome reduction of GST elaboration in *A. digitifera* begs further examination.

**Table 17:** **Proteins of the chemical defensome in the predicted proteome of *A. digitifera***

		
**Gene sequence**	**KEGG Orthology**	**Encoded protein description**
v1.06127; v1.06128	K01015	Alcohol sulfotransferase
v1.09267	K00537	Arsenate reductase
v1.24496; v1.24495; v1.03953	K03893	Arsenical pump membrane protein
v1.10691	K07755	Arsenite methyltransferase
v1.20443	K11811	Arsenical resistance protein ArsH
v1.14972	K01551	Arsenite-transporting ATPase
v1.17644; v1.00480; v1.08150; v1.22865	K01014	Aryl sulfotransferase
v1.21535; v1.11835; v1.02456	K01534	Cd^2+/^Zn^2+^-exporting ATPase
v1.03485; v1.21926; v1.05686	K01533	Cu^2+^-exporting ATPase
v1.22646 [+ 8 other sequence copies]	K07408	Cytochrome P450, family 1, subfamily A, polypeptide 1
v1.01284	K07421	Cytochrome P450, family 2, subfamily T
v1.10544; v1.02314, v1.17490	K07422	Cytochrome P450, family 2, subfamily U
v1.23039 [+ 13 other sequence copies]	K07422	Cytochrome P450, family 3, subfamily A
v1.07750	K07425	Cytochrome P450, family 4, subfamily A
v1.22798; v1.23000	K07426	Cytochrome P450, family 4, subfamily B
v1.02020 [+ 4 other sequence copies]	K07427	Cytochrome P450, family 4, subfamily V
v1.19495	K07428	Cytochrome P450, family 4, subfamily X
v1.15382	K15002	Cytochrome P450, family 6
v1.16427	K07430	Cytochrome P450, family 7, subfamily B
v1.17631	K00498	Cytochrome P450, family 11, subfamily A
v1.08074 [+ 4 other sequence copies]	K15004	Cytochrome P450, family 12
v1.02478 [+ 5 other sequence copies]	K00512	Cytochrome P450, family 17, subfamily A
v1.06713	K07435	Cytochrome P450, family 20, subfamily A
v1.22414 [+ 5 other sequence copies]	K07436	Cytochrome P450, family 24, subfamily A
v1.20153	K12665	Cytochrome P450, family 26, subfamily C
v1.08074 [+ 6 other sequence copies]	K00488	Cytochrome P450, family 27, subfamily A
v1.06537	K07439	Cytochrome P450, family 39, subfamily A
v1.22302 [+ 5 other sequence copies]	K07440	Cytochrome P450, family 46, subfamily A
v1.16335	K09832	Cytochrome P450, family 710, subfamily A
v1.18439; v1.02594; v1.02593	K01016	Estrone sulfotransferase
v1.07758 [+ 5 other sequence copies]	K00699	Glucuronosyltransferase
v1.00764	K13299	Glutathione S-transferase kappa 1
v1.17188	K00799	Glutathione S-transferase
v1.04140	K07239	Heavy-metal exporter, HME family
v1.10181	K00481	p-Hydroxybenzoate 3-monooxygenase
v1.16748; v1.07471	K08365	MerR family transcriptional regulator, mercuric resistance
v1.04382; v1.24424	K13638	MerR family transcriptional regulator, Zn(II)-responsive
v1.12760	K08363	Mercuric ion transport protein
v1.04179; v1.01891; v1.00145	K03284	Metal ion transporter, MIT family
v1.21500 [+ 5 other sequence copies]	K01253	Microsomal epoxide hydrolase
v1.08005	K08970	Nickel/cobalt exporter
v1.03484	K08364	Periplasmic mercuric ion binding protein
v1.05406	K07245	Putative copper resistance protein D
v1.14635	K08726	Soluble epoxide hydrolase
v1.01929; v1.19296	K05794	Tellurite resistance protein TerC
v1.10880; v1.15709; v1.12348	K07803	Zinc resistance-associated protein

Many toxicological studies on the effects of pollution on cnidarian fitness have focused on their response to heavy metal contamination, including copper, cadmium, mercury and zinc [478,479]. In scleractinian corals the uptake and toxic effects of copper [480-483], cadmium [482] and mercury [484,485] have been studied at the metabolic level with specific studies to examine the effects of heavy metal toxicity on coral fertilisation [486-488], settlement [487], metamorphosis [486] and in coral bleaching [489]. Yet, the identification of molecular markers to monitor the response of Cnidaria to sub-lethal levels of heavy metal exposure has been elusive [490]. We were delighted to uncover in our annotation a wide range of genes to express metal-specific (arsenic, copper, mercury, nickel/cobalt and tellurium) resistance, transportation and membrane pump exporting proteins that, together with non-specific heavy metal ion export proteins (Table 17), might prove useful for monitoring the environmental response of *A. digitifera* to heavy metal contamination. Included in the heavy metal defensome are the Mer-family of transcriptional regulators of Hg- and Zn-resistance proteins and a periplasmic ion-binding protein attributed to the Hg detoxification system of bacteria [491]. Enzymes specific for arsenic detoxification are an arsenate oxidoreductase for conversion of arsenate to arsenite [492] and arsenite methyltransferase for conversion of arsenite to the less toxic dimethylarsenite that is amenable to excretion [493]. Such processes may enhance the resilience of corals exposed to natural [494] and site-affected [495] levels of arsenic contamination. In contrast, there were no (organo)cyanide detoxification genes apparent in the *A. digitifera* genome, but one sequence (v1.01601; K10814) encodes for hydrogen cyanide synthase of unknown metabolic purpose (data not tabulated). Ancillary evidence suggests that the expression of HCN synthase could be linked to quorum sensing [496] for regulating microbial densities of the coral holobiont community.

### Epigenetic and DNA-remodelling proteins

In all Kingdoms of life, DNA methylation and chromatin remodelling is pivotal to the regulation of gene transcription independent of underlying allelic variation. One such process mediated by epigenetic changes in eukaryotic biology is the all-important cellular differentiation during morphogenetic development. Epigenetic modifications cause the activation, regulation or silencing of certain genes without changing the basic DNA code. Changes in epigenetic regulation can persist during cell division and across multiple generations [497]. In addition, cytosine methylation may be associated with a higher mutation rate, because deamination of the methylated base produces thymine resulting in C/T mutations, which on reproduction may be transmitted by the germline to subsequent generations in selective processes of evolution [498]. On the other hand, environmentally induced destabilisation of the epigenome can produce epigenetic gene variants (epialleles) that activate transcription and mobilization of DNA transposable elements, which may subsequently lead to stable heritable traits of environmental adaptation, as does occur by genetic imprinting in plants [499]. Transposition has thus the potential to direct increased frequencies of permanent genetic mutations for selective adaptation.

One way by which genes are regulated at the epigenome is through the remodelling of the chromatin histone-DNA complex (the nucleosome), which by post-translational modification changes the template structure of DNA associated histone proteins. These modifications are affected by histone-lysine (and histone-arginine) N-methyltransferase enzymes (Table 18) by which these proteins may be further modified by acetylation, ADP-ribosylation, ubiquination, and phosphorylation (annotation not tabulated). The methylation pattern of histone lysine residues is highly predictive of the gene expression states of transcriptional activation and repression [500]. Necessary epigenomic reprogramming of histone modification at different stages of cell development is affected by the activation of histone and lysine-specific demethylase enzymes (Table 18). Determinants for recognition of the histone code are being revealed by a growing body of experimental data providing valuable information on the molecular tractability of binding sites involved in epigenetic signalling [501], which will enhance further insight to epigenetic function.

**Table 18:** **Epigenetic and DNA-remodelling proteins in the predicted proteome of *A. digitifera***

		
**Gene sequence**	**KEGG Orthology**	**Encoded protein description**
v1.04426; v1.02042	K02528	16S rRNA (adenine1518-N6/1519-N6)-dimethyltransferase
v1.22358; v1.00249	K14191	18S rRNA (adenine1779-N6/1780-N6)-dimethyltransferase
v1.19400; v1.04238	K00561	23S rRNA (adenine2085-N6)-dimethyltransferase
v1.05107; v1.05242	K01488	Adenosine deaminase
v1.04152; v1.09790	K14857	AdoMet-dependent rRNA methyltransferase SPB1
v1.00197	K13530	AraC family transcriptional regulator DNA methyltransferase
v1.12967; v1.19789; v1.07763	K14589	Cap-specific mRNA (nucleoside-2′-O-)-methyltransferase 1
v1.24281	K01489	Cytidine deaminase
v1.16211; v1.14952; v1.01094; v1.06983	K00558	DNA (cytosine-5-)-methyltransferase
v1.19683; v1.05688; v1.04223	K11324	DNA methyltransferase 1-associated protein 1
v1.14033; v1.19860; v1.19081; v1.04188	K11420	Euchromatic histone-lysine N-methyltransferase
v1.02068	K01487	Guanine deaminase
v1.02920	K05931	Histone-arginine methyltransferase CARM1
v1.17589 [+ 7 other sequence copies]	K11446	Histone demethylase JARID1
v1.07640	K06101	Histone-lysine N-methyltransferase ASH1L
v1.13515; v1.18577; v1.20187; v1.19182	K09186	Histone-lysine N-methyltransferase MLL1
v1.08381	K09187	Histone-lysine N-methyltransferase MLL2
v1.24258; v1.19182	K09188	Histone-lysine N-methyltransferase MLL3
v1.07992; v1.10302; v1.13829	K09189	Histone-lysine N-methyltransferase MLL5
v1.06939; v1.15255; v1.15254	K11424	Histone-lysine N-methyltransferase NSD1/2
v1.05552	K11422	Histone-lysine N-methyltransferase SETD1
v1.07744	K11423	Histone-lysine N-methyltransferase SETD2
v1.03190	K11431	Histone-lysine N-methyltransferase SETD7
v1.21867	K11428	Histone-lysine N-methyltransferase SETD8
v1.18700 [+ 8 other sequence copies]	K11421	Histone-lysine N-methyltransferase SETDB
v1.07557; v1.11409	K11419	Histone-lysine N-methyltransferase SUV39H
v1.24733; v1.13497	K11429	Histone-lysine N-methyltransferase SUV420H
v1.15405; v1.10291; v1.17601; v1.02845; v1.08629	K11450	Lysine-specific histone demethylase 1
v1.23155; v1.09394; v1.17624; v1.05370	K14835	Ribosomal RNA methyltransferase Nop2
v1.18460 [+ 6 other sequence copies]	K03500	Ribosomal RNA small subunit methyltransferase B
v1.07407; v1.03110	K08316	Ribosomal RNA small subunit methyltransferase D
v1.12193	K02427	Ribosomal RNA large subunit methyltransferase E
v1.11499	K11392	Ribosomal RNA small subunit methyltransferase F
v1.16053; v1.12676	K03437	RNA methyltransferase, TrmH family
v1.12453; v1.05459	K13097	Methylcytosine dioxygenase
v1.07692	K07451	5-Methylcytosine-specific restriction enzyme A
v1.21815; v1.17113	K00565	mRNA (guanine-N7-)-methyltransferase
v1.06363; v1.03360; v1.21218	K05925	mRNA (2′-O-methyladenosine-N6-)-methyltransferase
v1.09661	K07442	tRNA (adenine-N1-)-methyltransferase catalytic subunit
v1.08094; v1.04036; v1.18614	K03256	tRNA (adenine-N(1)-)-methyltransferase non-catalytic subunit
v1.11456; v1.00738; v1.04577	K03439	tRNA (guanine-N7-)-methyltransferase
v1.08042	K14864	tRNA methyltransferase
v1.20501	K00557	tRNA (uracil-5-)-methyltransferase
v1.15147	K14964	Set1/Ash2 histone methyltransferase subunit ASH2
v1.08925	K00571	Site-specific DNA-methyltransferase (adenine-specific)

Direct epigenetic modification of DNA (or mRNA) occurs by methylation of cytosine, and to a lesser extent adenosine and guanine, by nucleobase-specific DNA methyltranferases (Table 18) to give 5-methylcytosine (5-meC), 3-methyladenosine (3-meA) and 3-methylguanine (3-meG) nucleotides, respectively. The principal modification product, 5-methylcytosine behaves much like regular cytosine by pairing with guanine, but in areas of high cytosine methylation, genome transcription is strongly repressed (reviewed in [502]), together with the repression of other chromatin-dependent processes, including the incorporation of transposable elements [503]. Alteration in the methylation status of the entire genome, individual chromosomes or at specific gene sites is essential for normal cellular function, but processes for reprogramming methylated DNA at different stages of cell development, unlike the reversal of histone modifications, is poorly defined [504]. While there are abundant enzymes to repair DNA damage caused by spurious N-alkylation, direct nucleotide C-demethylation (via the hypothetical “DNA demethylase” [505]) is thermodynamically infeasible. Instead, removal of epigenetic C-methylated nucleobases occurs by several base-repair pathways involving DNA excision or mismatch repair enzymes. The genome of *A. digitifera* encodes expression of a specific DNA glycosylase enzyme [506] for excision of 3-meA, but there are no such enzymes encoded for the excision of 5-meC and 3-meG, although there is encoded a 5-methylcytosine-specific restriction enzyme. Another pathway for DNA demethylation requires base-specific deamination by the AID/Apobec family of deaminase enzymes that, for example, converts 5-meC to thymine that is replaced subsequently by cytosine by C/T mismatch repair enzymes. These methylated nucleobases are recognized for deamination by the cytosine, adenosine and guanine deaminase enzymes [507] that are encoded in the *A. digitifera* genome, and their deaminated bases are subsequently removed by DNA mismatch repair enzymes. Additionally, the genome of *A. digitifera* encodes a methlycytosine dioxygenase enzyme that converts 5-methylcytosine to 5-hydroxymethycytosine (5-hmC), which is recognized for removal by the base excision repair pathway [508] or via its 5-hmC deaminated intermediate [507]. Combined, these DNA demethylation pathways are able to remodel epigenetic modifications at different stages of cell development.

Most current knowledge on DNA and protein methylation comes from studies of mammals and plants, while our understanding of the extent and roles of DNA methylation in invertebrates, marine invertebrates in particular, is still limited [509]. Little is known about the epigenetic potential of corals to acclimatize and adapt to the thermal and synergistic stressors that cause wide-spread coral “bleaching” [510]. Yet, given that acclimatization occurs via the generation of epiallele variants that can in some instances lead to stable heritable traits of environmental adaptation, there is growing interest in the prospect that epigenetic modifications in corals or their algal symbionts [511] may drive adaptation to defend against the damaging threat imposed by rising temperatures from global climate change. It is anticipated that this field of study will rapidly accelerate with the need to better understand epigenetic processes that may contribute to the persistence of coral reefs.

## Conclusions

We offer ZoophyteBase as an unprecedented foundation to interrogate the molecular structure of the predicted *A. digitifera* proteome. Some key findings include proteins with relevance to host-symbiont function, dysfunction and recovery including those that direct vacuolar trafficking and proteins linking symbiont photosynthesis to coral calcification. An extensive catalogue of mammalian-like proteins essential to neural function and venoms related to distant animal phyla suggests their origins lie deep in early eumetazoan evolution. Homologues of prokaryotic genes that have not been described previously in any eukaryote genome such as flagella proteins, proteins essential for nitrogen fixation and photosynthesis point towards lateral gene transfer, perhaps mediated by viruses, that may lead to “shared” metabolic adaptations of symbiosis, and provide corals with limited ability for gene-encoded adaptation to a changing global environment. It is anticipated that understanding how the genome of a coral hosts interacts with that of its vast array of symbionts, and how it may regulate its metabolic quotient, for example through biochemical or epigenetic modification, will rapidly accelerate our ability to predict the fate of coral reefs.

## Availability and requirements

ZoophyteBase was constructed using the Metagenome/Genome Annotated Sequence Natural Language Search Engine (*MEGGASENSE*). This is a general system for the annotation of sequence collections and presentation of the results in a database that can be searched using biologically intuitive search terms. In this implementation, the predicted proteome of *A. digitifera* (genome assembly v. 1.0 [48]) was used as the source of protein sequences. The annotation was carried out using the KEGG database (release v58 [51]) to relate *A. digitifera* protein sequences to KEGG orthologues. The homologous protein sequences were used to construct hidden Markov model (HMM) profiles using the HMMER3 package [49]. The predicted proteome sequences of *A. digitifera* were searched with the HMM profiles to link proteins to appropriate KEGG orthologues [50,512]. A web interface was developed with various tools. The search platform Lucene/Solr [52] was used to implement natural language searches. Protein sequences provided by the user can be used for BLAST [50] searches against the coral proteome. Selected sequences of the coral proteome can be analysed with third party software (e.g. [53]) to interrogate conserved domains. ZoophyteBase is deployed using Apache-Tomcat (version 7.0.28 for Linux ×64 [513]) on the Ubuntu Linux server of the Section of Bioinformatics at the Faculty of Food Technology and Biotechnology, University of Zagreb, Croatia and is accessible at our published web address [47].

## Competing interests

The authors declare no competing interests exist.

## Authors’ contributions

WCD and PFL conceived the study, and participated in its design, coordination and drafted the manuscript. AS carried out the annotation. DB, JD, JZ and RG participated in the database design and testing. WCD, MJHvO, AS and PFL performed data analysis. DH and JC participated in and coordinated the annotation, database design and testing. All authors have read and approved the final manuscript.

## Supplementary Material

Additional file 1: Table S16b Predicted (UniProt) homologues of animal toxins encoded in the genome of *A. digitifera*. Click here for file

## References

[B1] Freudenthal HD *Symbiodinium* gen. nov. and *Symbiodinium microadriaticum* sp. nov., a zooxanthella: taxonomy, life cycle, and morphology J Eukaryot Microbiol 1962 9 45 52 10.1111/j.1550-7408.1962.tb02579.x

[B2] Muscatine L Dubinsky Z The role of symbiotic algae in carbon and energy flux in reef corals Coral Reefs: Ecosystems of the World 1990 Amsterdam: Elsevier 75 84

[B3] Yellowlees D Rees TAV Leggat W Metabolic interactions between algal symbionts and invertebrate hosts Plant Cell Environ 2008 31 679 694 10.1111/j.1365-3040.2008.01802.x 18315536

[B4] Johannes RE Wiebe WJ Crossland CJ Rimmer DW Smith SV Latitudinal limits of coral reef growth Mar Ecol Prog Ser 1983 11 105 111

[B5] Spalding MD Grenfell AM New estimates of global and regional coral reef areas Coral Reefs 1997 16 225 230 10.1007/s003380050078

[B6] Hatcher BG Coral reef primary productivity. A hierarchy of pattern and process Trends Ecol Evol 1990 5 149 155 10.1016/0169-5347(90)90221-X 21232343

[B7] Reaka-Kudla ML Reaka-Kudla ML, Wilson DE, Wilson EO The global biodiversity of coral reefs: A comparison with rainforests Biodiversity II. Understanding and Protecting Our Natural Resources 1997 Washington DC: Joseph Henry/National Academy Press 83 108

[B8] Davy SK Allemand D Weis VM Cell biology of cnidarians-dinoflagellate symbiosis Microbiol Mol Biol Rev 2012 76 229 261 10.1128/MMBR.05014-11 22688813 PMC3372257

[B9] Bellwood DR Hughes TP Folke C Nyström M Confronting the coral reef crisis Nature 2004 429 827 833 10.1038/nature02691 15215854

[B10] Hoegh-Guldberg O Climate change, coral bleaching and the future of the world’s coral reefs Mar Freshwat Res 1999 50 839 866 10.1071/MF99078

[B11] Hoegh-Guldberg O Mumby PJ Hooten AJ Steneck RS Greenfield P Gomez E Harvell CD Sale PF Edwards AJ Caldeira K Knowlton N Eakin CM Iglesias-Prieto R Muthiga N Bradbury RH Dubi A Hatziolos ME Coral reefs under rapid climate change and ocean acidification Science 2007 318 1737 1742 10.1126/science.1152509 18079392

[B12] Porter JW Dustan P Jaap WC Patterson KL Kosmynin V Meier OW Patterson ME Parsons M Patterns of spread of coral disease in the Florida Keys Hydrobiologia 2001 460 1 24 10.1023/A:1013177617800

[B13] Bruno JF Selig ER Casey KS Page CA Willis BL Harvell CD Sweatman H Melendy AM Thermal stress and coral cover as drivers of coral disease outbreaks PLoS Biol 2007 5 e124 10.1371/journal.pbio.0050124 17488183 PMC1865563

[B14] Brandt ME McManus JW Disease incidence is related to bleaching extent in reef-building corals Ecology 2009 90 2859 2867 10.1890/08-0445.1 19886494

[B15] Devlin MJ Brodie J Terrestrial discharge into the Great Barrier Reef Lagoon: nutrient behaviour in coastal waters Mar Pollut Bull 2005 51 9 22 10.1016/j.marpolbul.2004.10.037 15757704

[B16] Fabricius KE Effects of terrestrial runoff on the ecology of corals and coral reefs: review and synthesis Mar Pollut Bull 2005 50 125 146 10.1016/j.marpolbul.2004.11.028 15737355

[B17] Orr JC Fabry VJ Aumont O Bopp L Doney SC Feely RA Gnanadesikan A Gruber N Ishida A Joos F Key RM Lindsay K Maier-Reimer E Matear R Monfray P Mouchet A Najjar RG Plattner GK Rodgers KB Sabine CL Sarmiento JL Schlitzer R Slater RD Totterdell IJ Weirig MF Yamanaka Y Yool A Anthropogenic ocean acidification over the twenty-first century and its impact on calcifying organisms Nature 2005 437 681 686 10.1038/nature04095 16193043

[B18] Anthony KRN Kline DI Diaz-Pulido G Dove S Hoegh-Guldberg O Ocean acidification causes bleaching and productivity loss in coral reef builders Proc Natl Acad Sci U S A 2008 105 17442 17446 10.1073/pnas.0804478105 18988740 PMC2580748

[B19] De’ath G Fabricius K Water quality as a regional driver of coral biodiversity and macroalgae on the Great Barrier Reef Ecol Appl 2010 20 840 850 10.1890/08-2023.1 20437968

[B20] Carpenter KE Abrar M Aeby G Aronson RB Banks S Bruckner A Chiriboga A Cortés J Delbeek JC Devantier L Edgar GJ Edwards AJ Fenner D Guzmán HM Hoeksema BW Hodgson G Johan O Licuanan WY Livingstone SR Lovell ER Moore JA Obura DO Ochavillo D Polidoro BA Precht WF Quibilan MC Reboton C Richards ZT Rogers AD Sanciangco J One third of reef-building corals face elevated extinction risk from climate change and local impacts Science 2008 321 560 563 10.1126/science.1159196 18653892

[B21] Pendleton LH Valuing coral reef protection Ocean Coast Manage 1995 26 119 131 10.1016/0964-5691(95)00007-O

[B22] Halpern BS Walbridge S Selkoe KA Kappel CV Micheli F D’Agrosa C Bruno JF Casey KS Ebert C Fox HE Fujita R Heinemann D Lenihan HS Madin EM Perry MT Selig ER Spalding M Steneck R Watson R A global map of human impact on marine ecosystems Science 2008 319 948 952 10.1126/science.1149345 18276889

[B23] Hughes TP Baird AH Bellwood DR Card M Connolly SR Folke C Grosberg R Hoegh-Guldberg O Jackson JB Kleypas J Lough JM Marshall P Nyström M Palumbi SR Pandolfi JM Rosen B Roughgarden J Climate change, human impacts, and the resilience of coral reefs Science 2003 301 929 933 10.1126/science.1085046 12920289

[B24] Evans TG Hofmann GE Defining the limits of physiological plasticity: how gene expression can assess and predict the consequences of ocean change Philos Trans R. Soc B-Biol Sci 2012 367 1733 1745 10.1098/rstb.2012.0019 PMC3350660 22566679

[B25] Weis VM The susceptibility and resilience of corals to thermal stress: adaptation, acclimatization or both? Mol Ecol 2010 19 1515 1517 10.1111/j.1365-294X.2010.04575.x 20456235

[B26] Edge SE Morgan MB Gleason DF Snell TW Development of a coral cDNA array to examine gene expression profiles in *Montastraea faveolata* exposed to environmental stress Mar Pollut Bull 2005 51 507 523 10.1016/j.marpolbul.2005.07.007 16115654

[B27] Grasso LC Maindonald J Rudd S Hayward DC Saint R Miller DJ Ball EE Microarray analysis identifies candidate genes for key roles in coral development BMC Genomics 2008 9 540 10.1186/1471-2164-9-540 19014561 PMC2629781

[B28] Bay LK Ulstrup KE Nielsen HB Jarmer H Goffard N Willis BL Miller DJ van Oppen MJ Microarray analysis reveals transcriptional plasticity in the reef building coral *Acropora millepora* Mol Ecol 2009 18 3062 3075 10.1111/j.1365-294X.2009.04257.x 19538339

[B29] Meyer E Aglyamova GV Wang S Buchanan-Carter J Abrego D Colbourne JK Willis BL Matz MV Sequencing and de novo analysis of a coral larval transcriptome using 454 GSFlx BMC Genomics 2009 10 219 10.1186/1471-2164-10-219 19435504 PMC2689275

[B30] DeSalvo MK Sunagawa S Voolstra CR Medina M Transcriptomic responses to heat stress and bleaching in the elkhorn coral *Acropora palmata* Mar Ecol Prog Ser 2010 402 97 113

[B31] Portune KJ Voolstra CR Medina M Szmant AM Development and heat stress induced transcriptomic changes during embryogenesis of the scleractinian coral *Acropora palmata* Mar Genom 2010 3 51 62 10.1016/j.margen.2010.03.002 21798197

[B32] Souter P Bay LK Andreakis N Császár N Seneca FO van Oppen MJ A multilocus, temperature stress-related gene expression profile in *Acropora millepora*, a dominant reef-building coral Mol Ecol Resour 2011 11 328 334 10.1111/j.1755-0998.2010.02923.x 21429140

[B33] Ladner JT Barshis DJ Palumbi SR **Protein evolution in two co-occurring types of Symbiodinium: an exploration into the genetic basis of thermal tolerance in*****Symbiodinium*****clade D.** BMC Evol Biol 2012 12 217 10.1186/1471-2148-12-217 23145489 PMC3740780

[B34] Barshis DJ Ladner JT Oliver TA Seneca FO Traylor-Knowles N Palumbi SR Genomic basis for coral resilience to climate change Proc Natl Acad Sci USA 2013 110 1387 1392 10.1073/pnas.1210224110 23297204 PMC3557039

[B35] Granados-Cifuentes C Bellantuono AJ Ridgway T Hoegh-Guldberg O Rodriguez-Lanetty M **High natural gene expression variation in the reef-building coral*****Acropora millepora***: **potential for acclimative and adaptive plasticity** BMC Genomics 2013 14 228 10.1186/1471-2164-14-228 23565725 PMC3630057

[B36] Traylor-Knowles N Granger BR Lubinski TJ Parikh JR Garamszegi S Xia Y Marto JA Kaufman L Finnerty JR Production of a reference transcriptome and transcriptomic database (PocilloporaBase) for the cauliflower coral, *Pocillopora damicornis* BMC Genomics 2011 12 585 10.1186/1471-2164-12-585 22126435 PMC3339375

[B37] Sun J Chen Q Lun JC Xu J Qiu JW PcarnBase: Development of a transcriptomic database for the brain coral *Platygyra carnosus* Mar Biotechnol 2013 15 244 451 10.1007/s10126-012-9482-z 22875536

[B38] Cossins A Fraser J Hughes M Gracey A Post-genomic approaches to understanding the mechanisms of environmentally induced phenotypic plasticity J Exp Biol 2006 209 2328 2336 10.1242/jeb.02256 16731809

[B39] Weston AJ Dunlap WC Shick JM Klueter A Iglic K Vukelic A Starcevic A Ward M Wells ML Trick CG Long PF A profile of an endosymbiont-enriched fraction of the coral *Stylophora pistillata* reveals proteins relevant to microbial-host interactions Mol Cell Proteomics 2012 11 M111.015487 10.1074/mcp.M111.015487 22351649 PMC3433924

[B40] Starcevic A Dunlap WC Cullum J Shick JM Hranueli D Long PF Gene expression in the scleractinian *Acropora microphthalma* exposed to high solar irradiance reveals elements of photoprotection and coral bleaching PLoS One 2010 5 e13975 10.1371/journal.pone.0013975 21103042 PMC2980464

[B41] Miller DJ Ball EE Technau U Cnidarians and ancestral genetic complexity in the animal kingdom Trends Genet 2005 21 536 539 10.1016/j.tig.2005.08.002 16098631

[B42] Putnam NH Srivastava M Hellsten U Dirks B Chapman J Salamov A Terry A Shapiro H Lindquist E Kapitonov VV Jurka J Genikhovich G Grigoriev IV Lucas SM Steele RE Finnerty JR Technau U Martindale MQ Rokhsar DS Sea anemone genome reveals ancestral eumetazoan gene repertoire and genomic organization Science 2007 317 86 94 10.1126/science.1139158 17615350

[B43] Kortschak RD Samuel G Saint R Miller DJ EST analysis of the cnidarians *Acropora millepora* reveals extensive gene loss and rapid sequence divergence in the model invertebrates Curr Biol 2003 13 2190 2195 10.1016/j.cub.2003.11.030 14680636

[B44] Chapman JA Kirkness EF Simakov O Hampson SE Mitros T Weinmaier T Rattei T Balasubramanian PG Borman J Busam D Disbennett K Pfannkoch C Sumin N Sutton GG Viswanathan LD Walenz B Goodstein DM Hellsten U Kawashima T Prochnik SE Putnam NH Shu S Blumberg B Dana CE Gee L Kibler DF Law L Lindgens D Martinez DE Peng J Wigge PA **The dynamic genome of the *****Hydra*** Nature 2010 464 591 596 10.1038/nature08830 PMC4479502 20228792

[B45] Shinzato C Shoguchi E Kawashima T Hamada M Hisata K Tanaka M Fujie M Fujiwara M Koyanagi R Ikuta T Fujiyama A Miller DJ Satoh N Using the *Acropora digitifera* genome to understand coral responses to environmental change Nature 2011 476 320 323 10.1038/nature10249 21785439

[B46] Coral genome sequence data usage policy[http://coralbase.org/accounts/register/]

[B47] ZoophyteBase coral proteome database [http://bioserv7.bioinfo.pbf.hr/Zoophyte/index.jsp]

[B48] Genome Sequencing/Annotation Projectshttp://marinegenomics.oist.jp/genomes/download?%20project_id=3]

[B49] Finn RD Clements J Eddy SR HMMER web server: interactive sequence similarity searching Nucleic Acids Res 2011 39 Web Server Issue W29 W37 21593126 10.1093/nar/gkr367 PMC3125773

[B50] Camacho C Coulouris G Avagyan V Ma N Papadopoulos J Bealer K Madden TL BLAST+: architecture and applications BMC Bioinforma 2009 10 421 10.1186/1471-2105-10-421 PMC2803857 20003500

[B51] KEGG Kyoto Encyclopedia of Genes and Genomes[http://www.genome.jp/kegg/]

[B52] Apache Solr™ [http://lucene.apache.org/solr/]21097779

[B53] Conserved Domain Database(CDD) [http://www.ncbi.nlm.nih.gov/Structure/cdd/cdd.shtml]

[B54] Stanley GD Jr Photosymbiosis and the evolution of modern coral reefs Science 2006 312 857 858 10.1126/science.1123701 16690848

[B55] Dubinski Z Falkowski P Dubinski Z, Stambler N Light as a source of information and energy in zooxanthellate corals Coral Reefs: And Ecosystem in Transition 2011 Berlin: Springer-Verlag 107 118

[B56] Dunn SR Schnitzler CE Wies VM Apoptosis and autophagy as mechanisms of dinoflagellate symbiont release during cnidarian bleaching: every which way you lose Proc Biol Soc 2007 274 3079 3085 10.1098/rspb.2007.0711 PMC2293937 17925275

[B57] Downs CA Kramarsky-Winter E Martinez J Kushmaro A Woodley CM Loya Y Ostrander GK Symbiophagy as a cellular mechanism for coral bleaching Autophagy 2009 5 211 216 10.4161/auto.5.2.7405 19066451

[B58] Muscatine L Pool RR Regulation of numbers of intracellular alage Proc R Soc B 1979 204 131 139 10.1098/rspb.1979.0018 36618

[B59] Hutagalung AH Novick PJ Role of Rab GTPases in membrane traffic and cell physiology Physiol Rev 2011 91 119 149 10.1152/physrev.00059.2009 21248164 PMC3710122

[B60] Hong M-C Huang Y-S Lin W-W Fang L-S Chen M-C ApRab3, a biosynthetic Rab protein, accumulates on the maturing phagosomes and symbiosomes in the tropical sea anemone*, Aiptasia pulchella* Comp Biochem Physiol B Biochem Mol Biol 2009 152 249 259 10.1016/j.cbpb.2008.12.005 19110066

[B61] Hong M-C Huang J-S Song P-C Lin W-W Fang L-S Chen M-C Cloning and characterization of ApRab4, a recycling Rab protein of *Aiptasia pulchella*, and its implication in the symbiosome biogenesis Mar Biotechnol 2009 11 771 785 10.1007/s10126-009-9193-2 19459008

[B62] Chen MC Cheng YM Hong MC Fang LS Molecular cloning of Rab5 (ApRab5) in *Aiptasia pulchella* and its retention in phagosomes harbouring live zooxanthellae Biochem Biophys Res Commun 2004 324 1024 1033 10.1016/j.bbrc.2004.09.151 15485657

[B63] Chen MC Cheng YM Sung PJ Kuo CE Fang LS Molecular identification of Rab7 (ApRab7) in *Aiptasia pulchella* and its exclusion from phagosomes harboring zooxanthellae Biochem Biophys Res Commun 2003 308 586 595 10.1016/S0006-291X(03)01428-1 12914791

[B64] Chen MC Hong MC Huang YS Liu MC Cheng YM Fang LS ApRab11, a cnidarian homologue of the recycling regulatory protein Rab11, is involved in the establishment and maintenance of the *Aiptasia-Symbiodinium* endosymbiosis Biochem Biophys Res Commun 2005 338 1607 1616 10.1016/j.bbrc.2005.10.133 16288726

[B65] Collins RN Rab and ARF GTPase regulation of exocytosis Mol Membr Biol 2003 20 105 115 10.1080/0968768031000085892 12851068

[B66] Deneka M Neeft M van der Sluijs P Regulation of membrane transport by Rab GTPases Crit Rev Biochem Mol Biol 2003 38 121 142 10.1080/713609214 12749696

[B67] Vassilieva EV Nusrat A Vesicular trafficking: molecular tools and targets Methods Mol Biol 2008 440 3 14 10.1007/978-1-59745-178-9_1 18369933

[B68] Ohya T Miaczynska M Coskun Ü Lommer B Runge A Drechsel D Kalaidzidis Y Zerial M Reconstruction of Rab- and SNARE-dependent membrane fusion by synthetic endosomes Nature 2009 459 1091 1097 10.1038/nature08107 19458617

[B69] SNARE Database [http://bioinformatics.mpibpc.mpg.de/snare/snareQueryPage.jsp]

[B70] Kloepper TH Kienle CN Fasshauer D An elaborate classification of SNARE proteins sheds light on the conservation of the eukaryotic endomembrane system Mol Biol Cell 2007 18 3463 3471 10.1091/mbc.E07-03-0193 17596510 PMC1951749

[B71] Hay JC Chao DS Kuo CS Scheller RH Protein interactions regulating vesicle transport between the endoplasmic reticulum and Golgi apparatus in mammalian cells Cell 1997 89 149 158 10.1016/S0092-8674(00)80191-9 9094723

[B72] Masuda ES Huang BC Fisher JM Luo Y Scheller RH Tomosyn binds t-SNARE proteins via a VAMP-like coiled coli Neuron 1998 21 479 480 10.1016/S0896-6273(00)80559-0 9768835

[B73] Scales SJ Hesser BA Masuda ES Scheller RH Amisyn, a novel syntaxin-binding protein that may regulate SNARE complex assembly J Biol Chem 2002 227 28271 28279 12145319 10.1074/jbc.M204929200

[B74] Jahn R Scheller RH SNAREs – Engines for membrane fusion Nat Rev Mol Cell Biol 2006 7 631 643 10.1038/nrm2002 16912714

[B75] Muscatine L Pool RR Cernichiari E Some factors influencing selective release of soluble organic material by zooxanthellae from reef corals Mar Biol 1972 13 298 308 10.1007/BF00348077

[B76] Sutton DC Hoegh-Guldberg O Host-zooxanthellae interactions in four temperate marine invertebrate symbioses: assessment of the effect of host extracts on symbionts Biol Bull 1990 178 175 186 10.2307/1541975 29314935

[B77] Masuda K Miyachi S Maruyama T Sensitivity of zooxanthellae and non-symbiotic microalgae to stimulation of photosynthate excretion by giant clam tissue homogenate Mar Biol 1994 118 687 693 10.1007/BF00347517

[B78] Narayanan A Nogueira ML Ruyechan WT Kristie TM Combinatorial transcription of *Herpes simplex* virus and *Varicella zoster* virus intermediate early genes is strictly determined by cellular coactivator HCF-1 J Biol Chem 2005 280 1369 1375 15522876 10.1074/jbc.M410178200

[B79] Lee S Horn V Julien E Liu Y Wysocka J Bowerman B Hengartner MO Herr W **Epigenetic regulation of histone H3 serine 10 phosphorylation status by HCF-1 proteins in *****C. elegans *****and mammalian cells** PLoS One 2007 2 e1213 10.1371/journal.pone.0001213 18043729 PMC2082077

[B80] Kristie TM Liang Y Vogel JL Control of α-herpesvirus IE gene expression by HCF-1 coupled chromatin modification activities Biochem Biophys Acta 2010 1799 257 265 10.1016/j.bbagrm.2009.08.003 19682612 PMC2838944

[B81] Christensen RG Enuameh MS Noyes MB Brodsky MH Wolfe SA Stormo GD Recognition models to predict DNA-binding specificities of homeodomain proteins Bioinformatics 2012 28 i84 i89 10.1093/bioinformatics/bts202 22689783 PMC3371834

[B82] Mann RS Lelli KM Joshi R Hox specificity: unique roles for cofactors and collaborators Curr Top Dev Biol 2009 88 66 101 10.1016/S0070-2153(09)88003-4 PMC2810641 19651302

[B83] Finnerty JR Martindale MQ Ancient origins of axial patterning genes: Hox genes and ParaHox genes in the Cnidaria Evol Dev 1999 1 16 23 10.1046/j.1525-142x.1999.99010.x 11324016

[B84] Hislop NR de Jong D Hayward DC Ball EE Miller DJ Tandem organisation of independently duplicated homeobox genes in the basal cnidarian *Acropora millepora* Dev Genes Evol 2005 215 268 273 10.1007/s00427-005-0468-y 15702325

[B85] Ryan JF Mazza ME Pang K Matus DQ Baxevanis AD Martindale MQ Finnerty JR **Pre-bilaterian origins of the Hox cluster and the Hox code: evidence from the sea anemone, *****Nematostella vectensis*** PLoS One 2007 2 e153 10.1371/journal.pone.0000153 17252055 PMC1779807

[B86] Larroux C Fahey B Degnan SM Adamski M Rokhsar DS Degnan BM The NK homeobox gene cluster predates the origin of Hox genes Curr Biol 2007 17 706 710 17379523 10.1016/j.cub.2007.03.008

[B87] Dowid IB Chitnis AB LIM homeobox genes and the CNS: a close relationship Neuron 2001 30 301 303 10.1016/S0896-6273(01)00307-5 11394990

[B88] Srivastava M Larroux C Lu CD Mohanty K Chapman J Degnan BM Rokhsar DS Early evolution of the LIM homeobox gene family BMC Biol 2010 8 4 10.1186/1741-7007-8-4 20082688 PMC2828406

[B89] Ryan JF Burton PM Mazza ME Kwong GK Mullikin JC Finnerty JR **The cnidarian-bilaterian ancestor possessed at least 56 homeoboxes: evidence from the starlet sea anemone, *****Nematostella vectensis*** Genome Biol 2006 7 R64 10.1186/gb-2006-7-7-r64 16867185 PMC1779571

[B90] Saito T Sawamoto K Okano H Anderson DJ Mikoshiba K Mammalian BarH homologue is a potential regulator of neural bHLH genes Dev Biol 1998 199 216 225 10.1006/dbio.1998.8889 9698441

[B91] Habas S Kato Y He X Wnt/Frazzled activation of Rho regulates vertebrate gastrulation and requires a novel Formin homology protein Daam1 Cell 2001 107 843 854 10.1016/S0092-8674(01)00614-6 11779461

[B92] Matusek T Djiane A Jankovics F Brunner D Mlodzik M Mihály J The *Drosophila* formin DAAM regulates the tracheal cuticle pattern through organizing the actin cytoskeleton Development 2006 133 957 966 10.1242/dev.02266 16469972

[B93] Li D Hallett MA Zhu W Rubart M Liu Y Yang Z Chen H Haneline LS Chan RJ Schwartz RJ Field LJ Atkinson SJ Shou W Dishevelled-associated activator of morphogenesis 1 (Daam1) is required for heart morphogenesis Development 2011 138 303 315 10.1242/dev.055566 21177343 PMC3005605

[B94] Wada Y Kitamoto K Kanbe T Tanaka K Anraku Y The *SLP1* gene of *Saccharomyces cerevisiae* is essential for vacuolar morphogenesis and function Mol Cell Biol 1990 10 2214 2223 2183024 10.1128/mcb.10.5.2214-2223.1990 PMC360569

[B95] Bragdon B Moseychuk O Saldanha S King D Julian J Nohe A Bone morphogenetic proteins: a critical review Cell Signal 2011 23 609 620 10.1016/j.cellsig.2010.10.003 20959140

[B96] Martin VJ Photoreceptors of cnidarians Can J Zool 2002 80 1703 1722 10.1139/z02-136

[B97] Mason BM Cohen JH Long-wavelength photosensitivity in coral planula larvae Biol Bull 2012 222 88 92 22589399 10.1086/BBLv222n2p88

[B98] Gorbunov MY Falkowski PG Photoreceptors in the cnidarian host allow symbiotic corals to sense blue moonlight Limnol Oceanogr 2002 47 309 315 10.4319/lo.2002.47.1.0309

[B99] Levy O Appelbaum L Leggat W Gothlif Y Hayward DC Miller DJ Hoegh-Guldberg O Light-responsive cryptochromes from a simple multicellular animal, the coral *Acropora millepora* Science 2007 318 467 470 10.1126/science.1145432 17947585

[B100] Vize PD Transcriptome analysis of the circadian regulatory network in the coral *Acropora millepora* Biol Bull 2009 216 131 137 19366924 10.1086/BBLv216n2p131

[B101] Brady AK Snyder KA Vize PD **Circadian cycles of gene expression in the coral, *****Acropora millepora*** PLoS One 2011 6 e25072 10.1371/journal.pone.0025072 21949855 PMC3176305

[B102] Ishiura M Kutsuna S Aoki S Iwasaki H Andersson CR Tanabe A Golden SS Johnson CH Kondo T Expression of a gene cluster *kaiABC* as a circadian feedback process in cyanobacteria Science 1998 281 1519 1523 9727980 10.1126/science.281.5382.1519

[B103] Nishiwaki T Satomi Y Nakajima M Lee C Kiyohara R Kageyama H Kitayama Y Temamoto M Yamaguchi A Hijikata A Go M Iwasaki H Takao T Kondo T Role of KaiC phosphorylation in the circadian clock system of *Synechococcus elongatus* PCC 7942 Proc Natl Acad Sci USA 2004 101 13927 13932 10.1073/pnas.0403906101 15347812 PMC518855

[B104] Xu Y Mori T Qin X Yan H Egli M Johnson CH Intramolecular regulation of phosphorylation status of the circadian clock protein KaiC PLoS One 2009 4 e7509 10.1371/journal.pone.0007509 19946629 PMC2778140

[B105] Nakahira Y Katayama M Miyashita H Katsuna S Iwasaki H Oyama T Kondo T Global gene repression by KaiC as a master process of prokaryotic circadian system Proc Natl Acad Sci USA 2004 101 881 885 10.1073/pnas.0307411100 14709675 PMC321775

[B106] Kondo T Ishiura M The circadian clock of cyanobacteria Bioessays 2000 22 10 15 10.1002/(SICI)1521-1878(200001)22:1<10::AID-BIES4>3.0.CO;2-A 10649285

[B107] Hardin PE Hall JC Rosbash M Feedback of the *Drosophila* period gene product on circadian cycling of its messenger RNA levels Nature 1990 343 536 540 10.1038/343536a0 2105471

[B108] Dunlap JC Molecular bases for circadian clocks Cell 1999 96 271 290 10.1016/S0092-8674(00)80566-8 9988221

[B109] Reitzel AM Behrendt L Tarrant AM Light entrained rhythmic gene expression in the sea anemone *Nematostella vectensis*: the evolution of the animal circadian clock PLoS One 2010 5 e12805 10.1371/journal.pone.0012805 20877728 PMC2943474

[B110] Gibbons IR Cilia and flagella of eukaryotes J Cell Biol 1981 91 107s 124s 10.1083/jcb.91.3.107s 6459326 PMC2112791

[B111] Linck RW Tektins and microtubules Adv Mol Cell Biol 1990 3 35 63

[B112] Fox LA Sawin KE Sale WS Kinesin-related proteins in eukaryotic flagella J Cell Sci 1994 107 1545 1550 7962196 10.1242/jcs.107.6.1545

[B113] Amos LA The tektin family of microtubule-stabilizing proteins Genome Biol 2008 9 229 18671835 10.1186/gb-2008-9-7-229 PMC2530864

[B114] Kozminski KG Beech PL Rosenbaum JL The *Chlamydomonas* kinesin-like protein FLA10 is involved in motility associated with the flagellar membrane J Cell Biol 1995 131 1517 1527 10.1083/jcb.131.6.1517 8522608 PMC2120669

[B115] Bartlett DH Frantz BB Matsumura P Flagellar activators FlbB and FlaI: gene sequences and 5′ consensus sequences of operons under FlbB and FlaI control J Bacteriol 1988 170 1575 1581 2832369 10.1128/jb.170.4.1575-1581.1988 PMC211004

[B116] Aldridge P Karlinsey J Hughes KT The type III secretion chaperone FlgN regulates flagellar assembly via a negative feedback loop containing its chaperone substrates FlgK and FlgL Mol Microbiol 2003 49 1333 1345 10.1046/j.1365-2958.2003.03637.x 12940991

[B117] Ferris HU Furukawa Y Minamino T Kroetz MB Kihara M Namba K Macnab RM FlhB regulates ordered export of flagellar components via autocleavage mechanisms J Biol Chem 2005 280 41236 41242 10.1074/jbc.M509438200 16246842

[B118] Mattick JS Type IV pili and twitching motility Annu Rev Microbiol 2002 56 289 314 10.1146/annurev.micro.56.012302.160938 12142488

[B119] Starcevic A Akthar S Dunlap WC Shick JM Hranueli D Cullum J Long PF Enzymes of the shikimate acid pathway encoded in the genome of a basal metazoan, *Nematostella vectensis*, have microbial origins Proc Natl Acad Sci U S A 2008 105 2533 2537 10.1073/pnas.0707388105 18268342 PMC2268171

[B120] Rebbapragada A Johnson MS Harding GP Zuccarelli AJ Fletcher HM Zhulin IB Taylor BL The Aer protein and the serine chemoreceptor Tsr independently sense intracellular energy levels and transduce oxygen, redox, and energy signals for *Escherichia coli* behavior Proc Natl Acad Sci USA 1997 94 10541 10546 10.1073/pnas.94.20.10541 9380671 PMC23396

[B121] Troemei ER Chou JH Dwyer ND Colbert HA Bargmann CI Divergent seven transmembrane receptors are candidate chemosensory receptors in *C. elegans* Cell 1995 83 207 218 10.1016/0092-8674(95)90162-0 7585938

[B122] Sen M Shah A Marsh L Two types of alpha-factor receptor determinants for pheromone specificity in the mating-incompatible yeasts *S. cerevisiae* and *S. kluyveri* Curr Genet 1997 31 235 240 10.1007/s002940050200 9065386

[B123] Gestwicki JE Lamanna AC Harshey RM McCarter LL Kiessling LL Adler J Evolutionary conservation of methyl accepting chemotaxis protein location in Bacteria and Archaea J Bacteriol 2000 182 6499 6502 10.1128/JB.182.22.6499-6502.2000 11053396 PMC94798

[B124] Capra EJ Laub MT Evolution of two-component signal transduction systems Annu Rev Microbiol 2012 66 325 347 10.1146/annurev-micro-092611-150039 22746333 PMC4097194

[B125] Koretke KK Lupas AN Warren PV Rosenberg M Brown JR Evolution of two-component signal transduction Mol Biol Evol 2000 17 1956 1970 10.1093/oxfordjournals.molbev.a026297 11110912

[B126] DeVries ME Kelvin AA Xu L Ran L Robertson J Kelvin DJ Defining the origins and evolution of the chemokine/chemokine receptor system J Immunol 2006 176 401 415 16365434 10.4049/jimmunol.176.1.401

[B127] Holland ND Early central nervous system evolution: an era of skin brains? Nat Rev Neurosci 2003 4 617 627 10.1038/nrn1175 12894237

[B128] Galliot B Quiquand M Ghila L de Rosa R Miljkovic-Licina M Chera S Origins of neurogenesis, a cnidarian view Dev Biol 2009 332 2 24 10.1016/j.ydbio.2009.05.563 19465018

[B129] Nakanishi N Renfer E Technau U Rentzsch F Nervous systems of the sea anemone *Nematostella vectensis* are generated by ectoderm and endoderm and shaped by distinct mechanisms Development 2012 139 347 357 10.1242/dev.071902 22159579

[B130] Sakarya O Armstrong KA Adamska M Adamski M Wang IF Tidor B Degnan BM Oakley TH Kosik KS A post-synaptic scaffold at the origin of the animal kingdom PLoS One 2007 2 e506 10.1371/journal.pone.0000506 17551586 PMC1876816

[B131] Miljkovik-Licina M Gauchat D Galliot B Neuronal evolution: analysis of regulatory genes in a first-evolved nervous system, the hydra nervous system Biosystems 2004 76 75 87 10.1016/j.biosystems.2004.05.030 15351132

[B132] Marlow HQ Srivastava M Matus DQ Rokhsar D Martindale MQ Anatomy and development of the nervous system of *Nematostella vectensis*, an anthozoan cnidarian Dev Neurobiol 2009 69 235 254 10.1002/dneu.20698 19170043

[B133] Kass-Simon G Pierobon P Cnidarian chemical neurotransmission, an updated overview Comp Biochem Physiol A Mol Intergr Physiol 2007 146 9 25 10.1016/j.cbpa.2006.09.008 17101286

[B134] Grimmelikhuijzen CJP Westfall JA Breidbach O, Kutch W The nervous systems of cnidarians The Nervous Systems of Invertebrates. An Evolutionary and Comparative Approach 1995 Basel: Brikhäuser Verlag 7 24

[B135] Sleeman MW Anderson KD Lambert PD Yancopoulos GD Wiegand SJ The ciliary neurotrophic factor and its receptor, CNFRTA alpha Pharm Acta Helv 2000 74 265 272 10.1016/S0031-6865(99)00050-3 10812968

[B136] Kass-Simon G Scappaticci AA Jr The behavioural and developmental physiology of nematocysts Can J Zool 2002 80 1772 1794 10.1139/z02-135

[B137] Plachetzki DC Fong CR Oakley TH Cnidocyte discharge is regulated by light and opsin-mediated phototransduction BMC Biol 2012 10 17 10.1186/1741-7007-10-17 22390726 PMC3329406

[B138] Coates MM Visual ecology and functional morphology of Cubozoa (Cnidaria) Integr Comp Biol 2003 43 542 548 10.1093/icb/43.4.542 21680462

[B139] Koyanagi M Takano K Tsukamoto H Ohtsu K Tokunaga F Terakita A Jellyfish vision starts with cAMP signalling mediated by opsin-G_S_ cascade Proc Natl Acad Sci U S A 2008 105 15576 15580 10.1073/pnas.0806215105 18832159 PMC2563118

[B140] Shichida Y Matsuyama T Evolution of opsins and phototransduction Philos Trans R Soc Lond B Biol Sci 2009 364 2881 2895 10.1098/rstb.2009.0051 19720651 PMC2781858

[B141] Beliaev A Learmonth DA Soares-da-Silva P Synthesis and biological evaluation of novel, peripherally selective chromanyl imidazolethione-based inhibitors of dopamine beta-hydroxylase J Med Chem 2006 49 1191 1197 10.1021/jm051051f 16451083

[B142] Grimmelikhuijzen CJP Williamson M Hansen GN Neuropeptides in cnidarians Can J Zool 2002 80 1690 1702 10.1139/z02-137

[B143] Attenborough RM Hayward DC Kitahara MV Miller DJ Ball EE A “neural” enzyme in nonbilaterian animals and algae: preneural origins for peptidylglycine α-amindating monooxygenase Mol Biol Evol 2012 29 3095 3109 10.1093/molbev/mss114 22496439

[B144] Allemand D Tambutté E Zoccola D Tambutté S Dubinsky Z, Stambler N Coral calcification, cells to reefs Coral Reefs: An Ecosystem in Transition 2011 Dordrecht: Springer 119 150

[B145] Bénazet-Tambutté S Allemand D Joubert J Permeability of the oral epithelial layers in cnidarians Mar Biol 1996 126 43 53 10.1007/BF00571376

[B146] Al-Horani FA Al-Moghrabi SM de Beer D The mechanism of calcification and its relation to photosynthesis and respiration in the scleractinian coral *Galaxea fascicularis* Mar Biol 2003 142 419 429

[B147] Zoccola D Tambutté E Sénégas-Balas F Michiels JF Failla JP Jaubert J Allemand D Cloning of a calcium channel α1 subunit from the reef-building coral, *Stylophora pistillata* Gene 1999 227 157 167 10.1016/S0378-1119(98)00602-7 10023047

[B148] Zoccola D Tambutté E Kulhanek E Puverel S Scimeca JC Allemand D Tambutté S Molecular cloning and localisation of a PMCA P-type calcium ATPase from the coral *Stylophora pistillata* Biochim Biophys Acta 2004 1663 117 126 10.1016/j.bbamem.2004.02.010 15157614

[B149] Moya A Tambutté S Bertucci A Tambutté E Lotto S Vullo D Supuran CT Allemand D Zoccola D Carbonic anydrase in the scleractinian coral *Stylophora pistillata*: characterisation, localisation, and role in biomineralisation J Biol Chem 2008 283 25475 25484 10.1074/jbc.M804726200 18617510

[B150] Gattuso J-P Allemand D Frankignoulle M Photosynthesis and calcification at cellular, organismal and community levels in coral reefs: A review on interactions and control by carbonate chemistry Amer Zool 1999 39 160 183

[B151] Allemand D Ferrier-Pagè C Furla P Houlbrèque F Puverel S Reynaud S Tambutté É Tambutté S Zoccola D Biomineralisation in reef-building corals: from molecular mechanisms to environmental control C R Palevol 2004 3 453 467 10.1016/j.crpv.2004.07.011

[B152] Venn A Tambutté E Holcomb M Allemand D Tambutté S Live tissue imaging shows corals elevate pH under their calcifying tissues relative to seawater PLoS One 2011 6 e20013 10.1371/journal.pone.0020013 21637757 PMC3103511

[B153] Kaniewska P Campbell PR Kline DI Rodriguez-Lanetty M Miller DJ Dove S Hoegh-Guldberg O Major cellular and physiological impacts of ocean acidification on a reef building coral PLoS One 2012 7 e34659 10.1371/journal.pone.0034659 22509341 PMC3324498

[B154] Catterall WA Voltage-gated calcium channels Cold Spring Harb Perspect Biol 2011 3 003947 10.1101/cshperspect.a003947 PMC3140680 21746798

[B155] Bertucci A Tembutté S Supuran CT Allemand D Zoccola D A new coral carbonic anhydrase in *Stylophora pistillata* Mar Biotechnol 2011 13 992 1002 10.1007/s10126-011-9363-x 21318259

[B156] Morita M Iguchi A Takamura A Roles of calmodulin and calcium/calmodulin-dependent protein in flagellate motility regulation in the coral *Acropora digitifera* Mar Biotechnol 2009 11 118 123 10.1007/s10126-008-9127-4 18661183

[B157] Lane CE Archibald JM The eukaryotic tree of life: endosymbionts takes its TOL Trends Ecol Evol 2008 32 268 275 18378040 10.1016/j.tree.2008.02.004

[B158] Keeling PJ Functional and ecological impacts of horizontal gene transfer in eukaryotes Curr Opin Genet Dev 2009 19 613 619 10.1016/j.gde.2009.10.001 19897356

[B159] Bock R The give-and-take of DNA: horizontal gene transfer in plants Trends Plant Sci 2010 15 11 22 19910236 10.1016/j.tplants.2009.10.001

[B160] Balskus EP Walsh CT The genetic and molecular basis for sunscreen biosynthesis in cyanobacteria Science 2010 329 1653 1656 10.1126/science.1193637 20813918 PMC3116657

[B161] Waller RF Stamovits CH Keeling PJ Lateral gene transfer of a multigene region from cyanobacteria to dinoflagellates resulting in a novel plastid-targeted fusion protein Mol Biol Evol 2006 23 1437 1443 10.1093/molbev/msl008 16675503

[B162] Richards TA Dacks JB Campbell SA Blanchard JL Foster PG McLeod R Roberts CW Evolutionary origins of the eukaryotic shikimate pathway: gene fusions, horizontal transfer, and endosymbiotic replacement Eukaryot Cell 2006 5 1517 1531 10.1128/EC.00106-06 16963634 PMC1563581

[B163] Habetha M Bosch TC Symbiotic *Hydra* express a plant-like peroxidase gene during oogenesis J Exp Biol 2005 208 2157 2165 10.1242/jeb.01571 15914659

[B164] Technau U Miller MA Bridge D Steele RE Arrested apoptosis of nurse cells during *Hydra* oogenesis and embryogenesis Dev Biol 2003 260 191 206 10.1016/S0012-1606(03)00241-0 12885564

[B165] Rumpho ME Worful JM Lee J Kannan K Tyler MS Bhattacharya D Moustafa A Manhart JR Horizontal gene transfer of the algal nuclear gene *psbO* to the photosynthetic sea slug *Elysia chlorotica* Proc Natl Acad Sci U S A 2008 105 17867 17871 10.1073/pnas.0804968105 19004808 PMC2584685

[B166] Rumpho ME Pelletreau KN Moustafa A Bhattacharya D The making of a photosynthetic animal J Exp Biol 2011 214 303 311 10.1242/jeb.046540 21177950 PMC3008634

[B167] Pierce SK Curtis NE Cell biology of the chloroplast symbiosis in sacaglossan sea slugs Int Rev Cell Mol Biol 2012 293 123 148 22251560 10.1016/B978-0-12-394304-0.00009-9

[B168] Wägele H Deusch O Händeler K Martin R Schmitt V Christa G Pinzger B Gould SB Dagan T Klussmann-Kolb A Martin W Transcriptomic evidence that longevity of acquired plastids in the photosynthetic slugs *Elysia timida* and *Plakobranchus ocellatus* does not entail lateral transfer of algal nuclear genes Mol Biol Evol 2011 28 699 706 10.1093/molbev/msq239 20829345 PMC3002249

[B169] Pierce SK Fang X Schwartz JA Jiang X Zhao W Curtis NE Kocot KM Yang B Wang J Transcriptomic evidence for the expression of horizontally transferred algal nuclear genes in the photosynthetic sea slug, *Elysia chlorotica* Mol Biol Evol 2012 29 1545 1556 10.1093/molbev/msr316 22319135

[B170] Lang BF O’Kelly C Nerad T Gray MW Burger G The closest unicellular relatives of animals Curr Biol 2002 12 1773 1778 10.1016/S0960-9822(02)01187-9 12401173

[B171] Ruiz-Trillo I Roger AJ Burger G Gray MW Lang BF A phylogenomic investigation into the origins of metazoa Mol Biol Evol 2008 25 664 672 10.1093/molbev/msn006 18184723

[B172] Sun G Yang Z Ishwar A Huang J Algal genes in the closest relatives of animals Mol Biol Evol 2010 27 2879 2889 10.1093/molbev/msq175 20627874

[B173] Ohad I Dal Bosco C Herrmann RG Meurer J Photosysytem II proteins PsbL and PsbJ regulate electron flow to the plastoquinone pool Biochemistry 2004 43 2297 2308 10.1021/bi0348260 14979726

[B174] Yamazaki S Nomata J Fujita Y Differential operation of dual protochlorophyllide reductases for chlorophyll biosynthesis in response to environmental oxygen levels in the cyanobacterium *Leptolyngbya boryana* Plant Physiol 2006 142 911 922 10.1104/pp.106.086090 17028153 PMC1630749

[B175] Pruzinská A Anders I Aubry S Schenk N Tapernoux-Lüthi E Müller T Kräutler B Hörtensteiner S In vivo participation of red chlorophyll catabolite reductase in chlorophyll breakdown Plant Cell 2007 19 369 387 10.1105/tpc.106.044404 17237353 PMC1820978

[B176] Harada J Saga Y Yaeda Y Oh-Oka H Tamiaki H In vitro activity of C-20 methyltransferase, BchU, involved in bacteriochlorophyll *c* biosynthetic pathway in green sulfur bacteria FEBS Lett 2005 579 1983 1987 10.1016/j.febslet.2005.01.087 15792807

[B177] Long H King PW Ghirardi ML Kim K Hydrogenase/ferredoxin charge-transfer complexes: effect of hydrogenase mutations on the complex association J Phys Chem A 2009 113 4060 4067 10.1021/jp810409z 19317477

[B178] Spence E Dunlap WC Shick JM Long PF Redundant pathways of sunscreen biosynthesis in a cyanobacterium ChemBioChem 2012 13 531 533 10.1002/cbic.201100737 22278966

[B179] Wegkamp A van Oorschot W de Vos WM Smid EJ Characterization of the role of *para*-aminobenzoic acid biosynthesis in folate production by *Lactococcus lactis* Appl Environ Microbiol 2007 73 2673 2681 10.1128/AEM.02174-06 17308179 PMC1855612

[B180] Sharon I Tzahor S Williamson S Shmoish M Man-Aharonovich D Rusch DB Yooseph S Zeidner G Golden SS Mackey SR Adir N Weingart U Horn D Venter JC Mandel-Gutfreund YM Béjà O Viral photosynthetic reaction center genes and transcripts in the marine environment ISME J 2007 1 492 501 10.1038/ismej.2007.67 18043651

[B181] Wang Q Jantaro S Lu B Majeed W Bailey M He Q The high light-inducible polypeptides stabilize trimeric photosystem I complex under high light conditions in *Synechocystis* PCC 6803 Plant Physiol 2008 147 1239 1250 10.1104/pp.108.121087 18502976 PMC2442545

[B182] Mann NH Cook A Millard A Bailey S Clokie M Marine ecosystems: bacterial photosynthesis genes in a virus Nature 2003 424 741 12917674 10.1038/424741a

[B183] Lindell D Sullivan MB Johnson ZI Tolonen AC Rohwer F Chisholm SW Transfer of photosynthesis genes to and from *Prochlorococcus* viruses Proc Natl Acad Sci U S A 2004 101 11013 11018 10.1073/pnas.0401526101 15256601 PMC503735

[B184] Mann NH Clokie MRJ Millard A Cook A Wilson WH Wheatley PJ Letarov A Krisch HM The genome of S-PM2, a “photosynthetic” T4-type bacteriophage that infects marine *Synechococcus* strains J Bacteriol 2005 187 3188 3200 10.1128/JB.187.9.3188-3200.2005 15838046 PMC1082820

[B185] van Oppen MJH Leong J-A Gates RD Coral-virus interactions: a double-edged sword? Symbiosis 2009 47 1 8 10.1007/BF03179964

[B186] Nagasaki K Tomaru Y Shirai Y Takano Y Mizumoto H Dinoflagellate-infecting viruses J Mar Biol Assoc UK 2006 86 469 474 10.1017/S0025315406013361

[B187] Lohr J Munn CB Wilson WH Characterization of a latent virus-like infection of symbiotic zooxanthellae Appl Environ Microbiol 2007 73 2976 2981 10.1128/AEM.02449-06 17351090 PMC1892877

[B188] Moran NA Symbiosis as an adaptive process and source of phenotypic complexity Proc Natl Acad Sci U S A 2007 104 Suppl 1 8627 8633 17494762 10.1073/pnas.0611659104 PMC1876439

[B189] Falkowski PG Dubinsky Z Muscatine L Porter JW Light and the bioenergentics of a symbiotic coral Bioscience 1984 34 705 709 10.2307/1309663

[B190] Muscatine L D’Elia CF The uptake, retention and release of ammonium by reef corals Limnol Oceanogr 1978 23 725 734 10.4319/lo.1978.23.4.0725

[B191] Rahav O Dubinsky Z Achituv Y Falkowski PG Ammonium metabolism in the zooxanthellate coral, *Stylophora pistillata* Proc R Soc Lond B 1989 236 325 337 10.1098/rspb.1989.0026

[B192] Kawaguti S Ammonium metabolism of the reef corals Biol J Okayama Univ 1953 1 171 176

[B193] Kühl M Cohen Y Dalsgaard T Jørgensen BB Revsbech NP Microenvironment and photosynthesis of zooxanthellae in scleractinian corals studied with microsensors for O2, pH and light Mar Ecol Prog Ser 1995 117 159 172

[B194] Gallon JR The oxygen sensitivity of nitrogenase: a problem for biochemists and micro-organisms Trends Biochem Sci 1981 6 19 23

[B195] Webb KL DuPaul WD Wiebe W Sottile W Johannes RE Enewetak (Eniwetok) atoll: aspects of the nitrogen cycle on a coral reef Limnol Oceanogr 1975 20 198 210 10.4319/lo.1975.20.2.0198

[B196] Crossland CJ Barnes DJ Dissolved nutrients and organic particulates in water flowing over coral reefs at Lizard Island Aust J Mar Freshwat Res 1983 34 835 844 10.1071/MF9830835

[B197] Wilkinson CR Williams DMB Sommarco PW Hogg RW Trott LA Rates of nitrogen fixation on coral reefs across the continental shelf of the central Great Barrier Reef Reef Mar Biol 1984 80 255 262 10.1007/BF00392820

[B198] Capone DG Dunham SE Horrigan SG Duguay LE Microbial nitrogen transformations in unconsolidated coral reef sediments Mar Ecol Prog Ser 1992 80 75 88

[B199] Hewson I Moisander PH Morrison AE Zehr JP Diazotrophic bacterioplankton in a coral reef lagoon: phylogeny, diel nitrogenase expression and response to phosphate enrichment ISME J 2007 1 78 91 10.1038/ismej.2007.5 18043616

[B200] Larkum AWD Kennedy IR Muller WJ Nitrogen fixation on a coral reef Mar Biol 1988 98 143 155 10.1007/BF00392669

[B201] Shashar N Cohen Y Loya Y Sar N Nitrogen fixation (acetylene reduction) in stony corals: evidence for coral-bacteria interactions Mar Ecol Prog Ser 1994 111 259 264

[B202] Lesser MP Mazel CH Gorbunov MY Falkowski PG Discovery of symbiotic nitrogen-fixing cyanobacteria in corals Science 2004 305 997 1000 10.1126/science.1099128 15310901

[B203] Mouchka ME Hewson I Harvell CD Coral-associated bacterial assemblages: current knowledge and the potential for climate-driven impacts Integr Comp Biol 2010 50 662 674 10.1093/icb/icq061 21558231

[B204] Olson ND Ainsworth TD Gates RD Takabayashi M Diazotrophic bacteria associated with Hawaiian *Montipora* coral: diversity and abundance in correlation with symbiotic dinoflagellates J Exp Mar Biol Ecol 2009 371 140 146 10.1016/j.jembe.2009.01.012

[B205] Lema KA Willis BL Bourne DG Corals form characteristic associations with symbiotic nitrogen-fixing bacteria Appl Environ Microbiol 2012 78 3136 3144 10.1128/AEM.07800-11 22344646 PMC3346485

[B206] Kellogg CA Tropical Archaea: diversity associated with the surface microlayer of corals Mar Ecol Prog Ser 2004 273 81 88

[B207] Wegley L Edwards R Rodriguez-Brito B Liu H Rohwer F Metagenomic analysis of the microbial community associated with the coral *Porites astreoides* Environ Microbiol 2007 9 2707 2719 10.1111/j.1462-2920.2007.01383.x 17922755

[B208] Siboni N Ben-Dov E Silvan A Kushmaro A Global distribution and diversity of coral-associated Archaea and their possible role in the coral holobiont nitrogen cycle Environ Microbiol 2008 10 2979 2990 10.1111/j.1462-2920.2008.01718.x 18707612

[B209] Rubio LM Ludden PW Biosynthesis of the iron-molybdenum cofactor of nitrogenase Annu Rev Microbiol 2008 62 93 111 10.1146/annurev.micro.62.081307.162737 18429691

[B210] Fujita Y Bauer CE Reconstitutuion of the light-independent protochlorophyllide reductase from purified Bchl and BchN-BchB subunits. *In vitro* confirmation of nitrogenase features of a bacteriochlorophyll biosynthesis enzyme J Biol Chem 2000 275 23583 23588 10.1074/jbc.M002904200 10811655

[B211] Sarma R Barney BM Hamilton TL Jones A Seefeld LC Peters JW Crystal structure of the L protein of *Rhodobacter sphaeroides* light-independent protochlorophyllide reductase with MgADP bound: a homologue of the nitrogenase Fe protein Biochemistry 2008 47 13004 13015 10.1021/bi801058r 19006326

[B212] Muraki N Nomata J Ebata K Mizoguchi T Shiba T Tamiaki H Kurisu G Fujita Y X-ray crystal structure of the light-independent protochlorophyllide reductase Nature 2010 465 110 114 10.1038/nature08950 20400946

[B213] Heyes DJ Hunter CN Making light work of enzyme catalysis: protochlorophyllide oxidoreductase Trends Biochem Sci 2005 30 642 649 10.1016/j.tibs.2005.09.001 16182531

[B214] Dean DR Bolin JT Zheng L Nitrogenase metalloclusters: structure, organisation, and synthesis J Bacteriol 1993 175 6737 6744 8226614 10.1128/jb.175.21.6737-6744.1993 PMC206795

[B215] Zheng L Dean DR Catalytic formation of a nitrogenase iron-sulfur cluster J Biol Chem 1994 269 18723 18726 8034623

[B216] Shah VK Stacey G Brill WJ Electron transport to nitrogenase J Biol Chem 1983 258 12064 12068 6352705

[B217] Hoover TR Santero E Porter S Kustu S The integration host factor stimulates interaction of RNA polymerase with FIFA, the transcriptional activator for nitrogen fixation operons Cell 1990 63 11 22 10.1016/0092-8674(90)90284-L 2208275

[B218] Merrick MJ Edwards RA Nitrogen control in bacteria Microbiol Mol Biol Rev 1995 59 604 622 10.1128/mr.59.4.604-622.1995 PMC239390 8531888

[B219] Kneip C Lockhart P Voss C Maier UG Nitrogen fixation in eukaryotes – new models for symbiosis BMC Evol Biol 2007 7 55 10.1186/1471-2148-7-55 17408485 PMC1853082

[B220] Beman JM Roberts KJ Wegley L Rohwer F Francis CA Distribution and diversity of archaeal ammonia momooxygenase genes associated with corals Appl Environ Microbiol 2007 73 5642 5647 10.1128/AEM.00461-07 17586663 PMC2042080

[B221] Campbell WH Nitrate reductase structure, function and regulation: Bridging the gap between biochemistry and physiology Annu Rev Plant Physiol Mol Biol 1999 50 277 303 10.1146/annurev.arplant.50.1.277 15012211

[B222] Einsle O Structure and function of formate-dependent cytochrome c nitrite reductase, NrfA Methods Enzymol 2011 496 399 422 21514473 10.1016/B978-0-12-386489-5.00016-6

[B223] Holden HM Thoden JB Raushel FM Carbamoyl phosphate synthetase: an amazing biochemical odyssey from substrate to product Cell Mol Life Sci 1999 56 507 522 10.1007/s000180050448 11212301 PMC11147029

[B224] Crandall JB Teece MA Urea is a dynamic pool of bioavailable nitrogen in coral reefs Coral Reefs 2012 31 207 214 10.1007/s00338-011-0836-1

[B225] Catmull J Yellowlees D Miller DJ NADP^+^-dependent glutamate dehydrogenase from *Acropora formosa*: purification and properties Mar Biol 1987 95 559 563 10.1007/BF00393099

[B226] Clode PL Saunders M Maker G Ludwig M Atkins CA Uric acid deposits in symbiotic marine algae Plant Cell Environ 2009 32 170 177 10.1111/j.1365-3040.2008.01909.x 19021889

[B227] Lancaster JR Jr Simulation of the diffusion and reaction of endogenously produced nitric oxide Proc Natl Acad Sci USA 1994 91 8137 8141 10.1073/pnas.91.17.8137 8058769 PMC44560

[B228] Trapido-Rosenthal H Zielke S Owen R Buxon L Boeing B Bhagooli R Archer J Increased zooxanthellae nitric oxide synthase activity is associated with coral bleaching Biol Bull 2005 208 3 6 10.2307/3593094 15713806

[B229] Bouchard JN Yamasaki H Heat stress stimulates nitric oxide production in *Symbiodinium microadriaticum*: a possible linkage between nitric oxide and the coral bleaching phenomenon Plant Cell Physiol 2008 49 641 652 10.1093/pcp/pcn037 18308760

[B230] Perez S Weis V Nitric oxide and cnidarians bleaching: an eviction notice mediates breakdown of a symbiosis J Exp Biol 2006 209 2804 2810 10.1242/jeb.02309 16809471

[B231] Safavi-Hemami H Young ND Doyle J Llewellyn L Klueter A Characterisation of nitric oxide synthase in three cnidarian-dinoflagellate symbioses PLoS One 2010 5 e10379 10.1371/journal.pone.0010379 20442851 PMC2861001

[B232] Dreyer J Schleicher M Tappe A Schilling K Kuner T Kusumawidijaja G Müller-Esterl W Oess S Kuner R Nitric oxide synthase (NOS)-interacting protein interacts with neuronal NOS and regulates its distribution and activity J Neurosci 2004 24 10454 10465 10.1523/JNEUROSCI.2265-04.2004 15548660 PMC6730309

[B233] Siebeck O Photoactivation and depth-dependent UV tolerance in reef coral in the Great Barrier Reef/Australia Naturwissenschaften 1981 68 426 428 10.1007/BF01079713

[B234] Reef R Dunn S Levy O Dove S Shemesh E Brickner I Leggat W Hoegh-Guldberg O Photoreactivation is the main repair pathway for UV-induced DNA damage in coral plenulae J Exp Biol 2009 212 2760 2766 10.1242/jeb.031286 19684208

[B235] Anderson S Zepp R Machula J Santavy D Hansen L Mueller E Indicators of UV exposure in corals and their relevance to global climate change and coral bleaching Hum Ecol Risk Assess 2001 7 1271 1782 10.1080/20018091094998

[B236] Torregiani JH Lesser MP The effects of short-term exposures to ultraviolet radiation in the Hawaiian coral *Montipora verrucosa* J Exp Mar Biol Ecol 2007 340 194 203 10.1016/j.jembe.2006.09.004

[B237] Baruch R Avishai N Rabinowitz C UV incites diverse levels of DNA breaks in different cellular compartments of a branching coral species J Exp Biol 2005 208 843 848 10.1242/jeb.01496 15755882

[B238] Hudson CL Ferrier MD Assessing ultraviolet radiation-induced DNA damage and repair in field-collected *Aiptasia pallida* using the comet assay Proceedings of the 11th International Coral Reef Symposium 2008 Florida 7 11

[B239] Nesa B Baird AH Harii S Yakovleva I Hidaka M Algal symbionts increase DNA damage in coral plenulae exposed to sunlight Zool Stud 2012 51 112 117

[B240] Vijayavel K Downs CA Ostrander GK Richmond RH Oxidative DNA damage induced by iron chloride in the larvae of the lace coral *Pocillopora damicornis* Comp Biochem Physiol C Toxicol Pharmacol 2012 155 275 280 10.1016/j.cbpc.2011.09.007 21963688

[B241] Lesser MP Farrell JH Exposure to solar radiation increases damage to both host tissues and algal symbionts of corals during thermal stress Coral Reefs 2004 23 367 377 10.1007/s00338-004-0392-z

[B242] Polato NR Vera JC Baums IB Gene discovery in the threatened elkhorn coral: 454 sequencing of the *Acropora palmata* transcriptome PLoS One 2011 6 e28634 10.1371/journal.pone.0028634 22216101 PMC3247206

[B243] Lindquist S Craig EA The heat-shock proteins Annu Rev Genet 1988 22 631 677 10.1146/annurev.ge.22.120188.003215 2853609

[B244] Åkerfelt M Morimoto RI Sistonen L Heat shock factors: Integrators of cell stress, development and lifespan Nat Rev Mol Cell Biol 2010 11 545 555 10.1038/nrm2938 20628411 PMC3402356

[B245] Bosch TCG Praetzel G The heat shock response in hydra: immunological relationship of hsp60, the major heat shock protein of *Hydra vulgaris*, to the ubiquitous hsp70 family Hydrobiologia 1991 216–217 513 51711538047

[B246] Choresh O Ron E Loya Y The 60-kDa hear shock protein (HSP60) of the sea anemone *Anemonia virdis*; a potential early warning system for environmental change Mar Biotechnol (NY) 2001 3 501 508 10.1007/s10126-001-0007-4 14961344

[B247] Bromage E Carpenter L Kaattari S Patterson M Quantification of coral heat shock proteins from individual coral polyps Mar Ecol Prog Ser 2009 376 123 132

[B248] Chow AR Ferrier-Pagès C Khalouei S Reynaud S Brown IR Increased light intensity induces heat shock protein Hsp60 in coral species Cell Stress Chaperones 2009 14 469 476 10.1007/s12192-009-0100-6 19214783 PMC2728280

[B249] Venn AA Quinn J Jones R Bodnar A P-glycoprotein (multi-xenobiotic resistance) and heat shock protein gene expression in the coral *Montastraea franksi* in response to environmental toxicant Aquat Toxicol 2009 93 188 195 10.1016/j.aquatox.2009.05.003 19501419

[B250] Nakamura M Morita M Kurihara H Mitarai S Expression of *hsp*70, *hsp*90 and *hsf*1 in the reef coral *Acropora digitifera* under prospective acidified conditions over the next several decades Biol Open 2012 1 75 81 10.1242/bio.2011036 23213399 PMC3507200

[B251] Hayes RL King CM Induction of 70-kD heat shock protein in scleractinian corals by elevated temperature: significance for coral bleaching Mol Mar Biol Biotechnol 1995 4 36 42 7749464

[B252] Fang L-S Huang S-P Lin K-L High temperature induces the synthesis of heat-shock proteins and the elevation on intracellular calcium in the coral *Acropora grandis* Coral Reefs 1997 16 127 131 10.1007/s003380050066

[B253] Carpenter LW Patterson MR Bromage ES Water flow influences the spatiotemporal distribution of heat shock protein 70 within colonies of the scleractinian coral *Montastrea annularis* (Ellis and Solander, 1786) following heat stress: Implications for coral bleaching J Exp Mar Biol Ecol 2010 387 52 59 10.1016/j.jembe.2010.02.019

[B254] Rosic NN Pernice M Dove S Dunn S Hoegh-Guldberg O Gene expression profiles of cytosolic heat shock proteins Hsp70 and Hsp90 from symbiotic dinoflagellates in response to thermal stress: possible implications for coral bleaching Cell Stress Chaperones 2011 16 69 80 10.1007/s12192-010-0222-x 20821176 PMC3024090

[B255] Roth MS Goericke R Deheyn DD Cold induces acute stress but heat is ultimately more deleterious for the reef-building coral *Acropora yongei* Sci Rep 2012 2 240 22355753 10.1038/srep00240 PMC3270498

[B256] Aravind L Anantharaman V Koonin EV Monophyly of class I aminoacyl tRNA synthase, USPA, ETFP, photolyase and the PP-ATPase nucleotide-binding domains: Implications for protein evolution in the RNA world Proteins 2002 48 1 14 10.1002/prot.10064 12012333

[B257] Kültz D Molecular and evolutionary basis of the cellular stress response Annu Rev Physiol 2005 67 225 257 10.1146/annurev.physiol.67.040403.103635 15709958

[B258] Kvint K Nachin L Diez A Nyström T The bacterial universal stress protein: functions and regulation Curr Opin Microbiol 2003 6 140 145 10.1016/S1369-5274(03)00025-0 12732303

[B259] Kerk D Bulgrien J Smith DW Gribskov M Arabidopsis proteins containing similarity to the universal stress protein domain of bacteria Plant Physiol 2003 131 1209 1219 10.1104/pp.102.016006 12644671 PMC166881

[B260] Forêt S Seneca F de Jong D Bieller A Hemmrich G Augustin R Hayward DC Ball EE Bosch TC Agata K Hassel M Miller DJ Phylogenomics reveals an anomalous distribution of USP genes in metazoans Mol Biol Evol 2011 28 153 161 10.1093/molbev/msq183 20660083

[B261] DeSalvo MK Voolstra CR Sunagawa S Schwartz JA Stillman JH Coffroth MA Szmant AM Medina M Differential gene expression during thermal stress and bleaching in the Caribbean coral *Montastraea faveolata* Mol Ecol 2008 17 3952 3971 10.1111/j.1365-294X.2008.03879.x 18662230

[B262] Voolstra CR Schnetzer J Penshkin L Randall CJ Szmant AM Medina M Effects of temperature on gene expression in embryos of the coral *Montastraea faveolata* BMC Genomics 2009 10 627 10.1186/1471-2164-10-627 20030803 PMC2807443

[B263] Sone H Akanuma H Fukuda T Oxygenomics in environmental stress Redox Rep 2010 15 98 114 10.1179/174329210X12650506623843 20594413 PMC7067327

[B264] Pronk TE Van Someren EP Stierum RH Ezendam J Pennings JLA Unraveling toxicological mechanisms and predicting toxicity classes with gene dysregulation networks J Appl Toxicol 2012 [Epub ahead of print] 10.1002/jat.2800 22886929

[B265] Hernández MP Sullivan WP Toft DO The assembly and intermolecular properties of the hsp70-Hop-hsp90 molecular complex J Biol Chem 2002 277 38294 38304 10.1074/jbc.M206566200 12161444

[B266] Song Y Masison DC Independent regulation of Hsp70 and Hsp90 chaperones by Hsp70/Hsp90-organising protein Sti (Hop1) J Biol Chem 2005 280 34178 34185 10.1074/jbc.M505420200 16100115 PMC1343460

[B267] Ellis RJ van der Vies SM Molecular chaperones Annu Rev Biochem 1991 60 321 347 10.1146/annurev.bi.60.070191.001541 1679318

[B268] Takayama S Reed JC Homma S Heat-shock proteins as regulators of apoptosis Oncogene 2003 2003 22 9041 9047 14663482 10.1038/sj.onc.1207114

[B269] Qiu XB Shao YM Miao S Wang L The diversity of the DnaJ/Hsp40 family, the crucial partners for Hsp70 chaperones Cell Mol Life Sci 2006 63 2560 2570 10.1007/s00018-006-6192-6 16952052 PMC11136209

[B270] Ke Q Costa M Hypoxia-inducible factor-1 (HIF-1) Mol Pharmacol 2006 70 1469 1480 10.1124/mol.106.027029 16887934

[B271] Rankin EB Biju MP Liu Q Unger TL Rha J Johnson RS Simon MC Keith B Haase VH Hypoxia-inducible factor-2 (HIF-2) regulates erythropoietin in vivo J Clin Invest 2007 117 1068 1077 10.1172/JCI30117 17404621 PMC1838939

[B272] Levy O Kaniewska P Alon S Eisenberg E Karako-Lampert S Bay LK Reef R Rodriguez-Lanetty M Miller DJ Hoegh-Guldberg O Complex diel cycles of gene expression in coral-algal symbiosis Science 2011 331 175 10.1126/science.1196419 21233378

[B273] Glickman MH Ciechanover A The ubiquitin-proteasome proteolytic pathway: destruction for the sake of construction Physiol Rev 2002 82 373 428 11917093 10.1152/physrev.00027.2001

[B274] Parsell DA Lindquist S The function of heat-shock proteins in stress tolerance: degradation and reactivation of damaged proteins Annu Rev Genet 1993 27 437 496 10.1146/annurev.ge.27.120193.002253 8122909

[B275] Imai J Yashiroda H Maruya M Yahara I Tanaka K Proteasomes and molecular chaperones: cellular machinery responsible for folding and destruction of unfolded proteins Cell Cycle 2003 2 585 590 14512774

[B276] Rosenzweig R Glickman MH Forging a proteasome α-ring with dedicated proteasome chaperones Nat Sruct Mol Biol 2008 15 218 220 10.1038/nsmb0308-218 18319735

[B277] Murata S Yashiroda H Tanaka K Molecular mechanisms of proteasome assembly Nat Rev Mol Cell Biol 2009 10 104 115 10.1038/nrm2630 19165213

[B278] Bügl H Fauman EB Staker BL Zheng F Kushner SR Saper MA Bardwell JC Jakob U RNA methylation under heat shock control Mol Cell 2000 6 349 360 10.1016/S1097-2765(00)00035-6 10983982

[B279] Caldas T Binet E Bouloc P Costa A Desgres J Richarme G The FtsJ/RrmJ heat shock protein of *Escherichia coli* is a 23S ribosomal RNA methyl transferase J Biol Chem 2000 275 16414 16419 10.1074/jbc.M001854200 10748051

[B280] Shimuta T Nakano K Yamaguchi Y Ozaki S Fujimitsu K Matsunaga C Noguchi K Emoto A Katayama T Novel heat shock protein HspQ stimulates degradation of mutant DnaA protein in *Escherichia coli* Genes Cells 2004 9 1151 1166 10.1111/j.1365-2443.2004.00800.x 15569148

[B281] Kitagawa M Wada C Yoshioka S Yura T Expression of ClpB, an analog of the ATP-dependent protease regulatory subunit in *Escherichia coli*, is controlled by a heat shock sigma factor (sigma 32) J Bacteriol 1991 173 4247 4253 1906060 10.1128/jb.173.14.4247-4253.1991 PMC208083

[B282] Squires C Squires CL The Clp proteins: proteolysis or molecular chaperones? J Bacteriol 1992 174 1081 1085 1735703 10.1128/jb.174.4.1081-1085.1992 PMC206400

[B283] Lupas AN Koretke KK Bioinformatic anatysis of ClpS, a protein module involved in prokaryotic and eukaryotic protein degradation J Struct Biol 2003 141 77 83 10.1016/S1047-8477(02)00582-8 12576022

[B284] Maillard RA Chistol G Sen M Righini M Tan J Kaiser CM Hodges C Martin A Bustamante C ClpX(P) generates mechanical force to unfold and translocate its protein substrates Cell 2011 145 459 469 10.1016/j.cell.2011.04.010 21529717 PMC3686100

[B285] Horwitz J Alpha-crystallin can function as a molecular chaperone Proc Natl Acad Sci U S A 1992 89 10449 10453 10.1073/pnas.89.21.10449 1438232 PMC50356

[B286] Rao PV Horwitz J Zigler JS α-Crystallin, a molecular chaperone, forms a stable complex with carbonic anhydrase upon heat denaturation Biochem Biophys Res Commun 1993 190 786 793 10.1006/bbrc.1993.1118 8094957

[B287] Carricart-Ganivet JP Cabanillas-Terán N Cruz-Ortega I Blanchon P Sensitivity of calcification to thermal stress varies among genera of massive reef-building corals PLoS One 2012 7 e32859 10.1371/journal.pone.0032859 22396797 PMC3291612

[B288] Taylor JL Wieczorek A Keyser AR Grover A Flinkstrom R Karls RK Bielefeldt-Ohmann H Dobos KM Izzo AA HspX-mediated protection against tuberculosis depends on the chaperoning of a mycobacterial molecule Immunol Cell Biol 2012 90 945 954 10.1038/icb.2012.34 22801575 PMC3511932

[B289] Santo Ede O Alves N Jr Dias GM Mazotto AM Vermelho A Vora GJ Wilson B Beltran VH Bourne DG Le Roux F Thompson FL Genomic and proteomic analysis of the pathogen *Vibrio coralliilyticus* reveals a diverse virulence repertoire ISME J 2011 5 1471 1483 10.1038/ismej.2011.19 21451583 PMC3160677

[B290] Kimes NE Grim CJ Johnson WR Hasan NA Tall BD Kothary MH Kiss H Munk AC Tapia R Green L Detter C Bruce DC Brettin TS Colwell RR Morris PJ Temperature regulation of virulence factors in the pathogen *Vibrio coralliilyticus* ISME J 2012 6 835 846 10.1038/ismej.2011.154 22158392 PMC3309362

[B291] Zhang T Kraus WL SIRT1-dependent regulation of chromatin and transcription: linking NAD^+^ metabolism and signaling to the control of cellular functions Biochim Biophys Acta 1804 2010 1666 1675 10.1016/j.bbapap.2009.10.022 PMC2886162 19879981

[B292] Liu TF Yoza BK El Gazzar M Vachharajani VT McCall CE NAD^+^-dependent SIRT1 deacetylase participates in epigenetic reprogramming during endotoxin tolerance J Biol Chem 2011 286 9856 9864 10.1074/jbc.M110.196790 21245135 PMC3058977

[B293] Katada S Imhof A Sassone-Corsi P Connecting threads: epigenetics and metabolism Cell 2012 148 24 28 10.1016/j.cell.2012.01.001 22265398

[B294] Lim JH Lee YM Chun YS Chen J Kim JE Park JW Sirtuin 1 modulates cellular responses to hypoxia by deacetylating hypoxia-inducible Factor 1α Mol Cell 2010 38 864 878 10.1016/j.molcel.2010.05.023 20620956

[B295] Lee JH Song MY Song EK Kim EK Moon WS Han MK Park JW Kwon KB Park BH Overexpression of SIRT1 protects pancreatic beta-cells against cytokine toxicity by suppressing the nuclear factor-kappaB signalling pathway Diabetes 2009 58 344 351 19008341 10.2337/db07-1795 PMC2628607

[B296] Wang F Nguyen M Qin FX Tong Q SIRT2 deacetylates FOXO3a in response to oxidative stress and caloric restriction Aging Cell 2007 6 505 514 10.1111/j.1474-9726.2007.00304.x 17521387

[B297] Shi T Wang F Stieren E Tong Q SIRT3, a mitochondrial sirtuin deacetylase, regulates mitochondrial function and thermogenesis in brown adipocytes J Biol Chem 2005 280 13560 13567 10.1074/jbc.M414670200 15653680

[B298] Nasrin N Wu X Fortier E Feng Y Baré OC Chen S Ren X Wu Z Streeper RS Bordone L SIRT4 regulates fatty acid oxidation and mitochondrial gene expression in liver and muscle cells J Biol Chem 2010 285 31995 32002 10.1074/jbc.M110.124164 20685656 PMC2952200

[B299] Du J Zhou Y Su X Yu JJ Khan S Jiang H Kim J Woo J Kim JH Choi BH He B Chen W Zhang S Cerione RA Auwerx J Hao Q Lin H Sirt5 is a NAD-dependent protein lysine demalonylase and desuccinylase Science 2011 334 806 809 10.1126/science.1207861 22076378 PMC3217313

[B300] Mostoslavsky R Chua KF Lombard DB Pang WW Fisher MR Gellon L Liu P Mostoslavsky G Franco S Murphy MM Mills KD Patel P Hsu JT Hong AL Ford E Cheng HL Kennedy C Nunez N Bronson R Frendewey D Auerbach W Valenzuela D Karow M Hottiger MO Hursting S Barrett JC Guarente L Mulligan R Demple B Yancopoulos GD Genomic instability and aging-like phenotype in the absence of mammalian SIRT6 Cell 2006 124 315 329 10.1016/j.cell.2005.11.044 16439206

[B301] Luo J Nikolaev AY Imai S Chen D Su F Shiloh A Guarente L Gu W Negative control of p53 by Sir2alpha promotes cell survival under stress Cell 2001 107 137 148 10.1016/S0092-8674(01)00524-4 11672522

[B302] Vakhrusheva O Smolka C Gajawada P Kostin S Boettger T Kubin T Braun T Bober E Sirt7 increases stress resistance of cardiomyocytes and prevent apoptosis and inflammatory cardiomyopathy in mice Circ Res 2008 102 703 710 10.1161/CIRCRESAHA.107.164558 18239138

[B303] O’Halloran TV Culotta VC Metallochaperones, an intracellular shuttle service for metal ions J Biol Chem 2000 275 25057 25060 10.1074/jbc.R000006200 10816601

[B304] Lin SJ Culotta VC The *ATX1* gene *of Saccharomyces cerevisiae* encodes a small metal homeostasis factor that protects cells against reactive oxygen toxicity Proc Natl Acad Sci U S A 1995 92 3784 3788 10.1073/pnas.92.9.3784 7731983 PMC42046

[B305] Culotta VC Klomp LWJ Strain J Casareno RLB Krems B Gitlin JD The copper chaperone for superoxide dismutase J Biol Chem 1997 272 23469 23472 10.1074/jbc.272.38.23469 9295278

[B306] Wong PC Waggoner D Subramaniam JR Tessarollo L Bartnikas TB Culotta VC Price DL Rothstein J Gitlin JD Copper chaperone for superoxide dismutase is essential to activate mammalian Cu/Zn superoxide dismutase Proc Natl Acad Sci USA 2000 97 2886 2891 10.1073/pnas.040461197 10694572 PMC16025

[B307] Shick JM Lesser MP Dunlap WC Stochaj WR Chalker BE Wu Won J Depth-dependent responses to solar ultraviolet radiation and oxidative stress in the zooxanthellate coral *Acropora microphthalma* Mar Biol 1995 122 41 51 10.1007/BF00349276

[B308] Lesser MP Stochaj WR Tapley DW Shick JM Bleaching in coral-reef anthozoans: effects of irradiance, ultraviolet radiation, and temperature on the activities of protective enzymes against active oxygen Coral Reefs 1990 8 225 232 10.1007/BF00265015

[B309] Crowe JH Carpenter JF Crowe LM The role of vitrification in anhydrobiosis Annu Rev Physiol 1998 60 73 103 10.1146/annurev.physiol.60.1.73 9558455

[B310] Sola-Penna M Meyer-Fernandes JR Stabilization against thermal inactivation promoted by sugars on enzyme structure and function: Why is trehalose more effective than other sugars? Arch Biochem Biophys 1998 360 10 14 10.1006/abbi.1998.0906 9826423

[B311] Veron JEN Corals of Australia and the Indo-Pacific 1993 Singapore: University of Hawaii Press 644

[B312] Shick JM Shima A, Ichihashi M, Fujiwara Y, Takebe H Solar UV and oxidative stress in algal-animal symbioses Frontiers of Photobiology 1993 Amsterdam: Elsevier Science Publishers 561 564

[B313] Turrens JF Mitochondrial formation of reactive oxygen species J Physiol 2003 552 335 344 10.1113/jphysiol.2003.049478 14561818 PMC2343396

[B314] Lesser MP Oxidative stress causes coral bleaching during exposure to elevated temperatures Coral Reefs 1997 16 187 192 10.1007/s003380050073

[B315] Brown BE Downs CA Dunn RP Gibb SW Exploring the basis of thermotolerance in the reef coral *Goniastrea aspera* Mar Ecol Prog Ser 2002 242 119 120

[B316] Halliwell B Reactive species and antioxidants. Redox biology is a fundamental theme of aerobic life Plant Physiol 2006 141 312 322 10.1104/pp.106.077073 16760481 PMC1475431

[B317] Liñán-Cabello MA Lesser MP Flores-Ramírez LA Zenteno-Savín T Reyes-Bonilla H Abele D, Vázquez-Medina JP, Zenteno-Savín T Oxidative stress in coral-photobiont communities Oxidative Stress in Aquatic Ecosystems 2011 Chichester: Wiley-Blackwell 127 138

[B318] Jönsson TJ Lowther WT The peroxiredoxin repair proteins Subcell Biochem 2007 44 115 141 10.1007/978-1-4020-6051-9_6 18084892 PMC2391273

[B319] Cheng Z Zhang J Ballou DP Williams CH Jr Reactivity of thioredoxin as a protein thiol-disulfide oxidoreductase Chem Rev 2011 111 5768 5783 10.1021/cr100006x 21793530 PMC3212873

[B320] Arnér ESJ Holmgren A Physiological function of thioredoxin and thioredoxin reductase Eur J Biochem 2000 267 6102 6109 10.1046/j.1432-1327.2000.01701.x 11012661

[B321] Ainsworth TD Hoegh-Guldberg O Cellular processes of bleaching in the Mediterranean coral *Oculina patagonica* Coral Reefs 2008 27 593 597 10.1007/s00338-008-0355-x 18059488

[B322] Kenkel CD Aglyamova G Alamaru A Bhagooli R Capper R Cunning R De Villers A Haslun JA Hédouin L Keshavmurthy S Kuehl KA Mahmoud H McGinty ES Montoya-Maya PH Palmer CV Pantile R Sánchez JA Schils T Silverstein RN Squiers LB Tang PC Goulet TL Matz MV Development of gene expression markers of acute heat-light stress in reef-building corals of the genus *Porites* PLoS One 2011 6 e26914 10.1371/journal.pone.0026914 22046408 PMC3202587

[B323] Wang C Youle RJ The role of mitochondria in apoptosis Annu Rev Genet 2009 43 95 118 10.1146/annurev-genet-102108-134850 19659442 PMC4762029

[B324] Richier S Sabourault C Courtiade J Zucchini N Allemand Furla P Oxidative stress and apoptotic events during thermal stress in the sea anemone, *Anemone viridis* FEBS J 2006 273 4186 4198 10.1111/j.1742-4658.2006.05414.x 16907933

[B325] Lasi M Pauly B Schmidt N Cikala M Stiening B Käsbauer T Zenner G Popp T Wagner A Knapp RT Huber AH Grunert M Söding J David CN Böttger A The molecular cell death machinery in the simple cnidarian *Hydra* includes an expanded caspase family and pro- and anti-apoptotic Bcl-2 proteins Cell Res 2010 20 812 825 10.1038/cr.2010.66 20479784

[B326] Baker AC Starger CJ McClanahan TR Glynn PW Coral reefs: corals’ adaptive response to climate change Nature 2004 430 741 10.1038/430741a 15306799

[B327] McClanahan T The relationship between bleaching and mortality of common corals Mar Biol 2004 144 1239 1245 10.1007/s00227-003-1271-9

[B328] Ainsworth TD Wasmund K Ukani L Seneca F Yellowlees D Miller D Leggat W Defining the tipping point. A complex cellular life/death balance in corals in response to stress Sci Rep 2011 1 160 22355675 10.1038/srep00160 PMC3240979

[B329] Tchernov D Kvitt H Haramaty L Bibby TS Gorbunov MY Rosenfeld H Falkowski PG Apoptosis and the selective survival of host animals following thermal bleaching in zooxanthellate corals Proc Natl Acad Sci U S A 2011 108 9905 9909 10.1073/pnas.1106924108 21636790 PMC3116386

[B330] Pernice M Dunn SR Miard T Dufour S Dove S Hoegh-Guldberg O Regulation of apoptotic mediators reveals dynamic responses to thermal stress in the reef building coral *Acropora millepora* PLoS One 2011 6 e16095 10.1371/journal.pone.0016095 21283671 PMC3025915

[B331] David CN Schmidt N Schade M Pauly B Alexabdrova O Böttger A Hydra and the evolution of apoptosis Integr Comp Biol 2005 45 631 638 10.1093/icb/45.4.631 21676810

[B332] Zmasek CM Zhang Q Ye Y Godzik A Surprising complexity of the ancestral apoptosis network Genome Biol 2007 8 R226 10.1186/gb-2007-8-10-r226 17958905 PMC2246300

[B333] Siepp S Wittig K Stiening B Böttger A Leitz T Morphogenesis in *Hydractina echinata* (Cnidaria) is capase-dependent Int J Dev Biol 2006 50 63 70 10.1387/ijdb.052012ss 16323079

[B334] Dunn SR Weis VM Apoptosis as a post-phagocytic mechanism in a coral-dinoflagellate mutualism Environ Microbiol 2009 11 268 276 10.1111/j.1462-2920.2008.01774.x 19125818

[B335] Adrian C Brumatti G Martin SJ Apoptosomes: protease activation platforms to die for Trends Biochem Sci 2006 31 243 247 10.1016/j.tibs.2006.03.004 16595176

[B336] Yang X Khosravi-Far R Chang HY Baltimore D Daxx, a novel Fas-binding protein that activates JNK and apoptosis Cell 1997 89 1067 1076 10.1016/S0092-8674(00)80294-9 9215629 PMC2989411

[B337] Cohen O Inbal B Kissil JL Raveh T Berissi H Spivak-Kroizaman T Feinstein E Kimchi A DAP-kinase participates in TNF-α- and Fas-induced apoptosis and its function requires the death domain J Cell Biol 1999 146 141 148 10402466 10.1083/jcb.146.1.141 PMC2199731

[B338] Lee JH Rho SB Chun T Programmed cell death 6 (PDCD6) protein interacts with death associated protein kinase 1 (DAPk1): additive effect on apoptosis via caspase-3 dependent pathway Biotechnol Lett 2005 27 1011 1015 10.1007/s10529-005-7869-x 16132846

[B339] Kaiser WJ Upton JW Long AB Livingstone-Rosanoff D Daley-Bauer LP Hakem R Caspary T Mocarski ES RIP3 mediates the embryonic lethality of caspase-8 deficient mice Nature 2011 471 368 372 10.1038/nature09857 21368762 PMC3060292

[B340] Krumschnabel G Sohm B Bock F Manzl C Villunger A The enigma of caspase-2: the layman’s view Cell Death Differ 2009 16 195 207 10.1038/cdd.2008.170 19023332 PMC3272397

[B341] Khorchid A Ikura M How calpain is activated by calcium Nat Struct Biol 2002 9 239 341 10.1038/nsb0402-239 11914728

[B342] Janssens S Timel A Lippens T Tschopp J PIDD mediates NF-κB activation response to DNA damage Cell 2005 123 1079 1092 10.1016/j.cell.2005.09.036 16360037

[B343] Wolenski FS Garbati MR Lubinski TL Traylor-Knowles N Dresselhaus E Stefanik DJ Goucher H Finnerty JR Gilmore TD Characterisation of the core elements of the NF-κB signalling pathway of the sea anemone *Nematostella vectensis* Mol Cell Biol 2011 31 1076 1087 10.1128/MCB.00927-10 21189285 PMC3067825

[B344] Dunn SR Phillips WS Spatafora JW Green DR Wies VM Highly conserved caspase and Bcl-2 homologues from the sea anemone *Aptaisia pallida*: lower metazoans for the study of apoptosis evolution J Mol Evol 2006 63 95 107 10.1007/s00239-005-0236-7 16770683

[B345] Knowlton N Rohwer F Multispecies microbial mutualisms on coral reefs: the host as a habitat Am Nat 2003 162 Suppl S51 S62 14583857 10.1086/378684

[B346] Littman RA Willis BL Pfeffer C Bourne DG Diversities of coral-associated bacteria differ with location, but not species, for three acroporid corals on the Great Barrier Reef FEMS Microbiol Ecol 2009 68 152 153 10.1111/j.1574-6941.2009.00666.x 19302548

[B347] Rohwer F Seguritan V Azam F Knowlton N Diversity and distribution of coral-associated bacteria Mar Ecol Prog Ser 2002 243 1 10

[B348] Sogin ML Morrison HG Huber JA Mark Welch D Huse SM Neal PR Arrieta JM Herndl GJ Microbial diversity in the deep sea and the unexplored “rare biosphere” Proc Natl Acad Sci U S A 2006 103 12115 12120 10.1073/pnas.0605127103 16880384 PMC1524930

[B349] Sunagawa S DeSalvo MK Voolstra CR Reyes-Bermudez A Medina M Identification and gene expression analysis of a taxonomically restricted cysteine-rich protein family in reef-building corals PLoS One 2009 4 e4865 10.1371/journal.pone.0004865 19283069 PMC2652719

[B350] Rosenberg E Koren O Reshef L Efrony R Zilber-Rosenberg I The role of microorganisms in coral health, disease, and evolution Nat Rev Microbiol 2007 5 355 362 10.1038/nrmicro1635 17384666

[B351] Wild C Huettel M Klueter A Kremb SG Rasheed MY Jørgensen BB Coral mucus formation functions as an energy carrier and particle trap in the reef ecosystem Nature 2004 428 66 70 10.1038/nature02344 14999280

[B352] Garren M Azam F Corals shed bacteria as a potential mechanism of resilience to organic matter enrichment ISME J 2012 6 1159 1165 10.1038/ismej.2011.180 22189494 PMC3358026

[B353] Bourne DG Garren M Work TM Rosenberg E Smith GW Harvell CD Microbial disease and the coral holobiont Trends Microbiol 2009 17 554 562 10.1016/j.tim.2009.09.004 19822428

[B354] Reshef L Koren O Loya Y Zilber-Rosenberg I Rosenberg E The coral probiotic hypothesis Environ Microbiol 2006 8 2068 2073 10.1111/j.1462-2920.2006.01148.x 17107548

[B355] Vollmer SV Kline DI Natural disease resistance in threatened staghorn corals PLoS One 2008 3 e3718 10.1371/journal.pone.0003718 19005565 PMC2579483

[B356] Carroll SP Hendry AP Reznick DN Fox CW Evolution on ecological time scales Funct Ecol 2007 21 387 393 10.1111/j.1365-2435.2007.01289.x

[B357] van Oppen MJH Souter P Howells EJ Heyward A Berkelmans R Novel genetic diversity through somatic mutations: fuel for adaptation of reef corals? Diversity 2011 3 405 423 10.3390/d3030405

[B358] Mydlarz LD McGinty ES Hravell CD What are the physiological and immunological responses of coral to climate warming and disease? J Exp Biol 2010 213 934 945 10.1242/jeb.037580 20190118

[B359] Hamada M Shoguchi E Shinzato C Kawashima T Miller DJ Satoh N The complex NOD-like receptor repertoire of the coral *Acropora digitifera* includes novel domain combinations Mol Biol Evol 2013 30 167 176 10.1093/molbev/mss213 22936719

[B360] Boyes DC Nam J Dangl JL The *Arabidopsis thaliana* RPM1 disease resistance gene product is a peripheral plasma membrane protein that is degraded coincident with the hypersensitive response Proc Natl Acad Sci U S A 1998 95 15849 15854 10.1073/pnas.95.26.15849 9861059 PMC28133

[B361] Axtell MJ Stakawicz BJ Initiation of RPS2-specified disease resistance in *Arabidopsis* is coupled to the AvrRpt2-directed elimination of RIN4 Cell 2003 112 369 377 10.1016/S0092-8674(03)00036-9 12581526

[B362] Edreva A Pathogenesis-related proteins: research progress in the last 15 years Gen Appl Plant Physiol 2005 31 105 124

[B363] Palmer CV Mydlarz LD Willis BL Evidence of an inflammatory-like response in non-normally pigmented tissues of two scleractinian corals Proc Biol Sci 2008 275 2687 2693 10.1098/rspb.2008.0335 18700208 PMC2605814

[B364] Mydlarz LD Couch CS Weil E Smith G Harvell CD Immune defenses of healthy, bleached and diseased *Montastraea faveolata* during a natural bleaching event Dis Aquat Organ 2009 87 67 78 20095242 10.3354/dao02088

[B365] Palmer C Bythwll JC Willis BL A comparative study of phenoloxidase activity in diseased and bleached colonies of the coral *Acropora millepora* Dev Comp Immunol 2011 35 1098 1101 10.1016/j.dci.2011.04.001 21527282

[B366] Cascales E Christie PJ The versatile bacterial type IV secretion systems Nat Rev Microbiol 2003 1 137 149 10.1038/nrmicro753 15035043 PMC3873781

[B367] Juhas M Crook DW Hood DW Type IV secretion systems: tools of bacterial gene transfer and virulence Cell Microbiol 2008 10 2377 2386 10.1111/j.1462-5822.2008.01187.x 18549454 PMC2688673

[B368] Technau U Rudd S Maxwell P Gordon PM Saina M Grasso LC Hayward DC Sensen CW Saint R Holstein TW Ball EE Miller DJ Maintenance of ancestral complexity and non-metazoan genes in two basal cnidarians Trends Genet 2005 21 633 639 10.1016/j.tig.2005.09.007 16226338

[B369] Khalturin K Hemmeich G Fraune S Augustin R Bosch TC More than just orphans: are taxonomically-restricted genes important in evolution? Trends Genet 2009 25 404 413 10.1016/j.tig.2009.07.006 19716618

[B370] Forêt S Knack B Houliston E Momose T Manuel M Quéinnec E Hayward DC Ball EE Miller DJ New tricks with old genes: the genetic basis of novel cnidarian traits Trends Genet 2010 26 154 158 10.1016/j.tig.2010.01.003 20129693

[B371] Bergh O Børsheim KY Bratbak G Heldal M High abundance of viruses found in aquatic environments Nature 1989 340 467 468 10.1038/340467a0 2755508

[B372] Børsheim KY Bratbak G Heldal M Enumeration and biomass estimation of planktonic bacteria and viruses by transmission electron microscopy Appl Environ Microbiol 1990 56 352 356 2306088 10.1128/aem.56.2.352-356.1990 PMC183343

[B373] Breitbart M Salamon P Andressen B Mahaffy JM Segall AM Mead D Azam F Rohwer F Genomic analysis of uncultured marine viral communities Proc Natl Acad Sci U S A 2002 99 14250 14255 10.1073/pnas.202488399 12384570 PMC137870

[B374] Williamson SJ Rusch DB Yooseph S Halpern AL Heidelberg KB Glass JI Andrews-Pfannkoch C Fadrosh D Miller CS Sutton G Frazier M Venter JC The Sorcerer II Global Ocean Sampling Expedition: metagenomic characterization of viruses within aquatic microbial samples PLoS One 2008 3 e1456 10.1371/journal.pone.0001456 18213365 PMC2186209

[B375] Bouvy M Combe M Bettarel Y Dupuy C Rochelle-Newall E Charpy L Uncoupled viral and bacterial distributions in coral reef waters of the Tuamotu Archipelago (French Polynesia) Mar Pollut Bull 2012 65 506 515 10.1016/j.marpolbul.2012.01.001 22284701

[B376] Dinsdale EA Pantos O Smriga S Edwards RA Angly F Wegley L Hatay M Hall D Brown E Haynes M Krause L Sala E Sandin SA Thurber RV Willis BL Azam F Knowlton N Rohwer F Microbial ecology of four coral atolls in the Northern Line Islands PLoS One 2008 3 e1584 10.1371/journal.pone.0001584 18301735 PMC2253183

[B377] Seymour JR Patten N Bourne DG Mitchell JG Spatial dynamic of virus-like particles and heterotrophic bacteria within a shallow coral reef system Mar Ecol Prog Ser 2005 288 1 8

[B378] Patten NL Seymour JR Mitchell JG Flow cytometric analysis of virus-like particles and heterotrophic bacteria within coral-associated reef water J Mar Biol Assoc UK 2006 86 563 566 10.1017/S0025315406013476

[B379] Leruste A Bouvier T Bettarel Y Enumerating viruses in coral mucus Appl Environ Microbiol 2012 78 6377 6379 10.1128/AEM.01141-12 22729548 PMC3416620

[B380] Davy JE Patten NL Morphological diversity of virus-like particles within the surface microlayer of scleractinian corals Aquat Microb Ecol 2007 47 37 44

[B381] Patten NL Harrison PL Mitchell JG Prevalence of virus-like particles within the staghorn scleractinian coral (*Acropora muricata*) from the Great Barrier Reef Coral Reefs 2008 27 569 580 10.1007/s00338-008-0356-9

[B382] Wilson WH Francis I Ryan K Davy SK Temperature induction of viruses in symbiotic dinoflagellates Aquat Microb Ecol 2001 25 99 102

[B383] Wilson WH Dale AL Davy JE Davy SK An enemy within? Observation of virus-like particles in reef corals Coral Reefs 2005 24 145 148 10.1007/s00338-004-0448-0

[B384] Davy SK Burchett SG Dale AL Davies P Davy JE Muncke C Hoegh-Guldberg O Wilson WH Viruses: agents of coral disease? Dis Aquat Org 2006 69 101 110 16703772 10.3354/dao069101

[B385] Correa AM Welsh RM Vega Thurber RL Unique nucleocytoplasmic dsDNA and +ssRNA viruses are associated with dinoflagellate endosymbionts of corals ISME J 2013 7 13 27 10.1038/ismej.2012.75 22791238 PMC3526182

[B386] Marhaver KL Edwards RA Rohwer F Viral communities associated with health and bleached corals Environ Microbiol 2008 10 2277 2286 10.1111/j.1462-2920.2008.01652.x 18479440 PMC2702503

[B387] Vega Thurber RL Barott KL Hall D Liu H Rodriguez-Mueller B Desnues C Edwards RA Haynes M Angly FE Wegley L Rohwer FL Metagenomic analysis indicates that stressors induce production of herpes-like viruses in the coral *Porites compressa* Proc Natl Acad Sci U S A 2008 105 18413 18418 10.1073/pnas.0808985105 19017800 PMC2584576

[B388] Bettarel Y Thuy NT Huy TQ Hoang PK Bouvier T Observation of virus-like particles in thin sections of the bleaching scleractinian coral *Acropora cytherea* J Mar Biol Assoc UK 2013 93 909 912 10.1017/S0025315411002062

[B389] Wilson WH Hurst CJ Coral viruses Studies in Viral Ecology, Volume 2. Animal host systems 2011 Hoboken: Wiley 143 152

[B390] Kuznetsov SG Bosch TC Self/nonself recognition in Cnidaria: contact of allergenic tissue does not result in elimination of nonself cells in *Hydra vulgaris* Zool (Jena) 2003 106 109 116 10.1078/0944-2006-00105 16351896

[B391] Rinkevich B Allorecognition and xenorecognition in reef corals: a decade of interactions Hydrobiologia 2004 530–531 443 450

[B392] Rinkevich B Neglected biological features in cnidarians self-nonself recognition Adv Exp Med Biol 2012 738 46 59 10.1007/978-1-4614-1680-7_4 22399373

[B393] Bosch TCG Heine H The path less explored: innate immune reactions in cnidarians Nucleic Acids and Molecular Biology. Volume 21. Innate Immunity of Plants, Animals and Humans 2008 Heidelberg: Springer-Verlag Berlin 27 42

[B394] Wood-Charlson EM Hollingsworth LL Krupp DA Weis VM Lectin/glycan interactions play a role in recognition in a coral/dinoflagellate symbiosis Cell Microbiol 2006 8 1985 1993 10.1111/j.1462-5822.2006.00765.x 16879456

[B395] Hemmrich G Miller DJ Bosch TCG The evolution of immunity: a low-life perspective Trends Immunol 2007 28 449 454 10.1016/j.it.2007.08.003 17855167

[B396] Miller DJ Hemmrich G Ball EE Hayward DC Khalturin K Funayama N Agata K Bosch TC The innate immune repertoire in Cnidaria - ancestral complexity and stochastic gene loss Genome Biol 2007 8 R59 10.1186/gb-2007-8-4-r59 17437634 PMC1896004

[B397] Kvennefors ECE Leggat W Hoegh-Guldberg O Degnan BM Barnes AC An ancient and variable mannose-binding lectin from the coral *Acropora millepora* binds both pathogens and symbionts Dev Comp Immunol 2008 32 1582 1592 10.1016/j.dci.2008.05.010 18599120

[B398] Bosch TCG Augustin R Anton-Erxleben F Fraune S Hemmrich G Zill H Rosenstiel P Jacobs G Schreiber S Leippe M Stanisak M Grötzinger J Jung S Podschun R Bartels J Harder J Schröder JM Uncovering the evolutionary history of innate immunity: the simple metazoan *Hydra* uses epithelial cells for host defence Dev Comp Immunol 2009 33 559 569 10.1016/j.dci.2008.10.004 19013190

[B399] Dunn SR Immunorecognition and immunoreceptors in the Cnidaria Invertebrate Surviv J 2009 6 7 14

[B400] Rosenstiel P Philipp EER Schreiber S Bosch TCG Evolution and function of innate immune receptors - insights from marine invertebrates J Innate Immun 2009 1 291 300 10.1159/000211193 20375587

[B401] Augustin R Bosch TCG Cnidarian immunity: a tale of two barriers Adv Exp Med Biol 2010 708 1 16 10.1007/978-1-4419-8059-5_1 21528690

[B402] Mydlarz LD Jones LE Harvell CD Innate immunity, environmental drivers, and disease ecology of marine and freshwater invertebrates Annu Rev Ecol Evol Syst 2006 37 251 288 10.1146/annurev.ecolsys.37.091305.110103

[B403] Widner WR Wickner RB Evidence that the SKI antiviral system of *Saccharomyces cerevisiae* acts by blocking expression of viral mRNA Mol Cell Biol 1993 13 4331 4341 8321235 10.1128/mcb.13.7.4331-4341.1993 PMC359991

[B404] Brown JT Bai X Johnson AW The yeast antiviral proteins Ski2p, Ski3p and Ski8p exist as a complex *in vivo* RNA 2000 6 449 457 10.1017/S1355838200991787 10744028 PMC1369926

[B405] Houseley J Tollervey D The many pathways of RNA degradation Cell 2009 136 763 776 10.1016/j.cell.2009.01.019 19239894

[B406] Arraiano CM Matos RG Barbas A RNase II: The finer details of the *modus operandi* of a molecular killer RNA Biol 2010 7 276 281 10.4161/rna.7.3.11490 20484980

[B407] Samuel CE Antiviral actions of interferon. Interferon-regulated proteins and their surprisingly selective antiviral activities Virology 1991 183 1 11 10.1016/0042-6822(91)90112-O 1711253

[B408] Haller O Kochs G Webe F The interferon response circuit: Induction and suppression by pathogenic viruses Virology 2006 344 119 130 10.1016/j.virol.2005.09.024 16364743 PMC7125643

[B409] Fensterl V Sen GC Interferons and viral infections Biofactors 2009 35 14 20 10.1002/biof.6 19319841

[B410] Taniguchi T Ogasawara K Takaoka A Tanaka N IRF family of transcription factors as regulators of host defense Annu Rev Immunol 2001 19 623 655 10.1146/annurev.immunol.19.1.623 11244049

[B411] Dornan D Eckert M Wallace M Shimizu H Ramsay E Hupp TR Ball KL Interferon regulatory factor 1 binding to p300 stimulates DNA-dependent acetylation of p53 Mol Cell Biol 2004 24 10083 10098 10.1128/MCB.24.22.10083-10098.2004 15509808 PMC525491

[B412] Salkowski CA Barber SA Detore GR Vogel SN Differential dysregulation of nitric oxide production in macrophages with targeted disruptions in INF regulatory factor-1 and -2 genes J Immunol 1996 156 3107 3110 8617930

[B413] Weisz A Marx P Sharf R Appella E Driggers PH Ozato K Levi BZ Human interferon consensus sequence binding protein is a negative regulator of enhancer elements common to interferon-inducible genes J Biol Chem 1992 267 25589 25596 1460054

[B414] Espert L Degols G Gongora C Blondel D Williams BR Silverman RH Mechti N ISG20, a new interferon-induced RNase specific for single-stranded RNA, defines an alternative antiviral pathway against RNA genomic viruses J Biol Chem 2003 278 16151 16158 10.1074/jbc.M209628200 12594219

[B415] Taylor GA Jeffers M Largaespada DA Jenkins NA Copeland NG Vande Woude GF Identification of a novel GTPase, the inducibly expressed GTPase, that accumulates in response to interferon γ J Biol Chem 1996 271 20399 20405 10.1074/jbc.271.34.20399 8702776

[B416] Haller O Staeheli P Kochs G Interferon-induced Mx proteins in antiviral host defense Biochimie 2007 89 812 818 10.1016/j.biochi.2007.04.015 17570575

[B417] Pichlmair A Schulz O Tan CP Rehwinkel J Kato H Takeuchi O Akira S Way M Schiavo G Reise Sousa C Activation of MDA5 requires higher-order RNA structures generated during virus infection J Virol 2009 83 10761 10769 10.1128/JVI.00770-09 19656871 PMC2753146

[B418] Deblandre GA Marinx OP Evans SS Majjaj S Leo O Caput D Huez GA Wathelet MG Expression cloning of an interferon-inducible 17-kDa membrane protein implicated in the control of cell growth J Biol Chem 1995 270 23860 23866 10.1074/jbc.270.40.23860 7559564

[B419] Farrar MA Schreiber RD The molecular cell biology of interferon-gamma and its receptor Annu Rev Immunol 1993 11 571 611 10.1146/annurev.iy.11.040193.003035 8476573

[B420] Blasius AL Giurisato E Cella M Schreiber RD Shaw AS Colonna M Bone marrow stromal cell antigen is a specific marker of type 1 IFN-producing cells in the native mouse, but a promiscuous cell surface antigen following IFN stimulation J Immunol 2006 177 3260 3265 16920966 10.4049/jimmunol.177.5.3260

[B421] Seth RB Sun L Ea CK Chen ZJ Indentification and characterization of MAVS, a mitochondrial antiviral signaling protein that activates NF-kappaB and IRF3 Cell 2005 122 669 682 10.1016/j.cell.2005.08.012 16125763

[B422] Crook NE Clem RJ Miller LK An apoptosis-inhibiting baculovirus gene with a zinc finger-like motif J Virol 1993 67 2168 2174 8445726 10.1128/jvi.67.4.2168-2174.1993 PMC240327

[B423] Yang YL Li XM The IAP family: endogenous caspase inhibitors with multiple biological activities Cell Res 2000 10 169 177 10.1038/sj.cr.7290046 11032169

[B424] Spear PG Longnecker R Herpesvirus entry: an update J Virol 2003 77 10179 10185 10.1128/JVI.77.19.10179-10185.2003 12970403 PMC228481

[B425] Nemerow GR Wolfert R McNaughton ME Cooper NR Identification and characterization of the Epstein-Barr virus receptor on human B lymphocytes and its relationship to the C3d component receptor (CR2) J Virol 1985 55 347 351 2410629 10.1128/jvi.55.2.347-351.1985 PMC254939

[B426] Wu FK Garcia JA Harrich D Gaynor RB Purification of the human immunodeficiency virus type 1 enhancer and TAR binding proteins EBP-1 and UBP-1 EMBO J 1988 7 2117 2130 3138113 10.1002/j.1460-2075.1988.tb03051.x PMC454507

[B427] Lu Y Qian XY Krug RM The influenza virus NS1 protein: a novel inhibitor of pre-mRNA splicing Genes Dev 1994 8 1817 1828 10.1101/gad.8.15.1817 7958859

[B428] Hale BG Randall RE Ortín J Jackson D The multifunctional NS1 protein of influenza A viruses J Gen Virol 2008 89 2359 2376 10.1099/vir.0.2008/004606-0 18796704

[B429] Freundlich M Ramani N Mathew E Sirko A Tsui P The role of intergration host factor in gene expression in *Escherichia coli* Mol Microbiol 1992 6 2557 2563 10.1111/j.1365-2958.1992.tb01432.x 1447969

[B430] Tailor CS Willett BJ Kabat D A putative cell surface receptor for anemia-inducing feline leukemia virus subgroup C is a member of a transporter superfamily J Virol 1999 73 6500 6505 10400745 10.1128/jvi.73.8.6500-6505.1999 PMC112732

[B431] Bard-Chapeau EA Jeyakani J Kok CH Muller J Chua BQ Gunaratne J Batagov A Jenjaroenpun P Kuznetsov VA Wei CL D’Andrea RJ Bourque G Jenkins NA Copeland NG Ecotopic viral integration site 1 (EVI1) regulates multiple cellular processes important for cancer and is a synergistic partner for FOS protein in invasive tumors Proc Natl Acad Sci U S A 2012 109 2168 2173 10.1073/pnas.1119229109 22308434 PMC3277513

[B432] Turk T Kem WR The phylum Cnidaria and investigations of its toxins and venoms until 1990 Toxicon 2009 54 1031 1037 10.1016/j.toxicon.2009.06.031 19576920

[B433] Anderluh G Macek P Cytolytic peptide and protein toxins from sea anemones (Anthozoa: Actiniaria) Toxicon 2002 40 111 124 10.1016/S0041-0101(01)00191-X 11689232

[B434] Frazão B Vasconcelos V Antunes A Sea anemone (Cnidaria, Anthozoa, Actiniaria) toxins: an overview Mar Drugs 2012 10 1812 1851 10.3390/md10081812 23015776 PMC3447340

[B435] Weston AJ Chung R Dunlap WC Morandini AC Marques AC Moura-da-Silva AM Ward M Padilla G da Silva LF Andreakis N Long PF Proteomic characterisation of toxins isolated from nematocysts of the South Atlantic jellyfish *Olindias sambaquiensis* Toxicon 2013 10.1016/j.toxicon.2013.05.002 23688393

[B436] Brinkman DL Burnell JN Biochemical and molecular characterisation of cubozoan protein toxins Toxicon 2009 54 1162 1173 10.1016/j.toxicon.2009.02.006 19232527

[B437] Brinkman DL Aziz A Loukas A Potriquet J Seymour J Mulvenna J Venom proteome of the Box Jellyfish *Chironex fleckeri* PLoS One 2012 7 e47866 10.1371/journal.pone.0047866 23236347 PMC3517583

[B438] Leppla SH Anthrax toxin edema factor: a bacterial adenylate cyclase that increases cyclic AMP concentrations of eukaryotic cells Proc Natl Acad Sci USA 1982 79 3162 3166 10.1073/pnas.79.10.3162 6285339 PMC346374

[B439] Tang WJ Guo Q The andenylyl cyclase activity of anthrax edema factor Mol Aspt Med 2009 30 423 430 10.1016/j.mam.2009.06.001 PMC2783455 19560485

[B440] Van Delden C Iglewski BH Cell-to-cell signaling and *Pseudomonas aeruginos*a infections Emerg Infect Dis 1998 4 551 560 10.3201/eid0404.980405 9866731 PMC2640238

[B441] Yates SP Merrill AR Elucidation of eukaryotic elongation factor-2 contact sites within the catalytic domain of *Pseudomonas aeruginosa* exotoxin A Biochem J 2004 379 563 572 10.1042/BJ20031731 14733615 PMC1224111

[B442] Hinnebusch BJ Rudolph AE Cherepanov P Dixon JE Schwan TG Forsberg A Role of Yersinia murine toxin in survival of *Yersinia pestis* in the midgut of the flea vector Science 2002 296 733 735 10.1126/science.1069972 11976454

[B443] Fagerlund A Lindbäck T Storset AK Granum PE Hardy SP *Bacillus cereus* Nhe is a pore-forming toxin with structural and functional properties similar to the ClyA (HlyE, SheA) family of haemolysins, able to induce osmotic lysis in epithelia Microbiology 2008 154 693 704 10.1099/mic.0.2007/014134-0 18310016

[B444] Marrack P Kappler J The staphylococcal enterotoxins and their relatives Science 1990 248 705 711 10.1126/science.2185544 2185544

[B445] Voth DE Ballard JD *Clostridium difficile* toxins: mechanism of action and role in disease Clin Microbiol Rev 2005 18 247 263 10.1128/CMR.18.2.247-263.2005 15831824 PMC1082799

[B446] Di Pierro M Lu R Uzzau S Wang W Margaretten K Pazzani C Maimone F Fasano A *Zonula occludens* toxin structure-function analysis. Identification of the fragment biologically active on tight junctions and of the zonulin receptor binding domain J Biol Chem 2001 276 19160 19165 10.1074/jbc.M009674200 11278543

[B447] Blaser MJ *Helicobacter pylori* and the parthenogenesis of gastroduodenal inflammation J Infect Dis 1990 161 626 633 10.1093/infdis/161.4.626 2181029

[B448] Miller VL Taylor RK Mekalanos JJ Cholera toxin transcriptional activator toxR is a transmembrane DNA binding protein Cell 1987 48 271 279 10.1016/0092-8674(87)90430-2 3802195

[B449] Childers BM Klose KE Regulation of virulence in *Vibrio cholerae*: the ToxR regulon Future Microbiol 2007 2 335 344 10.2217/17460913.2.3.335 17661707

[B450] Lanka E Wilkins BM DNA processing reactions in bacterial conjugation Annu Rev Biochem 1995 64 141 169 10.1146/annurev.bi.64.070195.001041 7574478

[B451] Iredell JR Manning PA The toxin-coregulated pilus of *Vibrio cholerae* 01: a model for type A pilus biogenesis? Trends Microbiol 1994 2 187 192 10.1016/0966-842X(94)90109-I 7916248

[B452] Kim Y Wang X Zhang X-S Grigoriu S Page R Peti W Wood TK *Escherichia coli* toxin/antitoxin pair MqsR/MqsA regulate toxin CspD Environ Microbiol 2010 12 1105 1121 10.1111/j.1462-2920.2009.02147.x 20105222 PMC3980499

[B453] Yamanaka K Zheng W Crooke E Wang YH Inouye M CspD, a novel DNA replication inhibitor induced during stationary phase in *Escherichia coli* Mol Microbiol 2001 39 1572 1584 10.1046/j.1365-2958.2001.02345.x 11260474

[B454] French CT Panina EM Yeh SH Griffith N Arambula DG Miller JF The *Bordella* type III secretion system effector BteA contains a conserved N-terminal motif that guides bacterial virulence factors to lipid rafts Cell Microbiol 2009 11 1735 1749 10.1111/j.1462-5822.2009.01361.x 19650828 PMC2788067

[B455] Espinosa A Alfano JR Disabling surveillance: bacterial type III secretion effectors that suppress innate immunity Cell Microbiol 2004 6 1027 1040 10.1111/j.1462-5822.2004.00452.x 15469432

[B456] Deane JE Roversi P Cordes FS Johnson S Kenjale R Daniell S Booy F Picking WD Picking WL Blocker AJ Lea SM Molecular model of a type III secretion system needle: implication for host cell sensing Proc Natl Acad Sci U S A 2006 103 12529 12533 10.1073/pnas.0602689103 16888041 PMC1567912

[B457] Jungo F Bougueleret L Xenarios I Poux S The UniProtKB/Swiss-Prot Tox-Prot program: a central hub of integrated venom protein data Toxicon 2012 60 551 557 10.1016/j.toxicon.2012.03.010 22465017 PMC3393831

[B458] Chang D Duda TF Jr Extensive and continuous duplication facilitates rapid evolution and diversification of gene family Mol Biol Evol 2012 29 2019 2029 10.1093/molbev/mss068 22337864

[B459] Wong ESW Belov K Venom evolution through gene duplication Gene 2012 496 1 7 10.1016/j.gene.2012.01.009 22285376

[B460] Moura-da-Silva AM Furlan MS Caporrino MC Grego KF Portes-Junior JA Clissa PB Valente RH Magalhães GS Diversity of metalloproteinases in *Bothrops neuwiedi* snake venom transcripts: evidences for recombination between different classes of SVMPs BMC Genet 2011 12 94 22044657 10.1186/1471-2156-12-94 PMC3217872

[B461] Dutertre S Jin AH Kaas Q Jones A Alewood PF Lewis RJ Deep venomics reveals the mechanism for expanded peptide diversity in cone snail venom Mol Cell Proteomics 2012 12 312 329 23152539 10.1074/mcp.M112.021469 PMC3567856

[B462] Starcevic A Long PF Diversification of animal venom peptides - were jellyfish amongst the first combinatorial chemists? ChemBiochem 2013 10.1002/cbic.201300305 23821453

[B463] Fabricius KE Dubinsky Z, Stambler N Factors determining the resistance of coral reefs to eutrophication: A review and conceptual model Coral Reefs: An Ecosystem in Transition 2011 Heidelberg: Springer Dordrecht 493 508

[B464] Ferrier-Pagès C Gatusso J-P Dallot S Jaubert J Effect of nutrient enrichment on growth and photosynthesis of the zooxanthellate coral *Stylophora pistillata* Coral Reefs 2000 19 103 113 10.1007/s003380000078

[B465] Kramarsky-Winter E Downs CA Downs A Loya Y Cellular responses in the coral *Stylophora pistillata* exposed to eutrophication from fish mariculture Evol Ecol Res 2009 11 1 21

[B466] Goldstone JV Hamdoun A Cole BJ Howard-Ashby M Nebert DW Scally M Dean M Epel D Hahn ME Stegeman JJ The chemical defensome: environmental sensing and response genes in the *Strongylocentrotus purpuratus* genome Dev Biol 2006 300 366 384 10.1016/j.ydbio.2006.08.066 17097629 PMC3166225

[B467] Sarasquete C Segner H Cytochrome P4501A (CYP1A) in teleostean fishes. A review of immunohistochemical studies Sci Total Environ 2000 247 313 332 10.1016/S0048-9697(99)00500-8 10803558

[B468] Gassman NJ Kennedy CJ Cytochrome P-450 content and xenobiotic metabolizing enzyme activity in the scleractinian coral, *Favia fragum* (Esper) Bull Mar Sci 1992 50 320 330

[B469] Garcia E Ramos R Bastidas C Presence of cytochrome P450 in the Carribean corals *Siderastrea sidereal* and *Montastaea faveolata* Cinc Mar 2005 31 23 30

[B470] Ramos R Garcia E Induction of mixed-function oxygenase system and antioxidant enzymes in the coral *Montastraea faveolata* exposure to benzo(*a*)pyrene Comp Biochem Physiol C. Toxicol Pharmacol 2007 144 348 355 10.1016/j.cbpc.2006.11.006 17208054

[B471] Rougée L Downs CA Richmond RH Ostrander GK Alteration of normal cellular profiles in the scleractinian coral (*Pocillopora damicornis*) following laboratory exposure to fuel oil Environ Toxicol Chem 2006 25 3181 3187 10.1897/05-510R2.1 17220087

[B472] Goldstone JV Environmental sensing and response genes in Cnidaria: the chemical defensome in the sea anemone *Nematostella vectensis* Cell Biol Toxicol 2008 24 483 502 10.1007/s10565-008-9107-5 18956243 PMC2811067

[B473] Khanal SK Xie B Tompson ML Sung S Ong SK Van Leeuwent J Fate, transport and biodegradation of natural estrogens in the environmental and engineering systems Environ Sci Technol 2006 40 6537 6546 10.1021/es0607739 17144275

[B474] Snyder SA Westerhoff P Yoon Y Sedlak DL Pharmaceuticals, personal care products, and endocrine disruptors in water: Implications for the water industry Environ Eng Sci 2003 20 449 469 10.1089/109287503768335931

[B475] Goksøyr A Endocrine disruptors in the marine environment: mechanisms of toxicity and their influence on reproductive processes in fish J Toxicol Environ Health A 2006 69 175 184 10.1080/15287390500259483 16291569

[B476] Tarrant AM Atkinson MJ Atkinson S Effects of steroidal estrogens on coral growth and reproduction Mar Ecol Prog Ser 2004 269 121 129

[B477] Hayes JD Flanagan JU Jowsey IR Glutathione transferases Annu Rev Pharmacol Toxicol 2005 45 51 88 10.1146/annurev.pharmtox.45.120403.095857 15822171

[B478] Karntanut W Pascoe D The toxicity of copper, cadmium and zinc to four different *Hydra* (Cndaria: Hydrozoan) Chemosphere 2002 47 1059 1064 10.1016/S0045-6535(02)00050-4 12137038

[B479] Gadelha JR Ferreira VAM Abreu SN Soars AMVM Morgado FMR Hamamura N, Suzuki S, Mendo S, Barroso CM, Iwata H, Tanabe S Experimental mercury bioaccumulation trends in sea anemone *Actinia equina* exposed to chlor-alkali industry effluent contaminated water Interdisciplinary Studies on Environmental Chemistry - Biological Responses to Chemical Contaminants: From Molecular to Community Level. Volume 3 2010 Tokyo: Terrapub 149 157

[B480] Alutoin S Boberg J Nyström M Tedengren M Effects of the multiple stressors copper and reduced salinity on the metabolism of the hermatypic coral *Porites lutea* Mar Environ Res 2001 52 289 299 10.1016/S0141-1136(01)00105-2 11570808

[B481] Nyström M Nordemar I Tedengren M Simultaneous and sequential stress from increased temperature and copper on the metabolism of the hermatypic coral Porites *cylindrica* Mar Biol 2001 138 1225 1231 10.1007/s002270100549

[B482] Mitchelmore CL Verde EA Weis VM Uptake and partitioning of copper and cadmium in the coral *Pocillopora damicornis* Aquat Toxicol 2007 85 48 56 10.1016/j.aquatox.2007.07.015 17804091

[B483] Bielmyer GK Grosell M Bhagooli R Baker AC Langdon C Gillette P Capo TR Differential effects of copper on three species of scleractinian corals and their algal symbionts (*Symbiodinium* spp.) Aquat Toxicol 2010 97 125 133 10.1016/j.aquatox.2009.12.021 20089320

[B484] Bastidas C Garcia EM Sublethal effects of mercury and its distribution in the coral *Porites astreoides* Mar Ecol Prog Ser 2004 267 133 143

[B485] Farina O Ramos R Bastidas C García E Biochemical response of cnidarian larvae to mercury and benzo(a)pyrene exposure Bull Environ Contam Toxicol 2008 81 553 557 10.1007/s00128-008-9534-2 18820822

[B486] Negri AP Howard AJ Inhibition of coral fertilisation and larval metamorphosis by tributyltin and copper Mar Environ Res 2001 51 17 27 10.1016/S0141-1136(00)00029-5 11125701

[B487] Reichelt-Brushett AJ Harrison PL The effect of selected trace metals on the fertilzation success of several scleractinian coral species Coral Reefs 2005 24 524 534 10.1007/s00338-005-0013-5

[B488] Victor S Richmond RH Effect of copper on fertilisation success in the reef coral *Acropora surculosa* Mar Pollut Bull 2005 50 1448 1451 10.1016/j.marpolbul.2005.09.004 16271374

[B489] Jones RJ Zooxanthellae loss as a bioassay for assessing stress in corals Mar Ecol Prog Ser 1997 149 163 171

[B490] Reitzel AM Sullivan JC Traylor-Knowles N Finnerty JR Genomic survey of candidate stress-response genes in the estuarine anemone *Nematostella vectensis* Biol Bull 2008 214 233 254 10.2307/25470666 18574101

[B491] Steele RA Opella SJ Structures of the reduced and mercury-bound forms of MerP, the periplasmic protein from the bacterial mercury detoxification system Biochemistry 1997 36 688 6895 10.1021/bi9621829 9188683

[B492] Mukhopadhyay R Rosen BP Arsenate reductases in prokaryotes and eukaryotes Environ Health Perspect 2002 110 745 748 10.1289/ehp.02110s5745 12426124 PMC1241237

[B493] Hyakawa T Kobayashi Y Cui X Hirano S A new metabolic pathway of arsenite:arsenic-glutathione complexes are substrates for human arsenic methyltransferase Cyt19 Arch Toxicol 2005 79 183 191 10.1007/s00204-004-0620-x 15526190

[B494] Price RE London J Wallaschläger D Ruiz-Chancho MJ Pichler T Enhanced bioaccumulation and biotransformation of As in coral reef organisms surrounding a marine shallow-water hydrothermal vent system Chem Geol 2012 10.1016/j.chemgeo.2012.02.023

[B495] David CP Heavy metal concentrations in growth bands of corals: a record of mine tailings input through time (Marinduque Island, Philippines) Mar Poll Bull 2003 46 187 196 10.1016/S0025-326X(02)00315-6 12586114

[B496] Pessi G Hass D Transcriptional control of the hydrogen cyanide biosynthetic genes *hcnABC* by the anaerobic regulator ANR and the quorum-sensing regulators LasR and RhlR in *Pseudomonas aeruginosa* J Baceriol 2000 182 6940 6949 10.1128/JB.182.24.6940-6949.2000 PMC94819 11092854

[B497] Bird A Perceptions of epigenetics Nature 2007 447 396 398 10.1038/nature05913 17522671

[B498] Jablonka E Lamb MJ Epigenetic inheritance in evolution J Evol Biol 1998 11 159 183 10.1007/s000360050073

[B499] Mirouze M Paszkowski J Epigenetic contribution to stress adaptation in plants Curr Opin Plant Biol 2011 14 267 274 10.1016/j.pbi.2011.03.004 21450514

[B500] Strahl BD Allis CD The language of covalent histone modifications Nature 2000 403 41 45 10.1038/47412 10638745

[B501] Wang M Mok MW Harper H Lee WH Min J Knapp S Oppermann U Marsden B Schapira M Structural genomics of histone tail recognition Bioinformatics 2010 26 2629 2630 10.1093/bioinformatics/btq491 20739309 PMC2951094

[B502] Goll MG Bestor TH Eukaryotic cytosine methyltransferases Annu Rev Biochem 2005 74 481 514 10.1146/annurev.biochem.74.010904.153721 15952895

[B503] Slokin RK Martienssen R Transposable elements and the epigenetic regulation of the genome Nat Rev Genet 2007 8 272 285 17363976 10.1038/nrg2072

[B504] Wolffe AP Jones PL Wade PA DNA demethylation Proc Natl Acad Sci U S A 1999 96 5894 5896 10.1073/pnas.96.11.5894 10339513 PMC34201

[B505] Ooi SK Bestor TH The colorful history of active DNA demethylation Cell 2008 133 1145 1148 10.1016/j.cell.2008.06.009 18585349

[B506] Zhu JK Active DNA demethylation mediated by DNA glycosylases Annu Rev Genet 2009 43 143 166 10.1146/annurev-genet-102108-134205 19659441 PMC3137514

[B507] Branco MR Ficz G Reik W Uncovering the role of 5-hydroxymethylcytosine in the epigenome Nat Rev Genet 2011 13 7 13 22083101 10.1038/nrg3080

[B508] Guo JU Su Y Zhong C Ming GL Song H Hydroxylation of 5-methylcytosine by TET1 promotes active DNA demethylation in the adult brain Cell 2011 145 423 434 10.1016/j.cell.2011.03.022 21496894 PMC3088758

[B509] Roberts SB Gavery MR Is there a relationship between DNA methylation and phenotypic plasticity in invertebrates? Front Physiol 2012 2 116 22232607 10.3389/fphys.2011.00116 PMC3249382

[B510] Brown BE Cossins AR Dubinski Z, Stambler N The potential for temperature acclimatisation of reef corals in the face of climate change Coral Reefs: And Ecosystem in Transition 2011 Berlin: Springer-Verlag 421 433

[B511] Gilbert SF McDonald E Boyle N Buttino N Gyi L Mai M Prakash N Robinson J Symbiosis as a source of selectable epigenetic variation: taking the heat for the big guy Phil Trans R Soc B 2010 365 671 678 10.1098/rstb.2009.0245 20083641 PMC2817139

[B512] Sievers F Wilm A Dineen DG Gibson TJ Karplus K Li W Lopez R McWilliam H Remmert M Söding J Thompson JD Higgins DG Fast, scalable generation of high-quality protein multiple sequence alignments using Clustal Omega Mol Syst Biol 2011 7 539 21988835 10.1038/msb.2011.75 PMC3261699

[B513] Apache-Tomcat[http://tomcat.apache.org/download-70.cgi]

