# Supplementary material for: KEGG orthology-based annotation of the predicted proteome of Acropora digitifera: ZoophyteBase - an open access and searchable database of a coral genome
Source: BMC Genomics. 2013 Jul 26;14:509. doi: 10.1186/1471-2164-14-509 (PMC3750612; doi:10.1186/1471-2164-14-509)
Supplement: Additional file 1: Table S16b — Predicted (UniProt) homologues of animal toxins encoded in the genome of A. digitifera. [file 1471-2164-14-509-S1.docx]

**Supplemental File 1**

**Table 13b (Suppl).** Predicted (UniProt) homologues of animal toxins encoded in the genome of *A. digitifera*.

| **Sequence** | **UniProt Toxin Accession** | **Animal with Closest Homologuey** | **Score** | **e-value** |
| --- | --- | --- | --- | --- |
| v1.01916 | Q92035; Acetylcholinesterase | *Bungarus fasciatus* (Banded Krait) | 778 | 3.52E-94 |
| v1.06761 | Q9IAM1; Agkisacutacin (subunit) (anticoagulant protease) | *Deinagkistrodon acutus* (Sharp-nosed Viper) | 177 | 2.44E-16 |
| v1.04809 | A8QL52; L-Amino acid oxidase | *Bungarus fasciatus* (Banded Krait) | 192 | 1.16E-15 |
| v1.06380 | Q4JHE1; L-Amino acid oxidase | *Pseudechis australis* (Mulga Snake) | 364 | 3.42E-37 |
| v1.10291 | P81383; L-Amino acid oxidase | *Ophiophagus hannah* (King Cobra) | 181 | 3.38E-14 |
| v1.14412 | A6MFL0; L-Amino acid oxidase | *Demansia vestigiata* (Lesser Black Whipsnake) | 178 | 8.38E-15 |
| v1.16469 | P81383; L-amino acid oxidase | *Ophiophagus hannah* (King Cobra) | 164 | 4.60E-12 |
| v1.23477 | P81382; L-Amino acid oxidase | *Calloselasma rhodostoma* (Malayan Pit Viper) | 156 | 4.01E-11 |
| v1.16440 | C5NSL2; Bandaporin (haemolysin) | *Anthopleura asiatica* (Sea Anemone) | 146 | 1.69E-11 |
| v1.16571 | Q76B45 ; Blarina toxin (vasoactive protease) | *Blarina brevicauda* (Northern Short-Tailed Shrew) | 372 | 5.74E-41 |
| v1.06055 | Q593B6; Coagulation factor V | *Pseudonaja textilis* (Eastern Brown Snake) | 565 | 3.36E-63 |
| v1.07831 | P14530; Coagulation factor IX | *Protobothrops flavoviridis* (Okinawa Habu Snake) | 252 | 7.20E-23 |
| v1.01708 | Q4QXT9; Coagulation factor X | *Tropidechis carinatus* (Rough-Scaled Snake) | 237 | 7.32E-21 |
| v1.09601 | Q93109; Equinatoxin-5 (cytolysin) | *Actinia equina* (Beadlet Anemone) | 439 | 1.50E-53 |
| v1.06821 | Q08169 ; Hyaluronidase | *Apis mellifera* (European Honey Bee) | 483 | 4.50E-54 |
| v1.08924 | I0CME7; Hyaluronidase, Conohyal-Cn1 | *Conus consors* (Singed Cone) | 305 | 1.68E-31 |
| v1.06189 | Q9XZC0; α-Latrocrustotoxin Lt1a (neurotoxin) | *Latrodectus tredecimguttatus* (Mediterranean Black Widow Spider) | 229 | 7.61E-19 |
| v1.02942 | G0LXV8; α-Latrocrustotoxin Lh1a (neurotoxin) | *Latrodectus hasseltii* (Australian Redback Spider) | 437 | 6.24E-44 |
| v1.00644 | Q25338; Δ- Latroinsectotoxin Lt1a (neurotoxin) | *Latrodectus tredecimguttatus* (Mediterranean Black Widow Spider) | 242 | 2.18E-20 |
| v1.07446 | A7X3X3; Lectin, Lectoxin Enh4 (platelet binding) | *Enhydris polylepis* (Macleay's Water Snake) | 146 | 2.28E-12 |
| v1.20653 | A7X3Y6; Lectin, Lectoxin Enh7 (platelet binding) | *Enhydris polylepis* (Macleay's Water Snake) | 172 | 1.54E-14 |
| v1.02561 | A7X3Z4; Lectin, Lectoxin Lio1 (platelet binding) | *Liophis poecilogyrus* (Water Snake) | 174 | 1.08E-14 |
| v1.13597 | A7X3Z7; Lectin, Lectoxin Lio2 (platelet binding) | *Liophis poecilogyrus* (Water Snake) | 171 | 2.64E-14 |
| v1.18386 | A7X413; Lectin, Lectoxin Lio3 (platelet binding) | *Liophis poecilogyrus* (Water Snake) | 151 | 1.18E-12 |
| v1.06094 | A7X406; Lectin, Lectoxin Phi1 (platelet binding) | *Philodryas olfersii* (Green Cobra) | 153 | 2.17E-12 |
| v1.06416 | A7X3Z0; Lectin, Lectoxin Thr1 (platelet binding) | *Thrasops jacksonii* (Black Tree Snake) | 147 | 2.70E-12 |
| v1.17681 | Q6TPG9; Lectin, Mucrocetin (platelet binding) | *Protobothrops mucrosquamatus* (Brown Spotted Pit Viper) | 190 | 3.44E-17 |
| v1.00077 | Q66S03; Lectin, Nattectin (platelet binding) | *Thalassophryne nattereri* (Toad Fish) | 166 | 1.01E-13 |
| v1.12241 | Q71RQ1; Lectin, Stejaggregin-A (platelet binding) | *Trimeresurus stejnegeri* (Bamboo Viper) | 198 | 4.90E-18 |
| v1.02245 | A0FKN6; Metalloprotease, Astacin-like toxin | *Loxosceles intermedia* (Recluse Spider) | 331 | 3.71E-34 |
| v1.03638 | Q90391; Metalloprotease, Atrolysin | *Crotalus atrox* (Western Diamondback Rattlesnake) | 305 | 2.11E-30 |
| v1.13106 | D3TTC2; Metalloproteinase, Atragin | *Naja atra* (Chinese Cobra) | 237 | 1.04E-20 |
| v1.11132 | Q7T1T4; Metalloproteinase, BjussuMP-2 | *Bothrops jararacussu* (Jararacussu Pit Viper) | 210 | 4.43E-18 |
| v1.02168 | O73795; Metalloproteinase, Disintegrin | *Gloydius brevicaudus* (Chinese Mamushi Snake) | 236 | 9.03E-21 |
| v1.06910 | Q7SZE0; Metalloproteinase, Disintegrin | *Gloydius saxatilis* (Rock Mamushi Snake) | 373 | 1.94E-37 |
| v1.22282 | P14530; Metalloproteinase, Disintegrin | *Protobothrops flavoviridis* (Okinawa Habu Snake) | 252 | 7.20E-23 |
| v1.03804 | Q2UXQ5; Metalloproteinase, EoVMP2 | *Echis ocellatus* (West African Carpet Viper) | 425 | 6.83E-44 |
| v1.02016 | Q91511; Mucrofibrase-5, [Hypotensive](http://www.uniprot.org/keywords/KW-0382) serine protease | *Protobothrops mucrosquamatus* (Brown Spotted Pit Viper) | 307 | 6.53E-31 |
| v1.09026 | Q7ZZN8; Natrin-2 (neurotoxin) | *Naja atra* (Chinese Cobra) | 178 | 6.49E-16 |
| v1.04153 | A0ZSK3; Neoverrucotoxin (haemolysin) | *Synanceia verrucosa* (Reef Stone Fish) | 732 | 1.05E-82 |
| v1.12433 | A2VBC4; Phospholipase A1 | *Polybia paulista* (Neotropical Social Wasp) | 203 | 6.99E-18 |
| v1.00019 | Q06478; Phospholipase A1 1 | *Dolichovespula maculata* (Bald-Faced Hornet) | 317 | 2.87E-33 |
| v1.09322 | P0CH47; Phospholipase A1, Magnifin | *Vespa magnifica* (Giant Hornet) | 363 | 1.95E-39 |
| v1.03556 | P53357; Phospholipase A1 2 | *Dolichovespula maculata* (Bald-Faced Hornet) | 217 | 9.78E-21 |
| v1.13015 | D2X8K2; Phospholipase A2 | *Condylactis gigantean* (Giant Caribbean Sea Anemone) | 162 | 9.89E-15 |
| v1.18628 | Q9TWL9; Phospholipase A2, Conodipine-M | *Conus magus* (Magical Cone) | 153 | 4.89E-14 |
| v1.11796 | Q9PUH9; Phospholipase A2, Acidic S9-53F | *Austrelaps superbus* (Lowland Copperhead Snake) | 163 | 7.37E-15 |
| v1.09883 | Q8AXW7; Phospholipase A2, Basic | *Micrurus corallinus* (Painted Coral Snake) | 180 | 2.45E-17 |
| v1.14874 | Q90WA8; Phospholipase A2, Basic 2 | *Bungarus fasciatus* (Banded Krait) | 162 | 1.01E-14 |
| v1.11797 | P20256; Phospholipase A2, Basic PA-12C | *Pseudechis australis* (Mulga Snake) | 276 | 8.47E-32 |
| v1.07278 | Q7SZN0; Prothrombin activator Pseutarin-C | *Pseudonaja textilis* (Eastern Brown Snake) | 254 | 1.17E-21 |
| v1.11045 | P83370; Prothrombin activator Hopsarin-D | *Hoplocephalus stephensii* (Stephen’s Branded Snake) | 150 | 4.12E-11 |
| v1.04104 | Q58L94; Prothrombin activator Notecarin D2 | *Notechis scutatus* (Tiger Snake) | 390 | 1.98E-40 |
| v1.00387 | Q58L90; Prothrombin activator Omicarin C | *Oxyuranus microlepidotus* (Inland Taipan ) | 253 | 1.15E-22 |
| v1.02137 | Q58L91; Prothrombin activator Omicarin C | *Oxyuranus scutellatus* (Coastal Taipan) | 181 | 4.64E-14 |
| v1.00618 | Q58L93; Prothrombin activator Porpharin D | *Pseudechis porphyriacus* (Red-Bellied Black Snake) | 404 | 1.36E-43 |
| v1.09896 | P81428; Prothrombin activator Trocarin D | *Tropidechis carinatus* (Rough-Scaled Snake) | 297 | 2.47E-31 |
| v1.13726 | A6MFK7; Prothrombin activator Vestarin D1 | *Demansia vestigiata* (Lesser Black Whipsnake) | 180 | 2.73E-14 |
| v1.02129 | Q6T269; Protease inhibitor, Bitisilin-3 (neurotoxic) | *Bitis gabonica* (Gaboon Viper) | 200 | 2.38E-18 |
| v1.06980 | Q3SB05; Pseudechetoxin (neurotoxin) | *Pseudonaja textilis* (Eastern Brown Snake) | 164 | 6.27E-13 |
| v1.21284 | D8VNS7; Ryncolin-1 (haemostasis inhibitor) | *Cerberus rynchops* (Dog-Faced Water Snake) | 250 | 2.73E-26 |
| v1.18895 | D8VNS8; Ryncolin-2 (haemostasis inhibitor) | *Cerberus rynchops* (Dog-Faced Water Snake) | 557 | 8.25E-69 |
| v1.14251 | D8VNS9; Ryncolin-3 (haemostasis inhibitor) | *Cerberus rynchops* (Dog-Faced Water Snake) | 583 | 3.74E-66 |
| v1.06759 | D8VNT0; Ryncolin-4 (haemostasis inhinitor) | *Cerberus rynchops* (Dog-Faced Water Snake) | 347 | 1.92E-39 |
| v1.01273 | Q9YGN4; Salmorin toxin ( haemostasis inhibitor) | *Gloydius brevicaudus* (Chinese Mamushi Snake) | 147 | 2.18E-11 |
| v1.09855 | B2DCR8; SE-Cephalotoxin | *Sepia esculenta* (Golden Cuttlefish) | 1154 | 1.36E-13 |
| v1.16247 | O13060; Serine protease, 2A | *Trimeresurus gramineus* (Bamboo Viper) | 307 | 8.52E-31 |
| v1.08397 | Q9DF66; Serine protease, 3 (haemostasis inhibitor) | *Protobothrops jerdonii* (Jerdon's pit viper) | 178 | 6.61E-15 |
| v1.03275 | Q9DG84; Serine protease, Serpentokallikrein-2 (haemostasis inhibitor) | *Protobothrops mucrosquamatus* (Brown Spotted Pit Viper) | 305 | 1.03E-31 |
| v1.16638 | Q7SYF1; Serine protease, Cerastocytin (platelet binding) | *Cerastes cerastes* (Saharan Horned Viper) | 207 | 4.19E-20 |
| v1.22320 | P0C5B4; Serine protease, Gloshedobin (platelet binding) | *Gloydius shedaoensis* (Shedao Pit Viper) | 294 | 7.37E-30 |
| v1.15074 | B2D0J4; Serine protease, Venom dipeptidyl peptidase 4 | *Apis mellifera* (European Honey Bee) | 289 | 1.50E-28 |
| v1.05361 | B6RLX2; Serine protease inhibitor, TCI (neurotoxic) | *Ophiophagus hannah* (King Cobra) | 165 | 3.02E-15 |
| v1.10994 | B7S4N9; Serine protease inhibitor, Taicatoxin (neurotoxic) | *Oxyuranus scutellatus* (Coastal Taipan) | 184 | 8.97E-18 |
| v1.11218 | Q90WA0; Serine protease inhibitor, Textilinin-2 (thrombin inhibitor) | *Pseudonaja textilis* (Eastern Brown Snake) | 183 | 1.19E-18 |
| v1.17856 | Q8T3S7; Serine protease inhibitor, U1-aranetoxin-Av1a (neurotoxic) | *Araneus ventricosus* (Devil Spider) | 187 | 3.51E-18 |
| v1.04154 | Q98989; Stonustoxin (haemostasis inhibitor) | *Synanceia horrida* (Estuarine Stonefish) | 267 | 5.94E-24 |
| v1.09427 | Q76DT2; Toxin AvTX-60A (cytolysin) | *Actineria villosa* (Okinawan Sea Anemone) | 741 | 1.77E-91 |
| v1.12311 | Q9GV72; Toxin CrTX-A (haemolysin) | *Carybdea rastonii* (Jimble Jellyfish) | 226 | 4.80E-20 |
| v1.07546 | P58911; Toxin PsTX-60 (haemolysin) | *Phyllodiscus semoni* (Night Anemone) | 613 | 1.46E-72 |
| v1.11270 | E2IYB3; Veficolin-1 (complement activator) | *Varanus komodoensis* (Komodo Dragon) | 250 | 9.68E-25 |
| v1.02115 | Q98993; Verrucotoxin (cytolysin) | *Synanceia verrucosa* (Reef Stonefish) | 521 | 2.05E-55 |
